# Domainoid: domain-oriented orthology inference

**DOI:** 10.1186/s12859-019-3137-2

**Published:** 2019-10-28

**Authors:** Emma Persson, Mateusz Kaduk, Sofia K. Forslund, Erik L. L. Sonnhammer

**Affiliations:** 1 Department of Biochemistry and Biophysics, Science for Life Laboratory, Stockholm University, Box 1031, 17121 Solna, Sweden; 2 Experimental and Clinical Research Cente, a joint cooperation of Max-Delbrück Center for Molecular Medicine and Charité-Universitätsmedizin Berlin, 13125 Berlin, Germany; 3 European Molecular Biology Laboratory, Structural and Computational Biology Unit, 69117 Heidelberg, Germany

**Keywords:** Orthology, Domain ortholog, Protein domain

## Abstract

**Background:**

Orthology inference is normally based on full-length protein sequences. However, most proteins contain independently folding and recurring regions, domains. The domain architecture of a protein is vital for its function, and recombination events mean individual domains can have different evolutionary histories. It has previously been shown that orthologous proteins may differ in domain architecture, creating challenges for orthology inference methods operating on full-length sequences. We have developed Domainoid, a new tool aiming to overcome these challenges faced by full-length orthology methods by inferring orthology on the domain level. It employs the InParanoid algorithm on single domains separately, to infer groups of orthologous domains.

**Results:**

This domain-oriented approach allows detection of discordant domain orthologs, cases where different domains on the same protein have different evolutionary histories. In addition to domain level analysis, protein level orthology based on the fraction of domains that are orthologous can be inferred. Domainoid orthology assignments were compared to those yielded by the conventional full-length approach InParanoid, and were validated in a standard benchmark.

**Conclusions:**

Our results show that domain-based orthology inference can reveal many orthologous relationships that are not found by full-length sequence approaches.

**Availability:**

https://bitbucket.org/sonnhammergroup/domainoid/

## Background

Genes descended from the same ancestral sequence, diverging from each other through speciation, are usually referred to as orthologs [1]. These are often useful in predicting function of experimentally uncharacterized genes in other species, as they are more likely to have retained ancestral functionality than paralogous genes, which instead diverged through gene duplication [2].

**Fig. 1:**
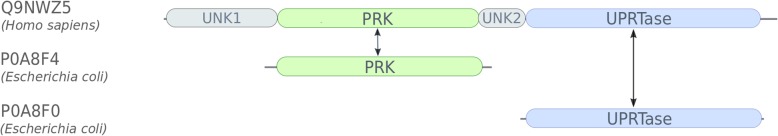
Example of orthologs that may be missed by full-length approaches because of their domain architecture, but not by domain-based orthology inference. Orthologous domains are marked with double arrows. Identifiers are UniProt accessions and Pfam domains (shown as coloured boxes) respectively

Most orthology inference methods, such as OMA [3], EggNOG [4], InParanoid [5], and Hieranoid [6] make no use of the fact that proteins often are composed of multiple domains, and operate on the complete protein level. Protein domains are subsequences of the protein that can independently fold, contribute to function, and move throughout evolution, as evidenced by them being found either in combination with other domains, or alone [7]. A domain-oriented approach for inferring orthology would have several advantages. If domains on the same protein chain are orthologous to different genes [8], this could cause confounding signals and confuse a full-length sequence approach. Separating the protein sequences into domains may offer a clearer view of the orthology in such cases [9, 10]. Even though orthologs tend to have the same domain architecture, it has been estimated that up to 10% of all orthologs between two species could have different domain architectures [11]. This estimation was done considering already well-defined domains, and also considering other types of regions would potentially increase this fraction further. An example of domain orthologs, i.e. proteins that are orthologous on the domain level, but may be missed by full-length sequence approaches, is shown in Fig. 1.

To apply a domain-aware approach for orthology inference one can either use an unsupervised algorithm for domain detection [12] or employ a domain dictionary such as Pfam [13] to divide sequences into domains before the orthology inference. No matter how this is done, the crucial algorithmic change is to treat domains rather than full-length proteins as the operating objects for the algorithm. The idea of domain-based orthology analysis have existed for some time. The need for methods that are aware of different domain architectures for finding homologs has been widely acknowledged, and many domain architecture similarity measures have been tested [14–16]. This growing interest in domain-aware methods highlights the importance of accounting for diversity in domain architectures also among orthologs.

As examples of such previous work, domain-based orthology detection is used by the microbial genome database MBGD [17] which utilizes the DomClust and DomRefine algorithms for constructing ortholog groups at the domain level and for inferring domains. The now retired PHOG [18] database used PhyloFacts trees to infer orthologs, and then FlowerPower to decide whether orthologs had the same Pfam domain architecture. The approach suggested here differs from the MBGD approach by using well-defined Pfam domains, while MBGD, and the DomClust algorithm initiates the analysis with the full-length sequence, makes domains out of clusters when required, and results in ortholog pairs mainly on a protein level. It is difficult to make comparisons to PHOG because it is currently unavailable, but one major difference is that our method uses efficient graph-based ortholog detection. A practical difference is that we provide our method as a standalone software package. Moreover, our approach infers orthology on the single domain level, whereas previous methods have primarily aimed at finding orthologs with the same domain architecture. Domain information has also been used in orthology inference to speed up all-versus-all comparison, by only comparing proteins with similar domain architectures [19, 20].

We here present a novel domain-aware approach, based on letting the InParanoid [5] algorithm identify orthology between domain sequences. The tool, called Domainoid, first extracts domain sequences from the proteomes, and then runs these sequences through the InParanoid algorithm. We analyzed the resulting orthologous domains for discordant domain orthologs, where different domains on the same protein have different evolutionary histories. They are also used to identify orthologs on the full protein sequence level, and we show that Domainoid can find domain orthologs that are not detectable by full-length approaches. To characterize the orthologs found by Domainoid, we evaluate their overlap with full-length orthologs inferred by conventional InParanoid analysis. We further examine the orthologs inferred by Domainoid in a standardized benchmarking.

## Results

### Extraction of domain sequences

In the first step of running Domainoid, domain sequences are extracted from the proteomes using Pfam. For our selected proteomes, *Escherichia coli*, containing 4313 sequences and *Homo sapiens*, containing 21,008 sequences, this first step resulted in a total of 85,551 domain or domain-equivalent sequences. Out of these, 271 protein sequences from *Escherichia coli* and 1932 from *Homo sapiens* had no domain assignments, hence these were fully included as potential single (orphan) domain sequences. Interdomain regions longer than 30 aa that were not assigned with any domain by Pfam, were included as domains labeled as unknown, together with a consecutive number, eg “UNK1”. For *Escherichia coli* 3271 and for *Homo sapiens* 32,783 such domains were included. Domain sequences classified by Pfam as repeats were excluded, 148 for *Escherichia coli* and 4413 for *Homo sapiens.* Domains overlapping with more than half of its length were excluded, 47 for *Escherichia coli* and 112 for *Homo sapiens*. The resulting dataset included 9461 domain or domain-equivalent sequences for *Escherichia coli* and 76,090 sequences for *Homo sapiens*. Running InParanoid on the domain sequences resulted in 2272 orthologous domains, generated from 894 ortholog groups.

### Discordant Orthology

In order to assess the usefulness of orthologous domains generated when running InParanoid on the domain equivalent sequences, we looked for cases where the domain-based approach for orthology inference resulted in orthologous pairs not found by the full-length approach. To identify cases where discordant domain orthologies, i.e. orthologous pairs where gene fission/fusion or domain shuffling may have taken place, the resulting set of ortholog pairs from Domainoid were inspected. For the two species *Homo sapiens* and *Escherichia coli*, Domainoid found 2126 orthologous proteins based on the predicted orthologs of which 1671 had exactly one orthologous domain, while the remaining 455 proteins had multiple orthologous domains. To give a conservative estimate of how many of these may represent cases of discordant domain orthology, we defined primary protein sequences among the proteins with multiple orthologous domains, and secondary proteins as sequences containing domains orthologous to one or more domains in the primary protein. If a primary protein had secondary proteins with different subsets of orthologous domains to the primary protein, this would indicate discordant domain orthology. We found 92 such primary proteins between *Homo sapiens* and *Escherichia coli*, which represent cases of different evolutionary histories for different domains on the same protein. For a complete listing of the discordant domain orthologs identified, see Additional file 2.

Out of the discordant domain orthologs, 261 protein pairs could be generated, and from these, 170 protein pairs could not be identified by the full-length approach inParanoid when run using default settings. An example of such a case is shown in Fig. 1, where the protein Q9NWZ5, Uridine-cytidine kinase-like 1, from *Homo sapiens* with four domains, Pfam domains PRK (Phosphoribulokinase/Uridine kinase) and UPRTase (Uracil phosphoribosyltransferase), and two interdomain, unannotated regions labeled UNK1 and UNK2, have one orthologous domain on each of the *Escherichia coli* proteins P0A8F4, Uridine kinase, and P0A8F0, Uracil phosphoribosyltransferase. The sequence for protein P0A8F4 almost entirely consists of the domain PRK, and protein P0A8F0 almost entirely of the domain URPTase. The *Homo sapiens* protein Q9NWZ5 is involved in catalyzation of UPM biosynthesis in the salvage pathway, similar to the *Escherichia coli* proteins identified as domain orthologs. The orthologous domains, PRK and UPRTase have been shown to appear in different constellations in different species, either in separate proteins or in one protein chain. It has previously been shown that the appearance of these domains in separate proteins, as in *Escherichia coli* implies a functional dependency between the proteins, where the absence of one of them resulted in growth inhibition [21]. This discordant orthology could represent a case of gene fusion, where the *Escherichia coli* proteins have been merged with interdomain regions into *Homo sapiens* protein Q9NWZ5. The orthology of these domains is supported by the Neighbor-joining trees found in Additional file 1: Figure S1 and Additional file 1: Figure S2, where they are adjacent among the closest homologs found when running BLAST on the proteomes for the respective domains. None of these protein pairs could be identified as orthologs by InParanoid, Hieranoid, or OMA.

The inability of InParanoid to recognize these discordant domain orthologs can to some extent be explained by the length filter, by default dismissing orthologous proteins that have a matching sequence shorter than half of the length of the longest sequence. Running InParanoid with sequence overlap cutoff of 0 and a segment coverage cutoff of 0 does detect one of the pairs, Q9NWZ5/P0A8F4, but the other pair remains undetected.

**Fig. 2:**
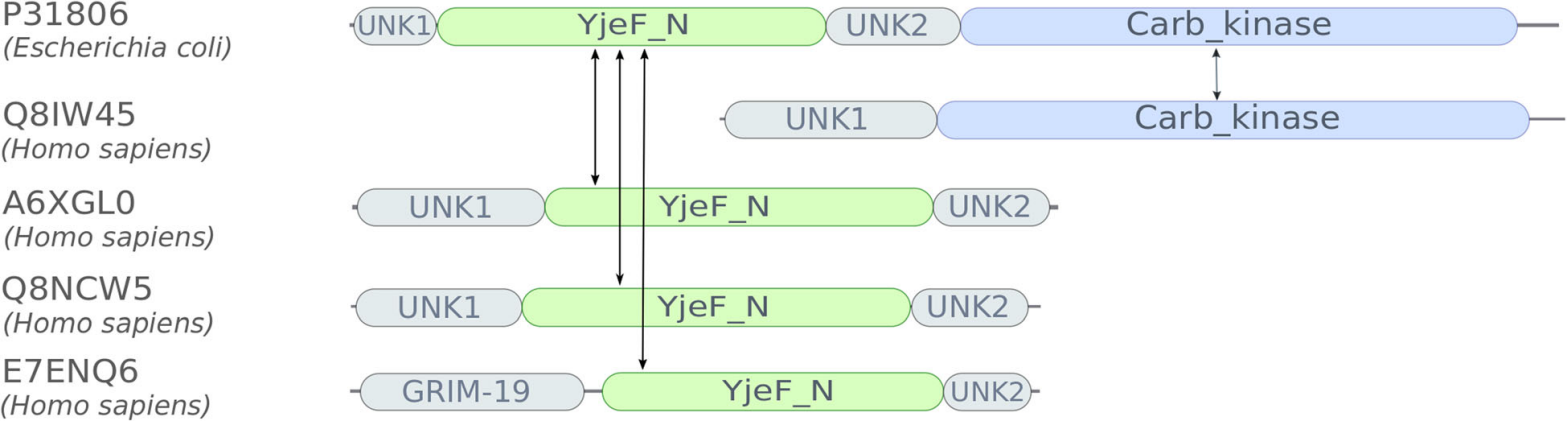
Example of a common scenario for discordant orthologies discovered by Domainoid, where the primary protein has more than two secondary proteins. The YjeF_N and Carb_kinase domains are both involved in NAD(P) H dehydration, where the former domain performs epimerization. The two domains have different evolutionary histories, causing discordant domain orthology as revealed by Domainoid. Identifiers are UniProt accessions and Pfam domains (shown as coloured boxes) respectively

Among the discordant domain orthologs generated, 51 out of 92 proteins defined as primary had more than two secondary proteins. Among these cases, many display a behaviour where two or more of the secondary proteins are very similar in its domain architecture, and contain the same orthologous domains to the primary protein, while other secondary proteins contain different orthologous domains to the primary protein. One example of this is P31806 in *Escherichia coli,* bifunctional NAD(P)H-hydrate repair enzyme Nnr, having four secondary proteins, A6XGL0, YjeF N-terminal domain-containing protein 3, Q8IW45, ATP-dependent (S)-NAD(P)H-hydrate dehydratase, Q8NCW5, NAD(P)H-hydrate epimerase, and E7ENQ6, an uncharacterized protein from *Homo sapiens*. The domain architectures of these proteins are shown in Fig. 2. Three of the *Homo sapiens* proteins, A6XGL0, Q8NCW5, and E7ENQ6 all have the orthologous domain YjeF_N (YjeF-related protein N-terminus), while the fourth, Q8IW45, has one unique domain, orthologous to the second annotated domain in the primary protein, Carb_kinase (Carbohydrate kinase). The *Escherichia coli* protein, P31806, has two functions, epimerization of NAD(P) HX, which is shared by the proteins orthologous to domain YjeF_N, and dehydration of NAD(P) HX, shared by the protein orthologous to the Carb_kinase domain, indicating different evolutionary histories for the domains. None of these ortholog pairs are identified by OMA, Hieranoid or InParanoid with default settings. Running InParanoid with a sequence overlap cutoff of 0 and a segment coverage cutoff of 0 identifies only one of the four pairs, P31806/Q8IW45. The orthology of these domains is supported by the Neighbor-joining trees in Additional file 1: Figure S3 and Additional file 1: Figure S4.

### Inferring domain-based protein ortholog pairs

Domainoid can be used to call full-length protein orthology if more domains than a preselected threshold are orthologous. As InParanoid inferences have been shown to be reliable in benchmarks [22], we used those ortholog calls to calibrate the threshold parameter, called alpha. To this end we ran the conventional InParanoid algorithm on the full-length sequences and compared its resulting ortholog group co-membership pairs to Domainoid’s at different alpha thresholds. Domainoid analysis assigned 85,551 domain sequences to 894 ortholog groups for the two tested species. These consisted of 47,293 sequences with identified Pfam domains, 36,055 unannotated regions larger than 30 residues, and 2203 full-length protein sequences.

**Fig. 3:**
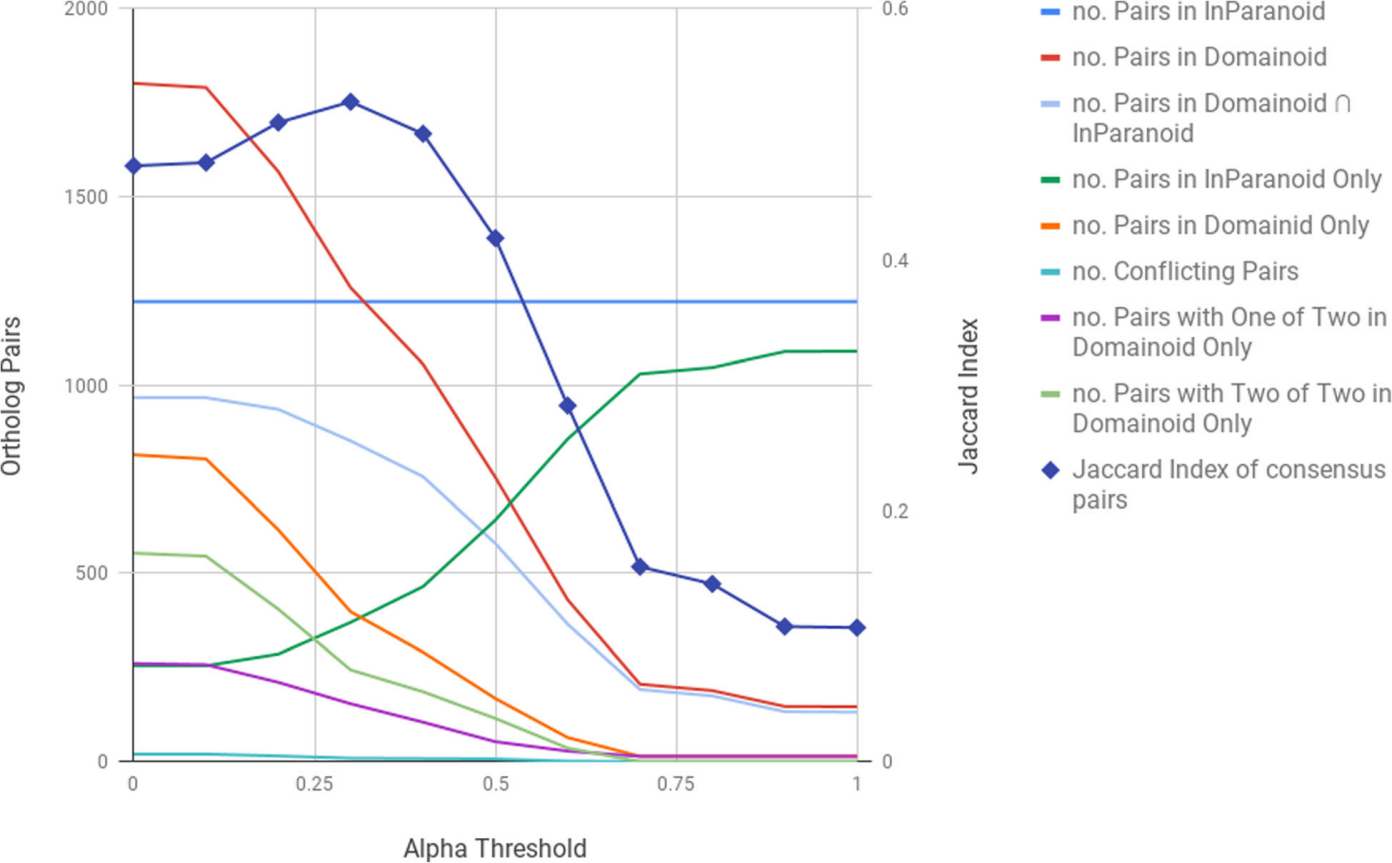
Number of orthologous pairs (left vertical axis) at different alpha thresholds (horizontal axis) when running Domainoid and InParanoid, and Jaccard index (right vertical axis) over consensus pairs at different alpha thresholds on species *Escherichia coli* and *Homo sapiens*. InParanoid-only and Domainoid-only pairs are uniquely found by the respective method

In order to pick an alpha threshold, we ran Domainoid with an alpha threshold at eleven different equally spaced intervals (see Fig. 3). For the Domainoid pairs we wanted to minimize the number of conflicting clusters, under the assumption that InParanoid assignments, where available, will more often be correct, and maximize the agreement. To quantify the consensus between Domainoid and InParanoid, we calculated the Jaccard index, the intersection over the union of the pairs from InParanoid and Domainoid, for alpha thresholds between 0.0 and 1.0. Since the consensus is peaking at an alpha threshold of 0.3, with ~ 53% of the total pairs being found by both methods, this was selected as a suitable alpha threshold.

**Fig. 4:**
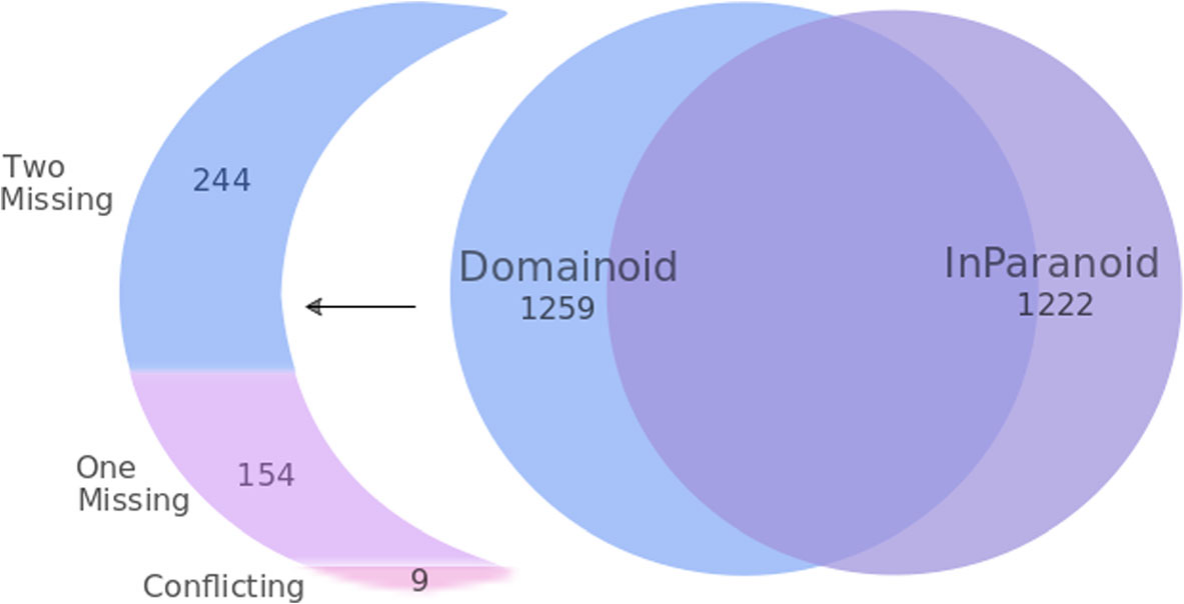
Diagram showing the number of pairs of orthologs inferred for *Escherichia coli* and *Homo sapiens* by Domainoid, InParanoid, and their intersection. Furthermore, the Domainoid-only part is subdivided into three categories, depending on how Domainoid ortholog pairs map onto InParanoid results. “Two missing” means that neither protein in a pair was assigned to an ortholog group by InParanoid, “One missing” indicates that one of the proteins in the pair was not assigned by InParanoid, and “Conflicting groups” are pairs where proteins are assigned to other pairs in InParanoid

On the level of full-length sequence ortholog pairs, there are three main scenarios. InParanoid and Domainoid may both identify the relationship, or it may be found by only one or the other of the methods. InParanoid full-length analysis of the two tested proteomes yielded 1222 ortholog pairs, whereas Domainoid at alpha threshold 0.3 yielded 1259 domain ortholog pairs. We note that InParanoid identified 255 ortholog pairs that could not be found by Domainoid at any threshold. This loss in sensitivity is likely due to the increased difficulty to detect orthology for shorter sequences, as well as the fact that there is an information loss, where similarity on the full-length sequence might be missed when the sequence is split into smaller pieces. Four hundred seven of the domain ortholog pairs were identified by Domainoid only. By mapping these onto ortholog groups resulting from conventional InParanoid analysis we can distinguish 9 conflicting pairs, where both proteins were assigned by InParanoid but in conflicting ortholog groups, 154 domain ortholog pairs with one protein not assigned to an ortholog group in the InParanoid results, and 244 domain ortholog pairs where neither protein was assigned to an ortholog group by InParanoid. This distribution of Domainoid and InParanoid ortholog pairs is shown in Fig. 4. It is possible to lower InParanoid’s coverage cutoffs in order to capture more domain-level orthologs. Setting both cutoffs to zero results in a decrease of the pairs unique to Domainoid from 398 to 225. However, such a low coverage cutoff also produces a large amount of potentially false positives, increasing the total number of orthologs inferred by InParanoid with ~ 39%. Despite a zero cutoff in InParanoid, it still fails to detect many Domainoid-unique ortholog pairs, most likely due to the inability to detect similarity on a sequence where domains are scattered over the protein, separated by long interdomain regions.

### Advantages of Domainoid

To further assess under what conditions a domain-based approach works better than full-length orthology inference with InParanoid, we inspected Domainoid-only protein-level ortholog pairs with one or two proteins missing from conventional InParanoid results when using an alpha threshold of 0.3. Out of 398 such ortholog pairs on the protein level, 374 (~ 94%) had only one orthologous domain.

**Fig. 5:**
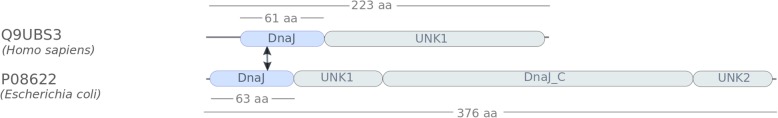
A common scenario for orthologs found by Domainoid but missed by conventional InParanoid analysis involves short orthologous domains. In this example the orthologous domain, the chaperone DnaJ, is small relative to the whole protein. Identifiers are UniProt accessions and Pfam domains (shown as coloured boxes) respectively

Among these single domain pairs, we find many cases similar to the pair Q9UBS3/P08622, *Homo sapiens* protein DnaJ homolog subfamily B member 9, and *Escherichia coli* protein Chaperone protein DnaJ, see Fig. 5, both chaperone proteins involved in protein folding. The orthologous domain, DnaJ, covers a small part of Q9UBS3, which also contains an unannotated region, while P08622 consists of the common domain, followed by the domain DnaJ_C (DnaJ C terminal domain) and two unannotated regions. This pair can not be identified as an ortholog pair by the full-length approach InParanoid when using the default settings, or when running with a sequence overlap cutoff of 0 and a segment coverage cutoff of 0 for the *Homo sapiens/Escherichia coli* dataset. The pair could also not be identified as an ortholog pair in any of the databases OMA or Hieranoid. A Neighbor-joining tree confirming the domains as closest pairs can be found in Additional file 1: Figure S5.

**Fig. 6:**
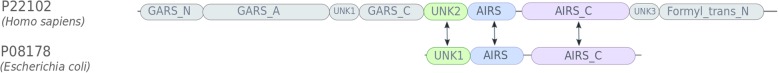
Example of orthologs with multiple orthologous domains identified by Domainoid, but missed by conventional InParanoid. These proteins share one of the functions of the trifunctional *Homo sapiens* protein, namely imidazole synthesis, revealed by orthology inference on a more fine-grained scale than on the full protein sequence level. Identifiers are UniProt accessions and Pfam domains (shown as coloured boxes) respectively

One of the cases where Domainoid identifies ortholog pairs with more than one common domains, is for *Homo sapiens* protein P22102, Trifunctional purine biosynthetic protein adenosine-3 and *Escherichia coli* protein P08178, Phosphoribosylformylglycinamidine cyclo-ligase, see Fig. 6. These proteins consist of three common domains, AIRS (AIR synthase related protein, N-terminal domain), AIRS_C (AIR synthase related protein, C-terminal domain), and one orthologous domain not identified by Pfam, labeled UNK2/UNK1. The AIRS and AIRS_C domains are the n-terminal and c-terminal domains involved in AIR (5-aminoimidizole ribonucleotide) synthase. For the *Escherichia coli* protein P08178 these three domains covers almost the entire sequence, but for the *Homo sapiens* protein P22102, these three domains are surrounded by other named domains as well as interdomain regions. This ortholog pair is not found by OMA, Hieranoid, or InParanoid at any sequence overlap cutoff and segment coverage cutoff. A Neighbor-joining tree supports the AIRS domains as orthologous, see Additional file 1: Figure S6. For the two other domains, no other close homologs could be identified using BLAST with an e-value threshold below zero, indicating that the orthologs for these domains are the closest. Domainoid identified five pairs similar to this, with more than two common domains when run on the *Homo sapiens/Escherichia coli* dataset with an alpha threshold of 0.3. None of these proteins are identified in any ortholog pair by InParanoid, most likely because the similar regions are scattered over the full protein sequence, making it difficult to detect sequence similarity.

### Benchmarking

To get an estimate of the quality of the ortholog pairs generated by Domainoid, the results were benchmarked using the Orthology benchmarking web service on the fungal subset of the Quest For Orthologs reference proteomes. The benchmarking web service provided a result for the fungal subset on the Generalized species tree discordance test when uploading orthologs projected on the protein level. To calibrate the alpha threshold, the results of Domainoid in relation to InParanoid were evaluated at different alpha thresholds, see Additional file 1: Figure S7. An alpha threshold of 0.3 was again used in order to maximise the consensus between the methods, and keep the number of conflicting pairs to a minimum. Since the consensus between the two methods was high on this dataset, a benchmark was performed using Domainoid as a standalone method. However, it is reasonable to believe that a dataset of species having a higher evolutionary distance would have a lower consensus, as seen for the *Homo sapiens/Escherichia coli* dataset, where InParanoid identifies a higher percentage of orthologs not detected by Domainoid. For such cases, the orthologs inferred by InParanoid could be enriched with non-conflicting new orthologs inferred only by Domainoid, to ensure as little loss of information as possible. To get an estimation of the quality of the orthologs inferred using Domainoid to enrich InParanoid orthologs, a benchmark was also performed on the merged set of orthologs for the fungal dataset, and for this merged set of data, a Domainoid alpha threshold of 0.4 was shown to generate the best results (see Additional file 1: Figure S8.

**Fig. 7:**
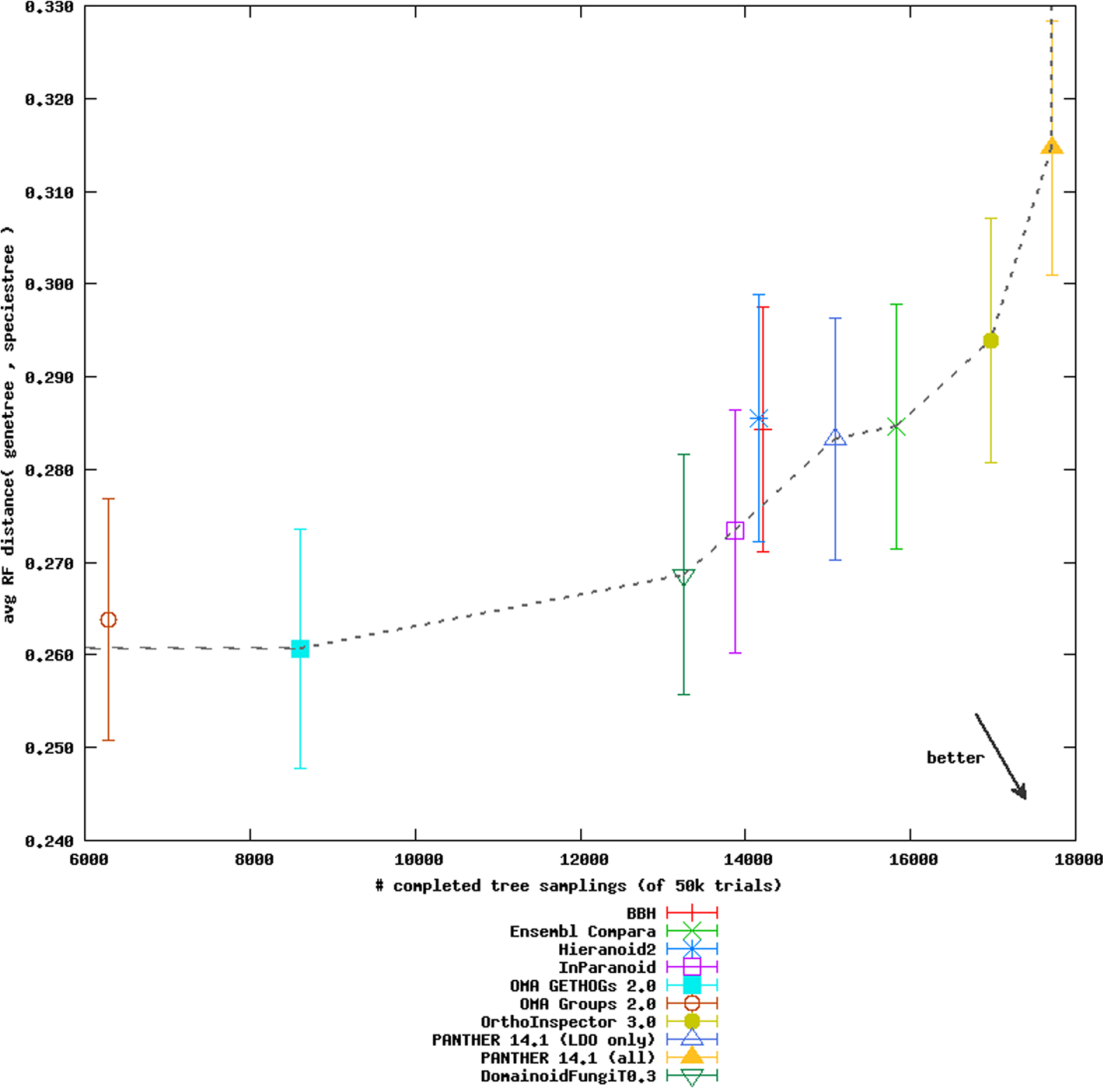
Result of Domainoid compared to publically available methods in the Orthology benchmarking web service for the Generalized species tree discordance benchmark on the fungal subset of the QFO 2018 reference data. The number of completed tree samplings are represented on the horizontal axis and the average RF distance is represented on the vertical axis

The results from the Generalized species tree discordance test from the Orthology benchmarking web service for Domainoid with an alpha threshold of 0.3, InParanoid enriched with Domainoid at alpha threshold 0.4, and conventional InParanoid on the fungal subset of the Quest for Orthologs data can be seen in Table 1. The results of the benchmarking reveals a lower error rate, deviation from species tree and fraction of species tree reconstructions with errors, when using Domainoid compared to conventional InParanoid while the number of uploaded orthologs and the number of inferred trees is slightly lower. The results of the merged set reveal an increase in the number of inferred trees and number of uploaded Orthologs in comparison to the two other methods, where the number of inferred trees is increased by ~ 11% in comparison to conventional InParanoid, while the error rate is slightly higher. The performance of Domainoid at an alpha threshold of 0.3 in the Generalized species tree discordance test in comparison to publically available results from other orthology inference tools can be seen in Fig. 7. This shows that Domainoid retains a balance between the recall, the number of inferred trees and the precision, keeping a low RF distance, placing the method on the Pareto frontier.

**Table 1:** Performance metric assessment results from the Orthology benchmarking web service comparing conventional InParanoid analysis performed on fungal species with corresponding results from Domainoid and Inparanoid enriched with Domainoid. Results are from the Generalized species tree discordance benchmark. This benchmark relies on the axiom that trees of orthologs should reflect species phylogeny, such that disagreement between a trusted species tree and the reconstructed relationships acts as an inverse measure of quality of an orthology reconstruction method. For robustness this is assessed under multiple (usually 50 K iterations) resampling of sets of gene trees from the orthology inference results. From these, measures (average and standard error) of different agreement metrics are taken, with two basic metrics reported. One is Robinson-Foulds (RF) set (Jaccard) distance between splits encoded by species and gene trees (controlling for leaf set divergences). One is simply what fraction of reconstructed trees contain an error when compared to the trusted tree

Method	Inferred Trees	Orthologs	Deviation from species tree (average RF distance)	Fraction species tree reconstructions with error (average)
InParanoid	13,883	254,236	0.273	0.419
Domainoid, alpha threshold 0.3	13,257	248,586	0.269	0.412
InParanoid enriched with Domainoid, alpha threshold 0.4	15,366	274,606	0.279	0.426

## Discussion

In this study we have presented the Domainoid algorithm for domain-oriented orthology inference and characterized its performance relative to conventional full-length protein orthology inference by InParanoid. We found that Domainoid is able to find orthologs not detected by InParanoid, where such detection was prevented by their divergent domain architectures and the different evolutionary history of the constituent domains. The approach taken by Domainoid, to split protein sequences into domains before orthology inference, could be seen as a complement to full-length approaches, and not as their replacement, as the splitting of proteins will in some cases lead to missed orthology relationships. Despite this we could see that Domainoid performed well as a standalone method in comparison to other tools when run on the fungal subset, but that using it to enrich InParanoid orthologs further improves the coverage of inferred trees.

The difference between Domainoid and InParanoid is more conceptual than technical. As InParanoid operates on full-length proteins it applies a sequence coverage cutoff of at least 50%. This is a logical choice for a full-length protein oriented approach. One might suspect that InParanoid could capture some of the orthologs found only by Domainoid if this cutoff was lowered. This is true to some extent, and lowering the cutoff to zero does detect some of the ortholog pairs identified by Domainoid, but the majority of the ortholog pairs remains undetected by InParanoid, presumably because this does not change the fact that InParanoid still operates on the full-length sequence level and prioritizes global matches over domain matches.

In order to compare the domain-oriented approach with the full-length protein approach it was necessary to project orthology of domains onto full-length proteins. This was done on the basis of a cutoff parameter alpha, the fraction of orthologous domains between two proteins. This cutoff can be set to any value between 0 and 1, from the most relaxed to the most stringent setting. We analyzed how it affects the congruence with conventional InParanoid and concluded that an alpha threshold close to 0.3 can be recommended. This retains a high consensus at the same time as it gives minimal conflicts with InParanoid. For other purposes, such as finding more speculative domain orthologs, this cutoff may be lowered. When using Domainoid to enrich orthologs generated by InParanoid, a slightly higher alpha threshold of 0.4 can be recommended.

For the two species *Escherichia coli* and *Homo sapiens* we showed examples of what type of additional orthologous pairs can be found by Domainoid. This revealed that many domain orthologs missed by full-length InParanoid had two-domain architectures where one domain is orthologous but covers less than half of one of the sequences. We validated Domainoid orthologs against tree reconstructions, which confirmed that the domain orthologs were inferred correctly as they are evolutionarily the closest among a set of close homologs. To validate Domainoid in a broader and more general scope, a standard orthology benchmark was performed which revealed that Domainoid performs well in comparison to full-length methods on species having close evolutionary distances, and that using it to enrich InParanoid orthologs further improves the results, generating a higher coverage.

## Conclusions

In this study, we have presented the Domainoid algorithm for inference of orthologs on the domain level. The results of using this domain-based approach suggest that many orthologous relationships are resolvable only at the level of protein domains, and that a domain-based approach can reveal discordant orthology where domains within a protein have different evolutionary histories. Our results show that complementing full-length analysis with domain orthology analysis improves our capability to reconstruct evolutionary relationships.

## Methods

### Software

To implement and test the Domainoid algorithm, the following software packages were used: BLAST 2.2.18 [23], Python 3.4.1 [24], Perl 5.26 [25], InParanoid 4.1 and PfamScan 1.6 with HMMER 3.1b2 [26]. Domainoid can be used packaged together with its dependencies in a Singularity container [27], or as a scripting pipeline requiring these dependencies. The source code, and instructions for running Domainoid can be found in bitbucket (https://bitbucket.org/sonnhammergroup/domainoid/). The tool requires proteomes for at least two species, and Pfam profiles as input, and produces a list of orthologous domains as well as a list of the orthologs projected on the protein level. Domainoid provides an option to merge the resulting ortholog pairs with results from conventional InParanoid, as well as to output a list of potential discordant domain orthologs from the Domainoid analysis.

### Domainoid algorithm

Conventional InParanoid takes as input two multi-FASTA files for two species, performs BLAST search all vs. all and finds best hits of sequences from two species as seed orthologs. BLAST is also performed on each multi-FASTA file against itself, to score close in-paralogs. Additional filters are applied, to ensure that matching sequences have sufficient similarity over their entire sequence length. Low complexity filters are enabled with BLAST searches to filter out regions with little discriminative information. In a BLAST post-processing step, additional filters are applied such as requiring a minimum bitscore (default 40 bits) and filters assuring that all matching segments cover at least 50% of the longer sequence. The best matching pairs of sequences from two species define seed orthologs. Using higher bitscores as a proxy for lower evolutionary distances, additional inparalogs are recruited around the seed ortholog pairs if their bitscore with the seed ortholog in the same species does not fall below the bitscore of the seed pair. Sets of seed orthologs and inparalogs define ortholog groups, within which all pairs of proteins that belong to different species are orthologs. When conventional InParanoid is run on full-length sequences, proteins from different species in the same ortholog groups are considered to be ortholog pairs and those from the same species as in-paralogs (co-orthologs relative to the group members in the other species) [28].

**Fig. 8:**
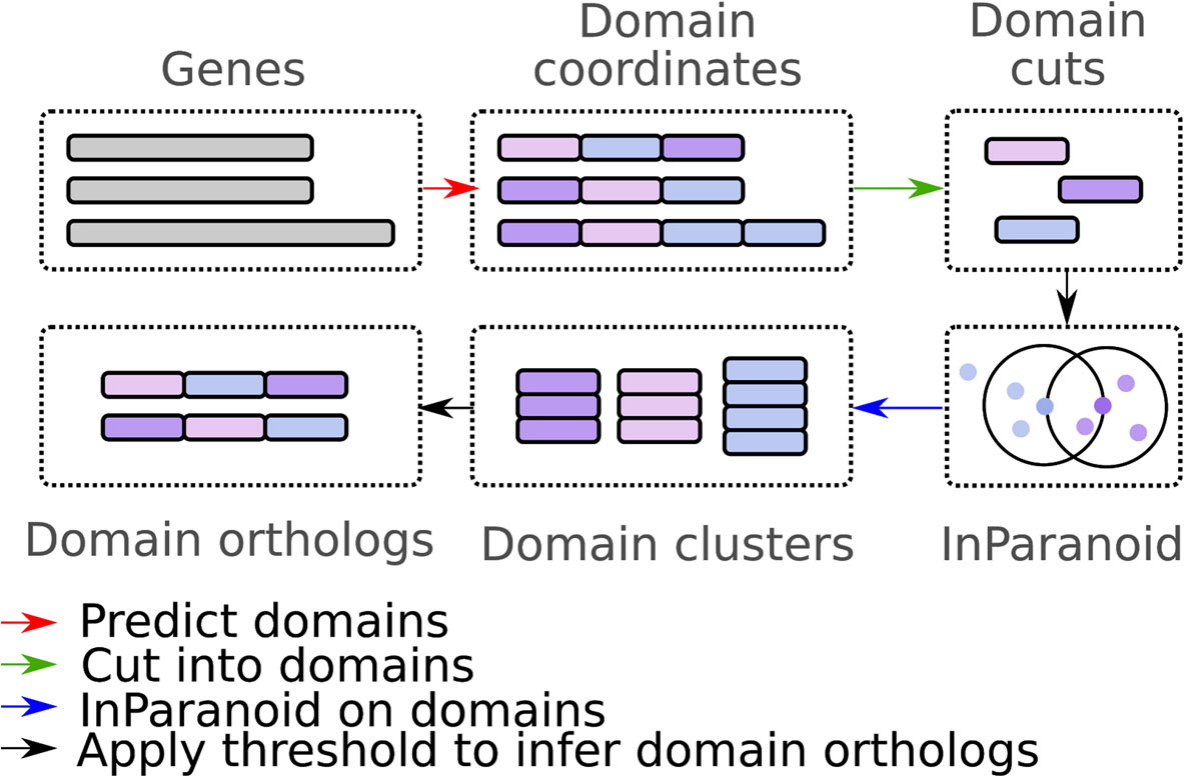
Overview of the Domainoid algorithm

Domainoid differs from conventional InParanoid in the first and last steps of this procedure. Instead of starting with all full-length sequences, most of the sequences are replaced with sets of region cuts corresponding to identified domains. A standard InParanoid is then run with these sequences as input, resulting in ortholog groups containing single domains rather than full-length sequences. These ortholog groups can then either be analyzed on the domain level or be used in aggregation to infer whole-protein ortholog pairs based on how many of their domains are orthologous. A general overview of steps in the Domainoid algorithm is shown in Fig. 8.

### Domain assignment and coverage filtering

For a set of input proteomes, the first step of Domainoid analysis is to identify instances of protein domain families in the sequences. This is done using the PfamScan tool. Additionally, any regions longer than 30 residues that are not matched to a known Pfam domain family are included as potential novel or orphan domain sequences, or potentially remotely diverged domain sequences. These are included in the same way as proper domain hits are in further analysis, whereas shorter unassigned regions are considered more likely to simply be linker regions or terminals and are therefore discarded. Any full-length genes without hits to known domains are similarly included in its complete length as potentially novel domain sequences. Domains identified by Pfam as Repeats are excluded from the set of sequences, and domains that are overlapping with more than half of its length are similarly excluded from the complete set. In cases where two domains are overlapping with more than half of its length, the longest sequence is kept. The resulting joint set of known and potentially novel domain sequences in each of the two species are then analyzed as per the conventional InParanoid pipeline, and Domainoid analysis then proceeds by merging and analyzing the resulting orthology groups pairwise (i.e. always between two species). The results can then either be analyzed as ortholog pairs on the domain level, or be projected on a protein-level as domain orthologs.

### Identifying discordant orthologs

The orthologous domains generated by Domainoid can be used to identify discordant domain orthology, i.e. proteins with domains that are orthologous to different proteins. Such domains have distinct evolutionary histories, resulting from gene fission/fusion or domain shuffling. To identify potential discordant orthologs, we define primary protein sequences as having orthologous domains to more than one other protein, and secondary proteins as sequences in the other species containing domains orthologous to a subset of the domains in the primary protein. If a primary protein has at least two secondary proteins with mutually exclusive orthologous domains to the primary protein, they are considered to be potential discordant orthologs.

### Recovering ortholog pairs

As in a conventional InParanoid analysis, Domainoid’s orthology groups are identified, each thus derived from a single domain-length sequence in the last common ancestral species of the two species analyzed in each case. If a set of proteins have the same domain architectures and the Domainoid ortholog groups corresponding to each of these domains support the same orthology relationship, then it is straightforward to conclude that this relationship also holds for the full-length sequences, as supported by the integrated results of the domain-level analysis.

**Fig. 9:**
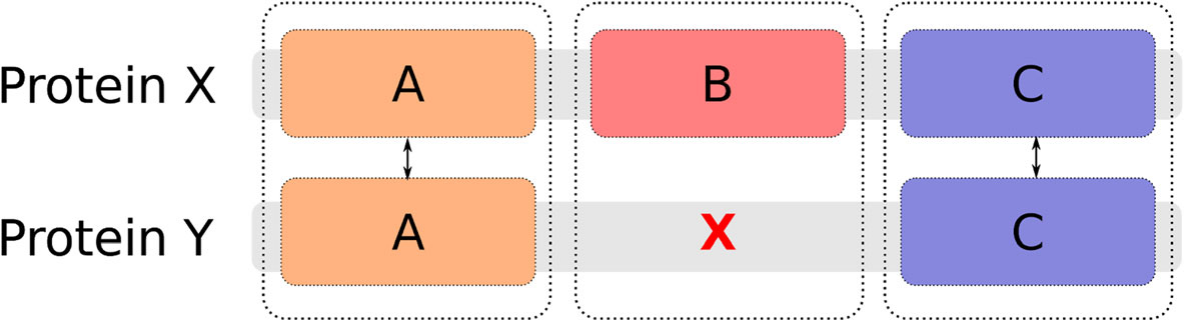
Visualization of how to calculate the alpha threshold. The number of orthologous domains (A and C) for the protein pair {X,Y} is 4, and the total number of domains for that pair is 5. The alpha value for the pair is thus $$ \raisebox{1ex}{$4$}\!\left/ \!\raisebox{-1ex}{$5$}\right. $$

In scenarios where orthology relationships hold for individual domains within a set of proteins, but not for all domains, it is necessary to decide whether full-length sequence proteins are orthologs. This is achieved by defining an alpha threshold parameter. For each pair of potential orthologs (proteins sharing at least one domain), the alpha parameter is calculated by counting the number of domains belonging to the same ortholog group, as assigned by InParanoid, divided by the number of domains these two proteins have in total. If alpha value is at least as large as a predefined threshold then a protein pair is considered orthologous. The calculation is more intuitively depicted in Fig. 9. Each domain or domain-equivalent sequence generated in the domain extraction, including unannotated regions and full-length sequences without domain assignments were subsequently included in this analysis.

### Enrichment of full-length methods

When using Domainoid to recover orthologs on the protein level, there is a risk of missing orthology relationships detectable only in full protein sequences but not with individual domains. In order to compensate for this, Domainoid can be used to complement a set of orthologs generated by conventional InParanoid. To perform this merger, Domainoid and InParanoid are run on the same data, and all orthologs detected by InParanoid are added to the merged set together with the orthologs that are uniquely identified by Domainoid. In cases where orthology assignments are conflicting, i.e. where both proteins are assigned by both methods but in conflicting ortholog groups, only the InParanoid pair is kept, since the orthologs predicted by InParanoid have previously proven to be reliable in benchmarks [22].

### Validation and benchmarking dataset

To assess the validity of Domainoid inferences and for benchmarking our results, we used the curated set of reference proteomes selected within the Quest for Orthologs initiative (QfO v2018–04) [29]. For all proteins, sequence domains were identified by the PfamScan tool [30] using the HMM profiles from version 32.0 of the Pfam database [13]. To avoid benchmarking results being affected by sampling bias, we selected a pair of genomes that had enough evolutionarily distance for significant domain shuffling to have occurred, and where enough structure biology work had been undertaken so that we could be confident in the domain calls. The species *Escherichia coli* and *Homo sapiens* meet these criteria, and were selected for analysis of Domainoid results. The resulting orthologs generated by Domainoid on this dataset were compared to orthologs inferred by InParanoid [5] for the same proteomes.

To perform the benchmark we used a subset of the Quest for Orthologs dataset, the fungal species *Batrachochytrium dendrobatidis*, *Candida albicans*, *Neosartorya fumigata*, *Neurospora crassa*, *Phaeosphaeria nodorum*, *Puccinia graminis*, *Saccharomyces cerevisiae*, *Schizosaccharomyces pombe*, *Sclerotinia sclerotiorum*, *Ustilago maydis*, *Yarrowia lipolytica*, and *Cryptococcus neoformans*. The resulting orthologs between these fungal species were uploaded to the Orthology benchmarking web service [22] which compared the results of the Generalized species tree discordance benchmark to those of other orthology inference tools with publically available results. Since the benchmarking web service evaluates orthologs on the protein level, and not on domain level, the orthologous domains generated by Domainoid were had to be converted to protein level orthologs.

To evaluate pairs of domain orthologs in the examples, we used BLAST 2.2.18 [23] to identify the closest homologs of the orthologous domains within the set of domains for each species. For each example, we selected a BLAST E-value cutoff that enabled the number of identified homologs to be as high as possible, while avoiding inclusion of too distant homologs that resulted in low quality of the alignments. These domains were aligned with Muscle 3.8.31 [31] and the alignments were used to build Neighbor-joining trees with Belvu 4.44.1 [32]. To further evaluate the additional sensitivity demonstrated through these examples, we verified that the pairs could not be found by InParanoid [5], OMA [3] or Hieranoid [6].

## Additional files

**Additional file 1: Figures S1–S6.** containing trees to support the orthology for the examples, **Figure S7.** showing the distribution of orthologs from Domainoid in relation to InParanoid for fungi, and **Figure S8.** showing performance of Domainoid merged with InParanoid in the Generalized species tree discordance test for fungi on different alpha thresholds.

**Additional file 2. **Including a list of discordant domain orthologs for *Homo sapiens/Escherichia coli.*

## Data Availability

The source code for Domainoid can be found at https://bitbucket.org/sonnhammergroup/domainoid/**.** The datasets analysed during the current study are available from the Quest For Orthologs reference proteomes, http://ftp.ebi.ac.uk/pub/databases/reference_proteomes/previous_releases/qfo_release-2018_04/QfO_release_2018_04.tar.gz
