# Supplementary material for: Domainoid: domain-oriented orthology inference
Source: BMC Bioinformatics. 2019 Oct 28;20:523. doi: 10.1186/s12859-019-3137-2 (PMC6816169; doi:10.1186/s12859-019-3137-2)

### Figure S1: NJ Tree of the closest homologs found when running BLAST with an e-value cutoff of 1e-4 for the *PRK (PF00485.18)* domain from proteins Q9NWZ5 and P0A8F4. Note that Q9BZX2 and Q9HA47 only contain one of the domains, and were therefore not included in the discordant list.

###
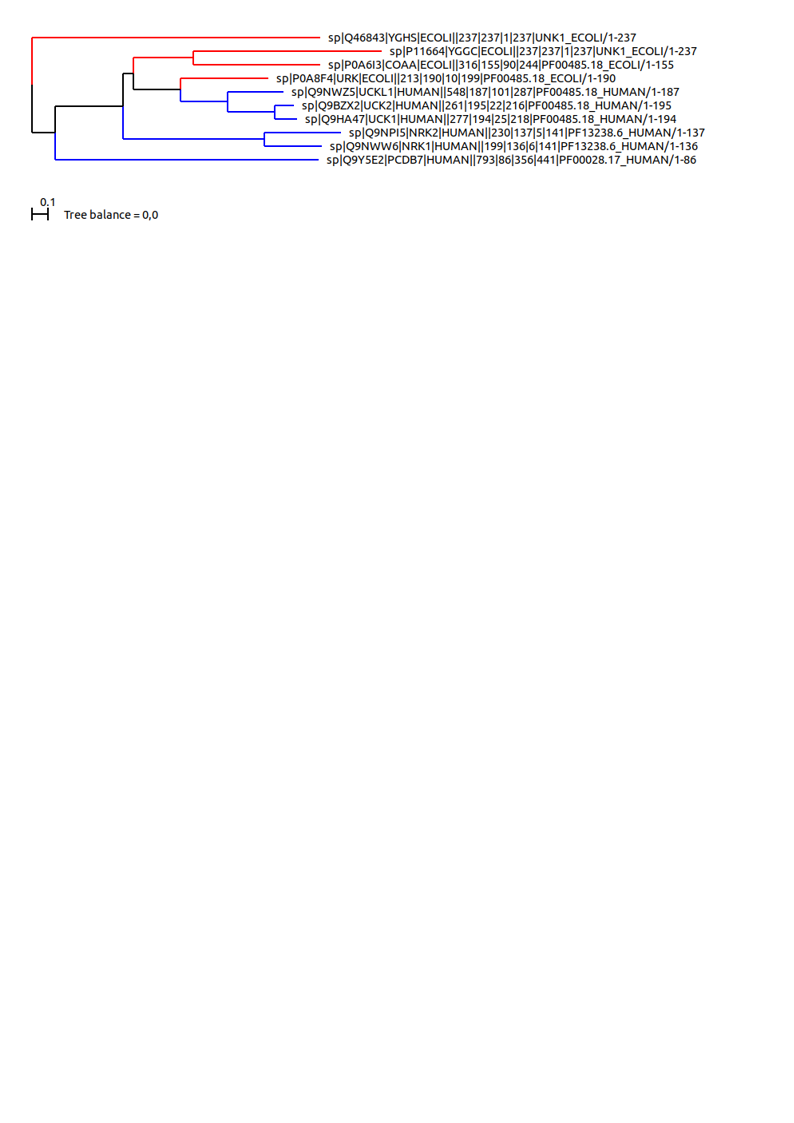


### Figure S2: NJ Tree of the closest homologs found when running BLAST with an e-value cutoff of 1e-4 for the *UPRTase (PF14681.6)* domain from proteins Q9NWZ5 and P0A8F0. Note that Q96BW1 only contains one of the domains, and was therefore not included in the discordant list.

###
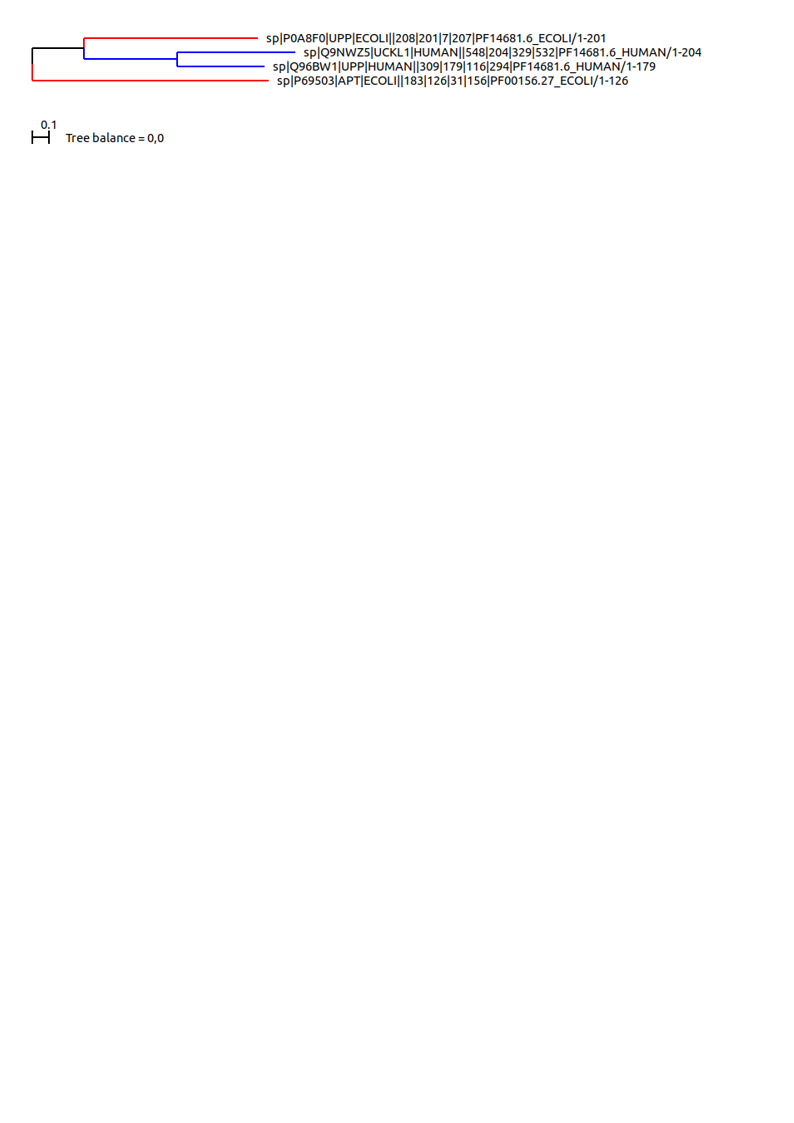


### Figure S3: NJ Tree of the homologs found when running BLAST with an e-value cutoff of 1e-4 for the *Carb_Kinase (PF01256.17)* domain from proteins P31806 and Q8IW45.
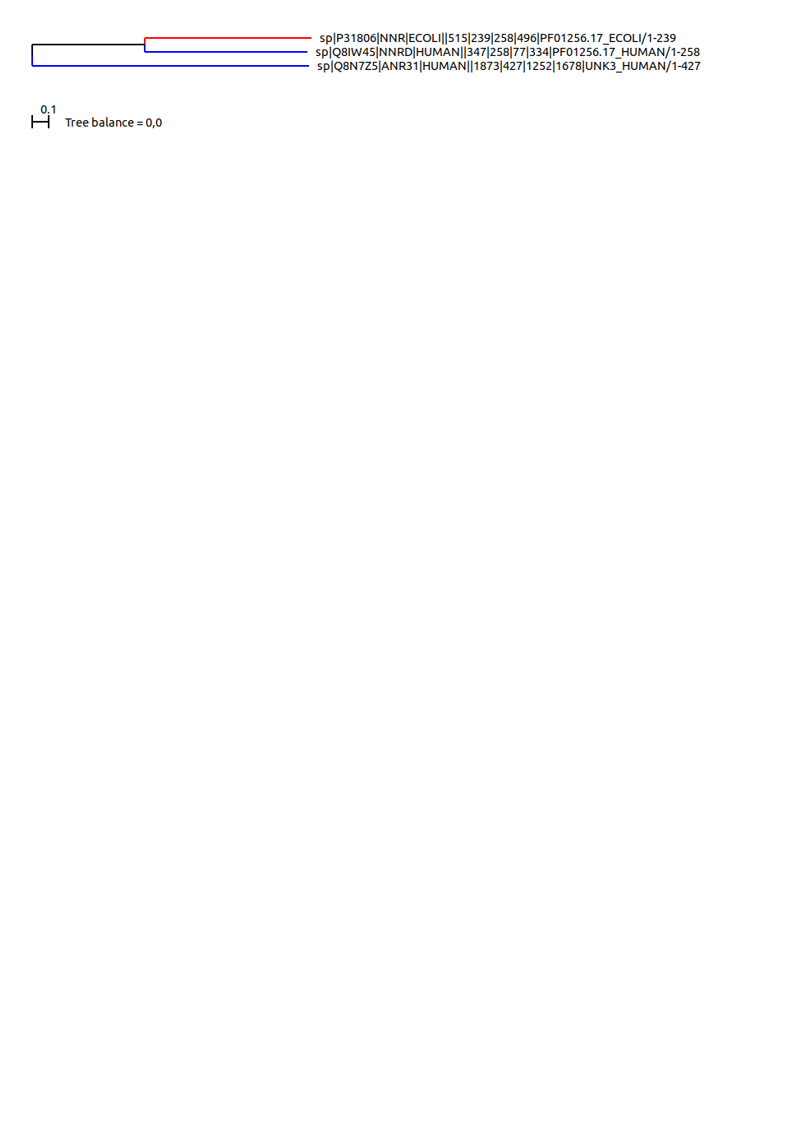


### Figure S4: NJ Tree of the homologs found when running BLAST with an e-value cutoff of 1e-4 for the *YjeF_N (PF03853.15)* domain from proteins P31806, A6XGL0, Q8NCW5 and E7ENQ6.


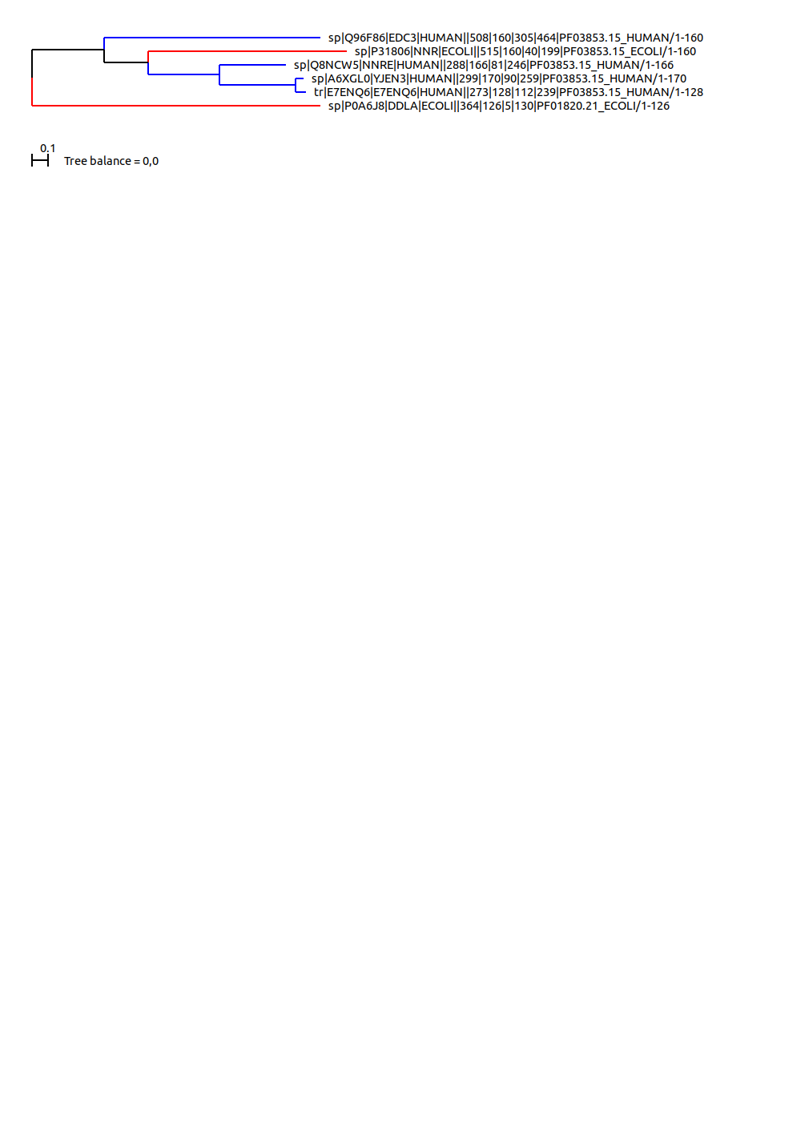


### Figure S5: NJ Tree of the homologs found when running BLAST with an e-value cutoff of 1e-5 for the *DnaJ (PF00226.31)* domain from the proteins Q9UBS3 and P08622.


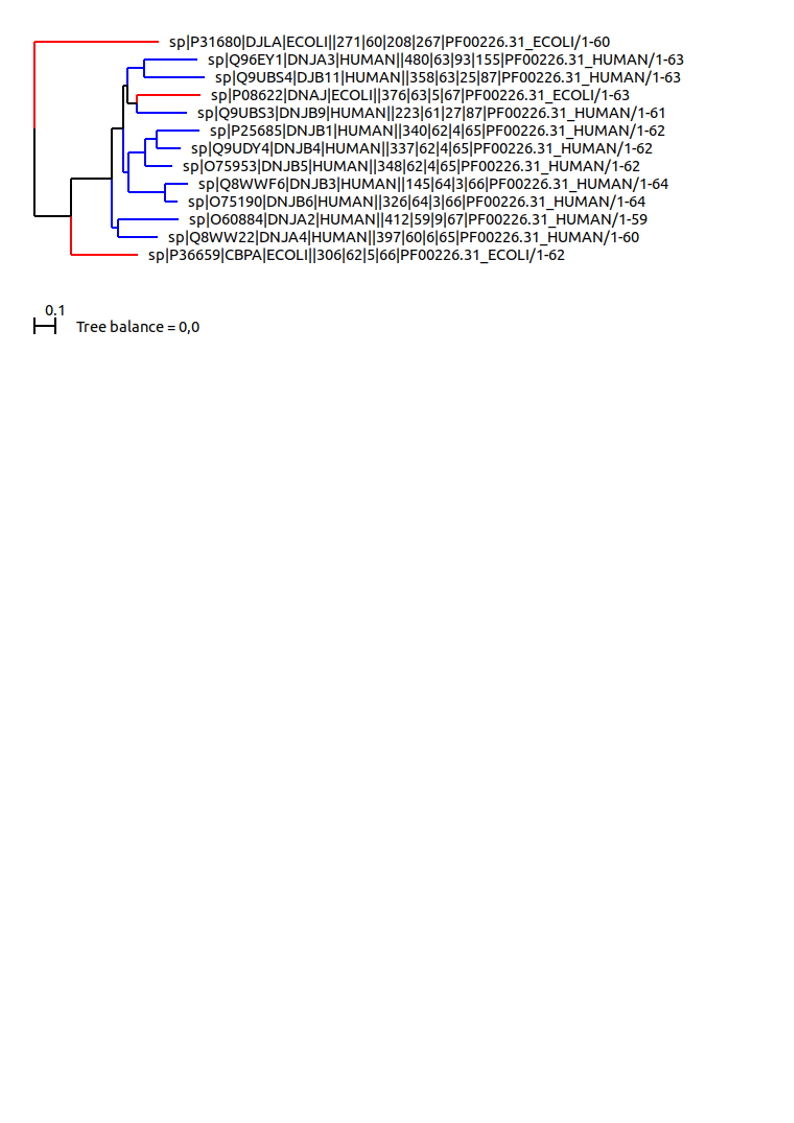


### Figure S6: NJ Tree of the homologs found when running BLAST with an e-value cutoff of 1e-4 for the *AIRS (PF00586.24)* domain from the proteins P22102 and P08178.


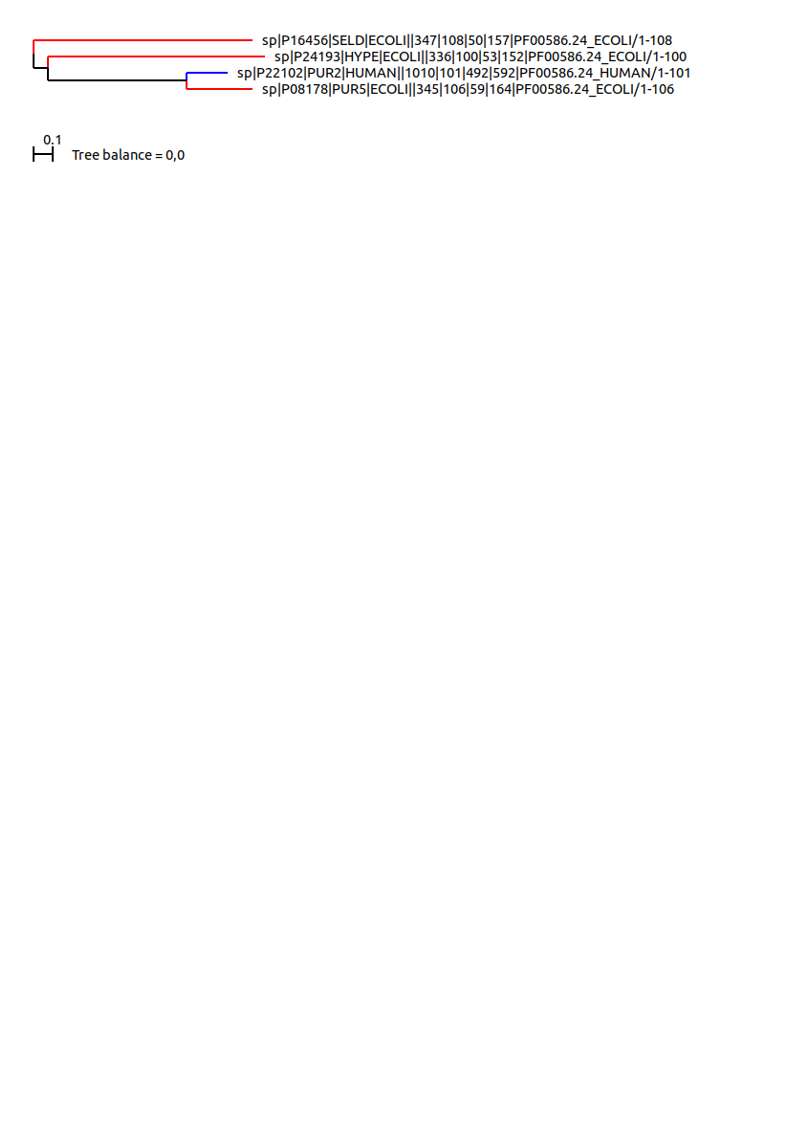


Figure S7: Distribution of ortholog pairs at different alpha-thresholds for the fungal subset of the QFO reference data in relation to InParanoid results.


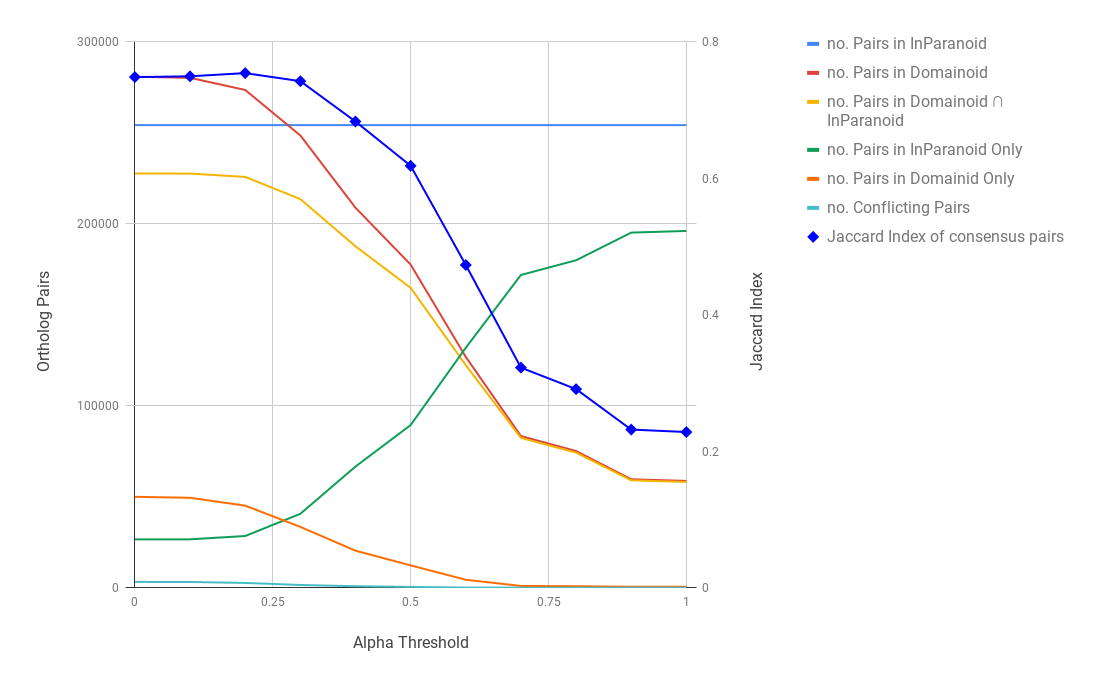


Figure S8. Average RF distance (vertical axis) and number of completed tree samplings (horizontal axis) from the Generalized species tree discordance test on the Orthology Benchmarking Service of InParanoid enriched with Domainoid at different alpha thresholds.


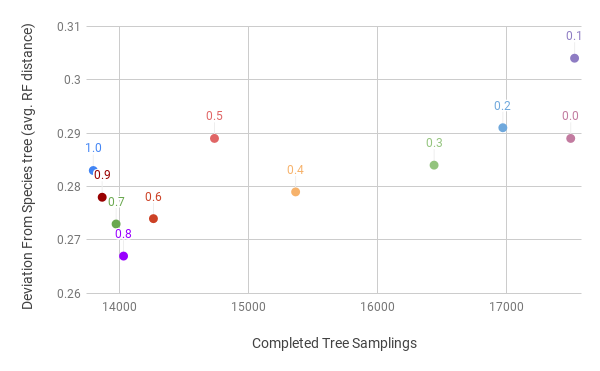

Supplement: Supplementary file 1 — Additional file 1: Figures S1–S6. containing trees to support the orthology for the examples, Figure S7. showing the distribution of orthologs from Domainoid in relation to InParanoid for fungi, and Figure S8. showing performance of Domainoid merged with InParanoid in the Generalized species tree discordance test for fungi on different alpha thresholds. [file 12859_2019_3137_MOESM1_ESM.docx]
