# Supplementary material for: Domainoid: domain-oriented orthology inference
Source: BMC Bioinformatics. 2019 Oct 28;20:523. doi: 10.1186/s12859-019-3137-2 (PMC6816169; doi:10.1186/s12859-019-3137-2)
Supplement: Supplementary file 2 — Additional file 2. Including a list of discordant domain orthologs for Homo sapiens/Escherichia coli. [file 12859_2019_3137_MOESM2_ESM.docx]

## Discordant domain orthologs for *Homo sapiens*/*Escherichia coli*

The discordant domain orthologs were generated using proteomes from QfO2018_04 (*Homo sapiens*, *Escherichia coli*), Pfam 32.0, Hmmer 3.1b2, InParanoid 4.1, pfam_scan.pl 2017-02-23, and BLAST 2.2.18.

Format:

*<Protein name and description>*

*<ortholog group>_<sequence length>|<domain length>|<domain start coordinate>|<domain stop coordinate>|<domain name>*

Primary: sp|P31806|NNR_ECOLI Bifunctional NAD(P)H-hydrate repair enzyme Nnr OS=Escherichia coli (strain K12) OX=83333 GN=nnr PE=1 SV=2

584_515|239|258|496|PF01256.17, 616_515|160|40|199|PF03853.15, 515|39|1|39|UNK1, 515|58|200|257|UNK2

Secondary: sp|A6XGL0|YJEN3_HUMAN YjeF N-terminal domain-containing protein 3 OS=Homo sapiens OX=9606 GN=YJEFN3 PE=1 SV=1

616_299|170|90|259|PF03853.15, 299|89|1|89|UNK1, 299|40|260|299|UNK2

Secondary: sp|Q8IW45|NNRD_HUMAN ATP-dependent (S)-NAD(P)H-hydrate dehydratase OS=Homo sapiens OX=9606 GN=NAXD PE=1 SV=1

584_347|258|77|334|PF01256.17, 347|76|1|76|UNK1

Secondary: sp|Q8NCW5|NNRE_HUMAN NAD(P)H-hydrate epimerase OS=Homo sapiens OX=9606 GN=NAXE PE=1 SV=2

616_288|166|81|246|PF03853.15, 288|80|1|80|UNK1, 288|42|247|288|UNK2

Secondary: tr|E7ENQ6|E7ENQ6_HUMAN Uncharacterized protein OS=Homo sapiens OX=9606 PE=4 SV=1

616_273|128|112|239|PF03853.15, 273|102|5|106|PF06212.12, 273|34|240|273|UNK1

Primary: sp|P60752|MSBA_ECOLI Lipid A export ATP-binding/permease protein MsbA OS=Escherichia coli (strain K12) OX=83333 GN=msbA PE=1 SV=1

213_582|151|359|509|PF00005.27, 468_582|73|510|582|UNK2, 582|271|27|297|PF00664.23, 582|61|298|358|UNK1

Secondary: sp|O95342|ABCBB_HUMAN Bile salt export pump OS=Homo sapiens OX=9606 GN=ABCB11 PE=1 SV=2

213_1321|149|438|586|PF00005.27, 213_1321|152|1096|1247|PF00005.27, 1321|308|63|370|PF00664.23, 1321|270|757|1026|PF00664.23, 1321|62|1|62|UNK1, 1321|67|371|437|UNK2, 1321|170|587|756|UNK3, 1321|69|1027|1095|UNK4, 1321|74|1248|1321|UNK5

Secondary: sp|P08183|MDR1_HUMAN Multidrug resistance protein 1 OS=Homo sapiens OX=9606 GN=ABCB1 PE=1 SV=3

213_1280|150|410|559|PF00005.27, 213_1280|152|1053|1204|PF00005.27, 1280|292|52|343|PF00664.23, 1280|275|712|986|PF00664.23, 1280|51|1|51|UNK1, 1280|66|344|409|UNK2, 1280|152|560|711|UNK3, 1280|66|987|1052|UNK4, 1280|76|1205|1280|UNK5

Secondary: sp|P21439|MDR3_HUMAN Phosphatidylcholine translocator ABCB4 OS=Homo sapiens OX=9606 GN=ABCB4 PE=1 SV=2

213_1286|150|412|561|PF00005.27, 213_1286|159|1052|1210|PF00005.27, 1286|288|58|345|PF00664.23, 1286|274|712|985|PF00664.23, 1286|57|1|57|UNK1, 1286|66|346|411|UNK2, 1286|150|562|711|UNK3, 1286|66|986|1051|UNK4, 1286|76|1211|1286|UNK5

Secondary: sp|Q2M3G0|ABCB5_HUMAN ATP-binding cassette sub-family B member 5 OS=Homo sapiens OX=9606 GN=ABCB5 PE=1 SV=4

213_1257|150|404|553|PF00005.27, 213_1257|152|1033|1184|PF00005.27, 1257|286|48|333|PF00664.23, 1257|273|693|965|PF00664.23, 1257|47|1|47|UNK1, 1257|70|334|403|UNK2, 1257|139|554|692|UNK3, 1257|67|966|1032|UNK4, 1257|73|1185|1257|UNK5

Secondary: sp|Q9NP58|ABCB6_HUMAN ATP-binding cassette sub-family B member 6, mitochondrial OS=Homo sapiens OX=9606 GN=ABCB6 PE=1 SV=1

250_842|150|606|755|PF00005.27, 468_842|87|756|842|UNK2, 842|250|6|255|PF16185.5, 842|271|274|544|PF00664.23, 842|61|545|605|UNK1

Secondary: sp|Q9NRK6|ABCBA_HUMAN ATP-binding cassette sub-family B member 10, mitochondrial OS=Homo sapiens OX=9606 GN=ABCB10 PE=1 SV=2

213_738|152|511|662|PF00005.27, 462_738|264|175|438|PF00664.23, 738|174|1|174|UNK1, 738|72|439|510|UNK2, 738|76|663|738|UNK3

Secondary: sp|Q9NUT2|ABCB8_HUMAN ATP-binding cassette sub-family B member 8, mitochondrial OS=Homo sapiens OX=9606 GN=ABCB8 PE=1 SV=3

213_735|151|490|640|PF00005.27, 462_735|276|149|424|PF00664.23, 735|148|1|148|UNK1, 735|65|425|489|UNK2, 735|95|641|735|UNK3

Primary: sp|Q9NQX3|GEPH_HUMAN Gephyrin OS=Homo sapiens OX=9606 GN=GPHN PE=1 SV=1

438_736|144|502|645|PF00994.24, 461_736|165|325|489|PF03453.17, 465_736|147|18|164|PF00994.24, 736|75|658|732|PF03454.15, 736|160|165|324|UNK1

Secondary: sp|P0AF03|MOG_ECOLI Molybdopterin adenylyltransferase OS=Escherichia coli (strain K12) OX=83333 GN=mog PE=1 SV=1

465_195|140|8|147|PF00994.24, 195|48|148|195|UNK1

Secondary: sp|P12281|MOEA_ECOLI Molybdopterin molybdenumtransferase OS=Escherichia coli (strain K12) OX=83333 GN=moeA PE=1 SV=1

438_411|136|182|317|PF00994.24, 461_411|160|9|168|PF03453.17, 411|73|332|404|PF03454.15

Primary: sp|Q9NUQ8|ABCF3_HUMAN ATP-binding cassette sub-family F member 3 OS=Homo sapiens OX=9606 GN=ABCF3 PE=1 SV=2

364_709|161|196|356|PF00005.27, 377_709|130|510|639|PF00005.27, 612_709|81|395|475|PF12848.7, 758_709|70|640|709|UNK4, 855_709|38|357|394|UNK2, 709|195|1|195|UNK1, 709|34|476|509|UNK3

Secondary: sp|P0A9W3|ETTA_ECOLI Energy-dependent translational throttle protein EttA OS=Escherichia coli (strain K12) OX=83333 GN=ettA PE=1 SV=2

855_555|38|192|229|UNK1, 555|170|22|191|PF00005.27, 555|77|230|306|PF12848.7, 555|134|340|473|PF00005.27, 555|33|307|339|UNK2, 555|82|474|555|UNK3

Secondary: sp|P43672|UUP_ECOLI ABC transporter ATP-binding protein uup OS=Escherichia coli (strain K12) OX=83333 GN=uup PE=1 SV=2

758_635|91|470|560|UNK3, 855_635|38|186|223|UNK1, 635|167|19|185|PF00005.27, 635|70|224|293|PF12848.7, 635|134|336|469|PF00005.27, 635|69|561|629|PF16326.5, 635|42|294|335|UNK2

Secondary: sp|P63389|YHES_ECOLI Uncharacterized ABC transporter ATP-binding protein YheS OS=Escherichia coli (strain K12) OX=83333 GN=yheS PE=1 SV=1

364_637|162|17|178|PF00005.27, 377_637|132|328|459|PF00005.27, 612_637|82|217|298|PF12848.7, 814_637|38|179|216|UNK1, 637|178|460|637|UNK2

Primary: sp|P54886|P5CS_HUMAN Delta-1-pyrroline-5-carboxylate synthase OS=Homo sapiens OX=9606 GN=ALDH18A1 PE=1 SV=2

216_795|264|364|627|PF00171.22, 322_795|259|71|329|PF00696.28, 339_795|168|628|795|UNK3, 795|70|1|70|UNK1, 795|34|330|363|UNK2

Secondary: sp|P07004|PROA_ECOLI Gamma-glutamyl phosphate reductase OS=Escherichia coli (strain K12) OX=83333 GN=proA PE=1 SV=2

216_417|275|8|282|PF00171.22, 339_417|135|283|417|UNK1

Secondary: sp|P0A7B5|PROB_ECOLI Glutamate 5-kinase OS=Escherichia coli (strain K12) OX=83333 GN=proB PE=1 SV=1

322_367|229|6|234|PF00696.28, 367|75|276|350|PF01472.20, 367|41|235|275|UNK1

Primary: sp|Q8N3C0|ASCC3_HUMAN Activating signal cointegrator 1 complex subunit 3 OS=Homo sapiens OX=9606 GN=ASCC3 PE=1 SV=3

700_2202|166|1329|1494|PF00270.29, 700_2202|175|479|653|PF00270.29, 789_2202|152|704|855|PF00271.31, 2202|306|978|1283|PF02889.16, 2202|86|1607|1692|PF00271.31, 2202|364|1812|2175|PF02889.16, 2202|478|1|478|UNK1, 2202|50|654|703|UNK2, 2202|122|856|977|UNK3, 2202|45|1284|1328|UNK4, 2202|112|1495|1606|UNK5, 2202|119|1693|1811|UNK6

Secondary: sp|P24230|RECG_ECOLI ATP-dependent DNA helicase RecG OS=Escherichia coli (strain K12) OX=83333 GN=recG PE=1 SV=1

789_693|108|481|588|PF00271.31, 693|157|15|171|PF17191.4, 693|161|273|433|PF00270.29, 693|101|172|272|UNK1, 693|47|434|480|UNK2, 693|105|589|693|UNK3

Secondary: sp|P30015|LHR_ECOLI Probable ATP-dependent helicase lhr OS=Escherichia coli (strain K12) OX=83333 GN=lhr PE=3 SV=2

700_1538|188|31|218|PF00270.29, 1538|69|349|417|PF00271.31, 1538|189|679|867|PF08494.11, 1538|30|1|30|UNK1, 1538|130|219|348|UNK2, 1538|261|418|678|UNK3, 1538|671|868|1538|UNK4

Secondary: sp|P30958|MFD_ECOLI Transcription-repair-coupling factor OS=Escherichia coli (strain K12) OX=83333 GN=mfd PE=1 SV=2

789_1148|104|803|906|PF00271.31, 1148|89|129|217|PF17757.1, 1148|96|477|572|PF02559.16, 1148|161|602|762|PF00270.29, 1148|93|1006|1098|PF03461.15, 1148|128|1|128|UNK1, 1148|259|218|476|UNK2, 1148|40|763|802|UNK3, 1148|99|907|1005|UNK4, 1148|50|1099|1148|UNK5

Primary: sp|P43329|HRPA_ECOLI ATP-dependent RNA helicase HrpA OS=Escherichia coli (strain K12) OX=83333 GN=hrpA PE=3 SV=3

194_1300|149|85|233|PF00270.29, 381_1300|119|285|403|PF00271.31, 687_1300|86|466|551|PF04408.23, 719_1300|62|404|465|UNK3, 862_1300|77|622|698|PF07717.16, 1300|585|713|1297|PF11898.8, 1300|84|1|84|UNK1, 1300|51|234|284|UNK2, 1300|70|552|621|UNK4

Secondary: sp|O43143|DHX15_HUMAN Pre-mRNA-splicing factor ATP-dependent RNA helicase DHX15 OS=Homo sapiens OX=9606 GN=DHX15 PE=1 SV=2

194_795|148|149|296|PF00270.29, 381_795|139|338|476|PF00271.31, 687_795|89|539|627|PF04408.23, 862_795|77|689|765|PF07717.16, 795|148|1|148|UNK1, 795|41|297|337|UNK2, 795|62|477|538|UNK3, 795|61|628|688|UNK4, 795|30|766|795|UNK5

Secondary: sp|O60231|DHX16_HUMAN Pre-mRNA-splicing factor ATP-dependent RNA helicase DHX16 OS=Homo sapiens OX=9606 GN=DHX16 PE=1 SV=2

194_1041|152|405|556|PF00270.29, 381_1041|127|603|729|PF00271.31, 687_1041|74|792|865|PF04408.23, 862_1041|77|939|1015|PF07717.16, 1041|404|1|404|UNK1, 1041|46|557|602|UNK2, 1041|62|730|791|UNK3, 1041|73|866|938|UNK4

Secondary: sp|Q14562|DHX8_HUMAN ATP-dependent RNA helicase DHX8 OS=Homo sapiens OX=9606 GN=DHX8 PE=1 SV=1

381_1220|129|767|895|PF00271.31, 687_1220|89|957|1045|PF04408.23, 755_1220|74|261|334|PF00575.23, 862_1220|77|1103|1179|PF07717.16, 1220|260|1|260|UNK1, 1220|432|335|766|UNK2, 1220|61|896|956|UNK3, 1220|57|1046|1102|UNK4, 1220|41|1180|1220|UNK5

Secondary: sp|Q7L7V1|DHX32_HUMAN Putative pre-mRNA-splicing factor ATP-dependent RNA helicase DHX32 OS=Homo sapiens OX=9606 GN=DHX32 PE=1 SV=1

862_743|81|616|696|PF07717.16, 743|90|458|547|PF04408.23, 743|457|1|457|UNK1, 743|68|548|615|UNK2, 743|47|697|743|UNK3

Secondary: sp|Q8IX18|DHX40_HUMAN Probable ATP-dependent RNA helicase DHX40 OS=Homo sapiens OX=9606 GN=DHX40 PE=1 SV=2

194_779|157|60|216|PF00270.29, 687_779|78|463|540|PF04408.23, 862_779|80|619|698|PF07717.16, 779|137|264|400|PF00271.31, 779|59|1|59|UNK1, 779|47|217|263|UNK2, 779|62|401|462|UNK3, 779|78|541|618|UNK4, 779|81|699|779|UNK5

Secondary: sp|Q8IY37|DHX37_HUMAN Probable ATP-dependent RNA helicase DHX37 OS=Homo sapiens OX=9606 GN=DHX37 PE=1 SV=1

862_1157|86|925|1010|PF07717.16, 1157|155|261|415|PF00270.29, 1157|87|589|675|PF00271.31, 1157|122|737|858|PF04408.23, 1157|260|1|260|UNK1, 1157|173|416|588|UNK2, 1157|61|676|736|UNK3, 1157|66|859|924|UNK4, 1157|147|1011|1157|UNK5

Secondary: sp|Q92620|PRP16_HUMAN Pre-mRNA-splicing factor ATP-dependent RNA helicase PRP16 OS=Homo sapiens OX=9606 GN=DHX38 PE=1 SV=2

194_1227|153|536|688|PF00270.29, 381_1227|127|735|861|PF00271.31, 687_1227|86|925|1010|PF04408.23, 862_1227|78|1069|1146|PF07717.16, 1227|535|1|535|UNK1, 1227|46|689|734|UNK2, 1227|63|862|924|UNK3, 1227|58|1011|1068|UNK4, 1227|81|1147|1227|UNK5

Secondary: sp|Q9H5Z1|DHX35_HUMAN Probable ATP-dependent RNA helicase DHX35 OS=Homo sapiens OX=9606 GN=DHX35 PE=1 SV=2

381_703|133|265|397|PF00271.31, 687_703|90|459|548|PF04408.23, 719_703|61|398|458|UNK2, 862_703|76|608|683|PF07717.16, 703|264|1|264|UNK1, 703|59|549|607|UNK3

Secondary: sp|Q9H6R0|DHX33_HUMAN ATP-dependent RNA helicase DHX33 OS=Homo sapiens OX=9606 GN=DHX33 PE=1 SV=2

687_707|92|471|562|PF04408.23, 719_707|62|409|470|UNK3, 862_707|77|620|696|PF07717.16, 707|146|90|235|PF00270.29, 707|126|283|408|PF00271.31, 707|89|1|89|UNK1, 707|47|236|282|UNK2, 707|57|563|619|UNK4

Primary: sp|P0ABQ2|GARR_ECOLI 2-hydroxy-3-oxopropionate reductase OS=Escherichia coli (strain K12) OX=83333 GN=garR PE=1 SV=1

282_294|160|2|161|PF03446.15, 813_294|118|164|281|PF14833.6

Secondary: sp|P31937|3HIDH_HUMAN 3-hydroxyisobutyrate dehydrogenase, mitochondrial OS=Homo sapiens OX=9606 GN=HIBADH PE=1 SV=2

282_336|159|42|200|PF03446.15, 704_336|128|203|330|PF14833.6, 336|41|1|41|UNK1

Secondary: sp|Q49A26|GLYR1_HUMAN Putative oxidoreductase GLYR1 OS=Homo sapiens OX=9606 GN=GLYR1 PE=1 SV=3

813_553|121|431|551|PF14833.6, 553|82|7|88|PF00855.17, 553|157|269|425|PF03446.15, 553|180|89|268|UNK1

Primary: sp|Q12882|DPYD_HUMAN Dihydropyrimidine dehydrogenase [NADP(+)] OS=Homo sapiens OX=9606 GN=DPYD PE=1 SV=2

150_1025|305|534|838|PF01180.21, 343_1025|303|190|492|PF07992.14, 450_1025|110|57|166|PF14691.6, 683_1025|59|946|1004|PF14697.6, 1025|56|1|56|UNK1, 1025|41|493|533|UNK2, 1025|107|839|945|UNK3

Secondary: sp|P09832|GLTD_ECOLI Glutamate synthase [NADPH] small chain OS=Escherichia coli (strain K12) OX=83333 GN=gltD PE=1 SV=3

343_472|313|147|459|PF07992.14, 472|110|25|134|PF14691.6

Secondary: sp|P25889|PREA_ECOLI NAD-dependent dihydropyrimidine dehydrogenase subunit PreA OS=Escherichia coli (strain K12) OX=83333 GN=preA PE=1 SV=3

150_411|299|6|304|PF01180.21, 683_411|57|338|394|PF14697.6, 411|33|305|337|UNK1

Secondary: sp|P37127|AEGA_ECOLI Protein AegA OS=Escherichia coli (strain K12) OX=83333 GN=aegA PE=3 SV=2

343_659|313|328|640|PF07992.14, 659|90|55|144|PF13247.6, 659|111|205|315|PF14691.6, 659|54|1|54|UNK1, 659|60|145|204|UNK2

Secondary: sp|P76440|PRET_ECOLI NAD-dependent dihydropyrimidine dehydrogenase subunit PreT OS=Escherichia coli (strain K12) OX=83333 GN=preT PE=1 SV=1

450_412|107|7|113|PF14691.6, 412|269|126|394|PF07992.14

Secondary: sp|Q46820|YGFT_ECOLI Uncharacterized protein YgfT OS=Escherichia coli (strain K12) OX=83333 GN=ygfT PE=3 SV=2

343_639|312|312|623|PF07992.14, 639|85|53|137|PF13247.6, 639|112|187|298|PF14691.6, 639|52|1|52|UNK1, 639|49|138|186|UNK2

Primary: sp|P23909|MUTS_ECOLI DNA mismatch repair protein MutS OS=Escherichia coli (strain K12) OX=83333 GN=mutS PE=1 SV=1

127_853|188|610|797|PF00488.21, 630_853|288|272|559|PF05192.18, 819_853|112|11|122|PF01624.20, 853|126|131|256|PF05188.17, 853|50|560|609|UNK1, 853|56|798|853|UNK2

Secondary: sp|P43246|MSH2_HUMAN DNA mismatch repair protein Msh2 OS=Homo sapiens OX=9606 GN=MSH2 PE=1 SV=1

127_934|187|666|852|PF00488.21, 630_934|304|306|609|PF05192.18, 934|114|18|131|PF01624.20, 934|134|156|289|PF05188.17, 934|56|610|665|UNK1, 934|82|853|934|UNK2

Secondary: sp|P52701|MSH6_HUMAN DNA mismatch repair protein Msh6 OS=Homo sapiens OX=9606 GN=MSH6 PE=1 SV=2

819_1360|118|407|524|PF01624.20, 1360|94|90|183|PF00855.17, 1360|162|538|699|PF05188.17, 1360|326|739|1064|PF05192.18, 1360|193|1131|1323|PF00488.21, 1360|89|1|89|UNK1, 1360|223|184|406|UNK2, 1360|39|700|738|UNK3, 1360|66|1065|1130|UNK4, 1360|37|1324|1360|UNK5

Primary: sp|Q9NP58|ABCB6_HUMAN ATP-binding cassette sub-family B member 6, mitochondrial OS=Homo sapiens OX=9606 GN=ABCB6 PE=1 SV=1

250_842|150|606|755|PF00005.27, 468_842|87|756|842|UNK2, 842|250|6|255|PF16185.5, 842|271|274|544|PF00664.23, 842|61|545|605|UNK1

Secondary: sp|P60752|MSBA_ECOLI Lipid A export ATP-binding/permease protein MsbA OS=Escherichia coli (strain K12) OX=83333 GN=msbA PE=1 SV=1

213_582|151|359|509|PF00005.27, 468_582|73|510|582|UNK2, 582|271|27|297|PF00664.23, 582|61|298|358|UNK1

Secondary: sp|P77265|MDLA_ECOLI Multidrug resistance-like ATP-binding protein MdlA OS=Escherichia coli (strain K12) OX=83333 GN=mdlA PE=3 SV=1

250_590|149|352|500|PF00005.27, 590|270|22|291|PF00664.23, 590|60|292|351|UNK1, 590|90|501|590|UNK2

Primary: sp|P75757|ZITB_ECOLI Zinc transporter ZitB OS=Escherichia coli (strain K12) OX=83333 GN=zitB PE=1 SV=1

274_313|192|22|213|PF01545.21, 880_313|100|214|313|UNK1

Secondary: sp|O14863|ZNT4_HUMAN Zinc transporter 4 OS=Homo sapiens OX=9606 GN=SLC30A4 PE=1 SV=2

880_429|97|333|429|UNK2, 429|219|114|332|PF01545.21, 429|113|1|113|UNK1

Secondary: sp|Q8IWU4|ZNT8_HUMAN Zinc transporter 8 OS=Homo sapiens OX=9606 GN=SLC30A8 PE=1 SV=2

880_369|98|272|369|UNK2, 369|195|77|271|PF01545.21, 369|76|1|76|UNK1

Secondary: sp|Q99726|ZNT3_HUMAN Zinc transporter 3 OS=Homo sapiens OX=9606 GN=SLC30A3 PE=1 SV=2

880_388|95|294|388|UNK2, 388|218|76|293|PF01545.21, 388|75|1|75|UNK1

Secondary: sp|Q9BRI3|ZNT2_HUMAN Zinc transporter 2 OS=Homo sapiens OX=9606 GN=SLC30A2 PE=1 SV=1

880_323|94|230|323|UNK2, 323|140|90|229|PF01545.21, 323|89|1|89|UNK1

Secondary: sp|Q9Y6M5|ZNT1_HUMAN Zinc transporter 1 OS=Homo sapiens OX=9606 GN=SLC30A1 PE=1 SV=3

274_507|261|12|272|PF01545.21, 507|235|273|507|UNK1

Primary: sp|Q8N139|ABCA6_HUMAN ATP-binding cassette sub-family A member 6 OS=Homo sapiens OX=9606 GN=ABCA6 PE=1 SV=2

400_1617|137|1304|1440|PF00005.27, 443_1617|146|497|642|PF00005.27, 841_1617|177|1441|1617|UNK5, 888_1617|224|643|866|UNK3, 1617|384|33|416|PF12698.7, 1617|301|867|1167|PF12698.7, 1617|32|1|32|UNK1, 1617|80|417|496|UNK2, 1617|136|1168|1303|UNK4

Secondary: sp|P0A9U1|YBHF_ECOLI Probable multidrug ABC transporter ATP-binding protein YbhF OS=Escherichia coli (strain K12) OX=83333 GN=ybhF PE=1 SV=1

400_578|144|346|489|PF00005.27, 443_578|140|26|165|PF00005.27, 888_578|89|490|578|UNK2, 578|180|166|345|UNK1

Secondary: sp|P36879|YADG_ECOLI Uncharacterized ABC transporter ATP-binding protein YadG OS=Escherichia coli (strain K12) OX=83333 GN=yadG PE=1 SV=1

417_308|145|21|165|PF00005.27, 841_308|143|166|308|UNK1

Secondary: sp|P37624|RBBA_ECOLI Ribosome-associated ATPase OS=Escherichia coli (strain K12) OX=83333 GN=rbbA PE=1 SV=3

400_911|144|293|436|PF00005.27, 443_911|148|28|175|PF00005.27, 888_911|132|437|568|UNK2, 888_911|117|176|292|UNK1, 911|335|569|903|PF12698.7

Primary: sp|Q04656|ATP7A_HUMAN Copper-transporting ATPase 1 OS=Homo sapiens OX=9606 GN=ATP7A PE=1 SV=3

239_1500|206|816|1021|PF00122.20, 288_1500|276|1038|1313|PF00702.26, 691_1500|187|629|815|UNK5, 746_1500|187|1314|1500|UNK6, 827_1500|62|380|441|PF00403.26, 827_1500|62|11|72|PF00403.26, 827_1500|59|176|234|PF00403.26, 827_1500|59|570|628|PF00403.26, 827_1500|59|493|551|PF00403.26, 827_1500|58|280|337|PF00403.26, 1500|103|73|175|UNK1, 1500|45|235|279|UNK2, 1500|42|338|379|UNK3, 1500|51|442|492|UNK4

Secondary: sp|P37617|ZNTA_ECOLI Zinc/cadmium/lead-transporting P-type ATPase OS=Escherichia coli (strain K12) OX=83333 GN=zntA PE=1 SV=1

288_732|207|433|639|PF00702.26, 746_732|93|640|732|UNK3, 732|57|53|109|PF00403.26, 732|181|234|414|PF00122.20, 732|52|1|52|UNK1, 732|124|110|233|UNK2

Secondary: sp|Q59385|COPA_ECOLI Copper-exporting P-type ATPase OS=Escherichia coli (strain K12) OX=83333 GN=copA PE=1 SV=4

239_834|181|320|500|PF00122.20, 288_834|215|518|732|PF00702.26, 497_834|102|733|834|UNK3, 691_834|160|160|319|UNK2, 827_834|55|105|159|PF00403.26, 834|55|8|62|PF00403.26, 834|42|63|104|UNK1

Primary: sp|P21693|DBPA_ECOLI ATP-dependent RNA helicase DbpA OS=Escherichia coli (strain K12) OX=83333 GN=dbpA PE=1 SV=2

218_457|165|27|191|PF00270.29, 434_457|107|230|336|PF00271.31, 457|71|384|454|PF03880.15, 457|38|192|229|UNK1, 457|47|337|383|UNK2

Secondary: sp|Q9BQ39|DDX50_HUMAN ATP-dependent RNA helicase DDX50 OS=Homo sapiens OX=9606 GN=DDX50 PE=1 SV=1

218_737|175|161|335|PF00270.29, 737|97|387|483|PF00271.31, 737|95|571|665|PF08152.12, 737|160|1|160|UNK1, 737|51|336|386|UNK2, 737|87|484|570|UNK3, 737|72|666|737|UNK4

Secondary: sp|Q9H0S4|DDX47_HUMAN Probable ATP-dependent RNA helicase DDX47 OS=Homo sapiens OX=9606 GN=DDX47 PE=1 SV=1

434_455|108|251|358|PF00271.31, 455|168|48|215|PF00270.29, 455|47|1|47|UNK1, 455|35|216|250|UNK2, 455|97|359|455|UNK3

Secondary: sp|Q9NR30|DDX21_HUMAN Nucleolar RNA helicase 2 OS=Homo sapiens OX=9606 GN=DDX21 PE=1 SV=5

218_783|171|210|380|PF00270.29, 783|97|436|532|PF00271.31, 783|96|620|715|PF08152.12, 783|209|1|209|UNK1, 783|55|381|435|UNK2, 783|87|533|619|UNK3, 783|68|716|783|UNK4

Secondary: sp|Q9Y6V7|DDX49_HUMAN Probable ATP-dependent RNA helicase DDX49 OS=Homo sapiens OX=9606 GN=DDX49 PE=1 SV=1

434_483|110|234|343|PF00271.31, 483|170|26|195|PF00270.29, 483|38|196|233|UNK1, 483|140|344|483|UNK2

Primary: sp|P55809|SCOT1_HUMAN Succinyl-CoA:3-ketoacid coenzyme A transferase 1, mitochondrial OS=Homo sapiens OX=9606 GN=OXCT1 PE=1 SV=1

149_520|198|303|500|PF01144.23, 183_520|226|46|271|PF01144.23, 520|45|1|45|UNK1, 520|31|272|302|UNK2

Secondary: sp|P76458|ATOD_ECOLI Acetate CoA-transferase subunit alpha OS=Escherichia coli (strain K12) OX=83333 GN=atoD PE=1 SV=1

183_220|210|6|215|PF01144.23

Secondary: sp|P76459|ATOA_ECOLI Acetate CoA-transferase subunit beta OS=Escherichia coli (strain K12) OX=83333 GN=atoA PE=1 SV=1

149_216|175|5|179|PF01144.23, 216|37|180|216|UNK1

Primary: sp|P24555|PTRB_ECOLI Protease 2 OS=Escherichia coli (strain K12) OX=83333 GN=ptrB PE=1 SV=2

244_686|214|463|676|PF00326.21, 768_686|401|3|403|PF02897.15, 686|59|404|462|UNK1

Secondary: sp|P48147|PPCE_HUMAN Prolyl endopeptidase OS=Homo sapiens OX=9606 GN=PREP PE=1 SV=2

768_710|416|7|422|PF02897.15, 710|223|483|705|PF00326.21, 710|60|423|482|UNK1

Secondary: sp|Q4J6C6|PPCEL_HUMAN Prolyl endopeptidase-like OS=Homo sapiens OX=9606 GN=PREPL PE=1 SV=1

244_727|218|490|707|PF00326.21, 727|316|111|426|PF02897.15, 727|110|1|110|UNK1, 727|63|427|489|UNK2

Primary: sp|P37624|RBBA_ECOLI Ribosome-associated ATPase OS=Escherichia coli (strain K12) OX=83333 GN=rbbA PE=1 SV=3

400_911|144|293|436|PF00005.27, 443_911|148|28|175|PF00005.27, 888_911|132|437|568|UNK2, 888_911|117|176|292|UNK1, 911|335|569|903|PF12698.7

Secondary: sp|O94911|ABCA8_HUMAN ATP-binding cassette sub-family A member 8 OS=Homo sapiens OX=9606 GN=ABCA8 PE=1 SV=3

400_1581|139|1267|1405|PF00005.27, 841_1581|176|1406|1581|UNK5, 1581|381|38|418|PF12698.7, 1581|121|499|619|PF00005.27, 1581|240|901|1140|PF12698.7, 1581|37|1|37|UNK1, 1581|80|419|498|UNK2, 1581|281|620|900|UNK3, 1581|126|1141|1266|UNK4

Secondary: sp|O95477|ABCA1_HUMAN ATP-binding cassette sub-family A member 1 OS=Homo sapiens OX=9606 GN=ABCA1 PE=1 SV=3

443_2261|145|917|1061|PF00005.27, 841_2261|189|2073|2261|UNK5, 888_2261|285|1062|1346|UNK3, 2261|202|640|841|PF12698.7, 2261|522|1347|1868|PF12698.7, 2261|143|1930|2072|PF00005.27, 2261|639|1|639|UNK1, 2261|75|842|916|UNK2, 2261|61|1869|1929|UNK4

Secondary: sp|P78363|ABCA4_HUMAN Retinal-specific ATP-binding cassette transporter OS=Homo sapiens OX=9606 GN=ABCA4 PE=1 SV=3

443_2273|144|947|1090|PF00005.27, 443_2273|142|1957|2098|PF00005.27, 841_2273|175|2099|2273|UNK5, 2273|247|610|856|PF12698.7, 2273|292|1604|1895|PF12698.7, 2273|609|1|609|UNK1, 2273|90|857|946|UNK2, 2273|513|1091|1603|UNK3, 2273|61|1896|1956|UNK4

Secondary: sp|Q86UK0|ABCAC_HUMAN ATP-binding cassette sub-family A member 12 OS=Homo sapiens OX=9606 GN=ABCA12 PE=1 SV=3

443_2595|146|1361|1506|PF00005.27, 841_2595|178|2418|2595|UNK5, 888_2595|237|1507|1743|UNK3, 2595|337|934|1270|PF12698.7, 2595|424|1744|2167|PF12698.7, 2595|145|2273|2417|PF00005.27, 2595|933|1|933|UNK1, 2595|90|1271|1360|UNK2, 2595|105|2168|2272|UNK4

Secondary: sp|Q86UQ4|ABCAD_HUMAN ATP-binding cassette sub-family A member 13 OS=Homo sapiens OX=9606 GN=ABCA13 PE=2 SV=3

443_5058|146|3859|4004|PF00005.27, 841_5058|174|4885|5058|UNK5, 5058|224|3547|3770|PF12698.7, 5058|265|4390|4654|PF12698.7, 5058|147|4738|4884|PF00005.27, 5058|3546|1|3546|UNK1, 5058|88|3771|3858|UNK2, 5058|385|4005|4389|UNK3, 5058|83|4655|4737|UNK4

Secondary: sp|Q8IZY2|ABCA7_HUMAN ATP-binding cassette sub-family A member 7 OS=Homo sapiens OX=9606 GN=ABCA7 PE=1 SV=3

443_2146|145|824|968|PF00005.27, 888_2146|193|1954|2146|UNK5, 2146|200|551|750|PF12698.7, 2146|284|1466|1749|PF12698.7, 2146|142|1812|1953|PF00005.27, 2146|550|1|550|UNK1, 2146|73|751|823|UNK2, 2146|497|969|1465|UNK3, 2146|62|1750|1811|UNK4

Secondary: sp|Q8WWZ4|ABCAA_HUMAN ATP-binding cassette sub-family A member 10 OS=Homo sapiens OX=9606 GN=ABCA10 PE=2 SV=3

400_1543|145|1223|1367|PF00005.27, 443_1543|147|410|556|PF00005.27, 841_1543|176|1368|1543|UNK5, 1543|240|90|329|PF12698.7, 1543|195|936|1130|PF12698.7, 1543|89|1|89|UNK1, 1543|80|330|409|UNK2, 1543|379|557|935|UNK3, 1543|92|1131|1222|UNK4

Secondary: sp|Q99758|ABCA3_HUMAN ATP-binding cassette sub-family A member 3 OS=Homo sapiens OX=9606 GN=ABCA3 PE=1 SV=2

417_1704|144|550|693|PF00005.27, 443_1704|143|1400|1542|PF00005.27, 841_1704|162|1543|1704|UNK5, 888_1704|229|694|922|UNK3, 1704|218|252|469|PF12698.7, 1704|400|923|1322|PF12698.7, 1704|251|1|251|UNK1, 1704|80|470|549|UNK2, 1704|77|1323|1399|UNK4

Secondary: sp|Q9BZC7|ABCA2_HUMAN ATP-binding cassette sub-family A member 2 OS=Homo sapiens OX=9606 GN=ABCA2 PE=1 SV=3

443_2435|145|1007|1151|PF00005.27, 443_2435|142|2072|2213|PF00005.27, 841_2435|222|2214|2435|UNK5, 2435|208|705|912|PF12698.7, 2435|283|1725|2007|PF12698.7, 2435|704|1|704|UNK1, 2435|94|913|1006|UNK2, 2435|573|1152|1724|UNK3, 2435|64|2008|2071|UNK4

Primary: sp|P31327|CPSM_HUMAN Carbamoyl-phosphate synthase [ammonia], mitochondrial OS=Homo sapiens OX=9606 GN=CPS1 PE=1 SV=2

114_1500|204|546|749|PF02786.17, 187_1500|203|1088|1290|PF02786.17, 233_1500|174|222|395|PF00117.28, 302_1500|125|963|1087|UNK5, 313_1500|150|396|545|UNK3, 369_1500|138|46|183|PF00988.22, 453_1500|122|841|962|PF02787.19, 458_1500|91|750|840|UNK4, 866_1500|84|1291|1374|UNK6, 1500|91|1375|1465|PF02142.22, 1500|14|1483|1496|PF18302.1, 1500|45|1|45|UNK1, 1500|38|184|221|UNK2

Secondary: sp|P00968|CARB_ECOLI Carbamoyl-phosphate synthase large chain OS=Escherichia coli (strain K12) OX=83333 GN=carB PE=1 SV=2

114_1073|207|128|334|PF02786.17, 187_1073|203|674|876|PF02786.17, 302_1073|126|548|673|UNK3, 313_1073|127|1|127|UNK1, 453_1073|121|427|547|PF02787.19, 458_1073|92|335|426|UNK2, 866_1073|80|877|956|UNK4, 1073|85|957|1041|PF02142.22, 1073|32|1042|1073|UNK5

Secondary: sp|P0A6F1|CARA_ECOLI Carbamoyl-phosphate synthase small chain OS=Escherichia coli (strain K12) OX=83333 GN=carA PE=1 SV=1

233_382|176|197|372|PF00117.28, 369_382|128|5|132|PF00988.22, 382|64|133|196|UNK1

Primary: sp|P25888|RHLE_ECOLI ATP-dependent RNA helicase RhlE OS=Escherichia coli (strain K12) OX=83333 GN=rhlE PE=1 SV=3

206_454|172|25|196|PF00270.29, 420_454|99|241|339|PF00271.31, 454|44|197|240|UNK1, 454|115|340|454|UNK2

Secondary: sp|O00571|DDX3X_HUMAN ATP-dependent RNA helicase DDX3X OS=Homo sapiens OX=9606 GN=DDX3X PE=1 SV=3

420_662|111|426|536|PF00271.31, 662|188|204|391|PF00270.29, 662|203|1|203|UNK1, 662|34|392|425|UNK2, 662|126|537|662|UNK3

Secondary: sp|O15523|DDX3Y_HUMAN ATP-dependent RNA helicase DDX3Y OS=Homo sapiens OX=9606 GN=DDX3Y PE=1 SV=2

420_660|110|425|534|PF00271.31, 660|188|202|389|PF00270.29, 660|201|1|201|UNK1, 660|35|390|424|UNK2, 660|126|535|660|UNK3

Secondary: sp|Q7L014|DDX46_HUMAN Probable ATP-dependent RNA helicase DDX46 OS=Homo sapiens OX=9606 GN=DDX46 PE=1 SV=2

206_1031|173|396|568|PF00270.29, 1031|110|605|714|PF00271.31, 1031|395|1|395|UNK1, 1031|36|569|604|UNK2, 1031|317|715|1031|UNK3

Secondary: sp|Q86TM3|DDX53_HUMAN Probable ATP-dependent RNA helicase DDX53 OS=Homo sapiens OX=9606 GN=DDX53 PE=1 SV=3

206_631|171|246|416|PF00270.29, 631|58|53|110|PF00013.29, 631|106|457|562|PF00271.31, 631|52|1|52|UNK1, 631|135|111|245|UNK2, 631|40|417|456|UNK3, 631|69|563|631|UNK4

Secondary: sp|Q86XP3|DDX42_HUMAN ATP-dependent RNA helicase DDX42 OS=Homo sapiens OX=9606 GN=DDX42 PE=1 SV=1

206_938|172|277|448|PF00270.29, 415_938|106|488|593|PF00271.31, 938|276|1|276|UNK1, 938|39|449|487|UNK2, 938|345|594|938|UNK3

Secondary: sp|Q9BUQ8|DDX23_HUMAN Probable ATP-dependent RNA helicase DDX23 OS=Homo sapiens OX=9606 GN=DDX23 PE=1 SV=3

206_820|202|415|616|PF00270.29, 399_820|109|651|759|PF00271.31, 820|414|1|414|UNK1, 820|34|617|650|UNK2, 820|61|760|820|UNK3

Secondary: sp|Q9NQI0|DDX4_HUMAN Probable ATP-dependent RNA helicase DDX4 OS=Homo sapiens OX=9606 GN=DDX4 PE=1 SV=2

420_724|109|527|635|PF00271.31, 724|180|312|491|PF00270.29, 724|311|1|311|UNK1, 724|35|492|526|UNK2, 724|89|636|724|UNK3

Secondary: sp|Q9UJV9|DDX41_HUMAN Probable ATP-dependent RNA helicase DDX41 OS=Homo sapiens OX=9606 GN=DDX41 PE=1 SV=2

420_622|108|420|527|PF00271.31, 622|180|205|384|PF00270.29, 622|204|1|204|UNK1, 622|35|385|419|UNK2, 622|95|528|622|UNK3

Primary: sp|P31552|CAIC_ECOLI Crotonobetaine/carnitine--CoA ligase OS=Escherichia coli (strain K12) OX=83333 GN=caiC PE=1 SV=2

291_517|410|17|426|PF00501.28, 646_517|76|435|510|PF13193.6

Secondary: sp|O14975|S27A2_HUMAN Very long-chain acyl-CoA synthetase OS=Homo sapiens OX=9606 GN=SLC27A2 PE=1 SV=2

291_620|428|60|487|PF00501.28, 620|74|499|572|PF13193.6, 620|59|1|59|UNK1, 620|48|573|620|UNK2

Secondary: sp|Q5K4L6|S27A3_HUMAN Long-chain fatty acid transport protein 3 OS=Homo sapiens OX=9606 GN=SLC27A3 PE=2 SV=3

291_730|386|212|597|PF00501.28, 730|77|606|682|PF13193.6, 730|211|1|211|UNK1, 730|48|683|730|UNK2

Secondary: sp|Q6P1M0|S27A4_HUMAN Long-chain fatty acid transport protein 4 OS=Homo sapiens OX=9606 GN=SLC27A4 PE=1 SV=1

291_643|431|81|511|PF00501.28, 643|76|520|595|PF13193.6, 643|80|1|80|UNK1, 643|48|596|643|UNK2

Secondary: sp|Q6PCB7|S27A1_HUMAN Long-chain fatty acid transport protein 1 OS=Homo sapiens OX=9606 GN=SLC27A1 PE=2 SV=1

291_646|434|82|515|PF00501.28, 646|76|523|598|PF13193.6, 646|81|1|81|UNK1, 646|48|599|646|UNK2

Secondary: sp|Q96CM8|ACSF2_HUMAN Acyl-CoA synthetase family member 2, mitochondrial OS=Homo sapiens OX=9606 GN=ACSF2 PE=1 SV=2

191_615|437|79|515|PF00501.28, 646_615|76|524|599|PF13193.6, 615|78|1|78|UNK1

Secondary: sp|Q9Y2P4|S27A6_HUMAN Long-chain fatty acid transport protein 6 OS=Homo sapiens OX=9606 GN=SLC27A6 PE=2 SV=1

291_619|425|62|486|PF00501.28, 619|77|495|571|PF13193.6, 619|61|1|61|UNK1, 619|48|572|619|UNK2

Secondary: sp|Q9Y2P5|S27A5_HUMAN Bile acyl-CoA synthetase OS=Homo sapiens OX=9606 GN=SLC27A5 PE=1 SV=1

291_690|438|120|557|PF00501.28, 690|77|566|642|PF13193.6, 690|119|1|119|UNK1, 690|48|643|690|UNK2

Primary: sp|P78363|ABCA4_HUMAN Retinal-specific ATP-binding cassette transporter OS=Homo sapiens OX=9606 GN=ABCA4 PE=1 SV=3

443_2273|144|947|1090|PF00005.27, 443_2273|142|1957|2098|PF00005.27, 841_2273|175|2099|2273|UNK5, 2273|247|610|856|PF12698.7, 2273|292|1604|1895|PF12698.7, 2273|609|1|609|UNK1, 2273|90|857|946|UNK2, 2273|513|1091|1603|UNK3, 2273|61|1896|1956|UNK4

Secondary: sp|P0A9U1|YBHF_ECOLI Probable multidrug ABC transporter ATP-binding protein YbhF OS=Escherichia coli (strain K12) OX=83333 GN=ybhF PE=1 SV=1

400_578|144|346|489|PF00005.27, 443_578|140|26|165|PF00005.27, 888_578|89|490|578|UNK2, 578|180|166|345|UNK1

Secondary: sp|P36879|YADG_ECOLI Uncharacterized ABC transporter ATP-binding protein YadG OS=Escherichia coli (strain K12) OX=83333 GN=yadG PE=1 SV=1

417_308|145|21|165|PF00005.27, 841_308|143|166|308|UNK1

Secondary: sp|P37624|RBBA_ECOLI Ribosome-associated ATPase OS=Escherichia coli (strain K12) OX=83333 GN=rbbA PE=1 SV=3

400_911|144|293|436|PF00005.27, 443_911|148|28|175|PF00005.27, 888_911|132|437|568|UNK2, 888_911|117|176|292|UNK1, 911|335|569|903|PF12698.7

Primary: sp|Q9NWZ5|UCKL1_HUMAN Uridine-cytidine kinase-like 1 OS=Homo sapiens OX=9606 GN=UCKL1 PE=1 SV=2

207_548|187|101|287|PF00485.18, 550_548|204|329|532|PF14681.6, 548|100|1|100|UNK1, 548|41|288|328|UNK2

Secondary: sp|P0A8F0|UPP_ECOLI Uracil phosphoribosyltransferase OS=Escherichia coli (strain K12) OX=83333 GN=upp PE=1 SV=1

550_208|201|7|207|PF14681.6

Secondary: sp|P0A8F4|URK_ECOLI Uridine kinase OS=Escherichia coli (strain K12) OX=83333 GN=udk PE=3 SV=1

207_213|190|10|199|PF00485.18

Primary: sp|Q9BYC2|SCOT2_HUMAN Succinyl-CoA:3-ketoacid coenzyme A transferase 2, mitochondrial OS=Homo sapiens OX=9606 GN=OXCT2 PE=2 SV=2

149_517|197|301|497|PF01144.23, 183_517|225|47|271|PF01144.23, 517|46|1|46|UNK1

Secondary: sp|P76458|ATOD_ECOLI Acetate CoA-transferase subunit alpha OS=Escherichia coli (strain K12) OX=83333 GN=atoD PE=1 SV=1

183_220|210|6|215|PF01144.23

Secondary: sp|P76459|ATOA_ECOLI Acetate CoA-transferase subunit beta OS=Escherichia coli (strain K12) OX=83333 GN=atoA PE=1 SV=1

149_216|175|5|179|PF01144.23, 216|37|180|216|UNK1

Primary: sp|Q3LXA3|TKFC_HUMAN Triokinase/FMN cyclase OS=Homo sapiens OX=9606 GN=TKFC PE=1 SV=2

108_575|315|20|334|PF02733.17, 555_575|172|399|570|PF02734.17, 575|64|335|398|UNK1

Secondary: sp|P76014|DHAL_ECOLI PEP-dependent dihydroxyacetone kinase, ADP-binding subunit DhaL OS=Escherichia coli (strain K12) OX=83333 GN=dhaL PE=1 SV=3

555_210|173|32|204|PF02734.17, 210|31|1|31|UNK1

Secondary: sp|P76015|DHAK_ECOLI PEP-dependent dihydroxyacetone kinase, dihydroxyacetone-binding subunit DhaK OS=Escherichia coli (strain K12) OX=83333 GN=dhaK PE=1 SV=2

108_356|334|18|351|PF02733.17

Primary: sp|P27708|PYR1_HUMAN CAD protein OS=Homo sapiens OX=9606 GN=CAD PE=1 SV=3

114_2225|204|514|717|PF02786.17, 187_2225|198|1050|1247|PF02786.17, 233_2225|176|180|355|PF00117.28, 302_2225|128|922|1049|UNK4, 306_2225|141|1925|2065|PF02729.21, 313_2225|158|356|513|UNK2, 315_2225|149|2072|2220|PF00185.24, 369_2225|136|2|137|PF00988.22, 453_2225|121|801|921|PF02787.19, 866_2225|79|1248|1326|UNK5, 2225|101|1327|1427|PF02142.22, 2225|61|1464|1524|PF01979.20, 2225|42|138|179|UNK1, 2225|83|718|800|UNK3, 2225|36|1428|1463|UNK6, 2225|400|1525|1924|UNK7

Secondary: sp|P00968|CARB_ECOLI Carbamoyl-phosphate synthase large chain OS=Escherichia coli (strain K12) OX=83333 GN=carB PE=1 SV=2

114_1073|207|128|334|PF02786.17, 187_1073|203|674|876|PF02786.17, 302_1073|126|548|673|UNK3, 313_1073|127|1|127|UNK1, 453_1073|121|427|547|PF02787.19, 458_1073|92|335|426|UNK2, 866_1073|80|877|956|UNK4, 1073|85|957|1041|PF02142.22, 1073|32|1042|1073|UNK5

Secondary: sp|P0A6F1|CARA_ECOLI Carbamoyl-phosphate synthase small chain OS=Escherichia coli (strain K12) OX=83333 GN=carA PE=1 SV=1

233_382|176|197|372|PF00117.28, 369_382|128|5|132|PF00988.22, 382|64|133|196|UNK1

Secondary: sp|P0A786|PYRB_ECOLI Aspartate carbamoyltransferase catalytic subunit OS=Escherichia coli (strain K12) OX=83333 GN=pyrB PE=1 SV=2

306_311|141|8|148|PF02729.21, 315_311|149|155|303|PF00185.24

Primary: sp|Q96NN9|AIFM3_HUMAN Apoptosis-inducing factor 3 OS=Homo sapiens OX=9606 GN=AIFM3 PE=1 SV=1

276_605|298|196|493|PF07992.14, 860_605|84|71|154|PF00355.26, 605|72|512|583|PF14759.6, 605|70|1|70|UNK1, 605|41|155|195|UNK2

Secondary: sp|P0ABW0|HCAC_ECOLI 3-phenylpropionate/cinnamic acid dioxygenase ferredoxin subunit OS=Escherichia coli (strain K12) OX=83333 GN=hcaC PE=1 SV=1

860_106|86|4|89|PF00355.26

Secondary: sp|P77650|HCAD_ECOLI 3-phenylpropionate/cinnamic acid dioxygenase ferredoxin--NAD(+) reductase component OS=Escherichia coli (strain K12) OX=83333 GN=hcaD PE=1 SV=1

276_400|295|5|299|PF07992.14, 400|83|318|400|PF14759.6

Primary: sp|P0A9P6|DEAD_ECOLI ATP-dependent RNA helicase DeaD OS=Escherichia coli (strain K12) OX=83333 GN=deaD PE=1 SV=2

218_629|165|31|195|PF00270.29, 399_629|109|232|340|PF00271.31, 629|70|489|558|PF03880.15, 629|62|568|629|PF12343.8, 629|30|1|30|UNK1, 629|36|196|231|UNK2, 629|148|341|488|UNK3

Secondary: sp|Q9BQ39|DDX50_HUMAN ATP-dependent RNA helicase DDX50 OS=Homo sapiens OX=9606 GN=DDX50 PE=1 SV=1

218_737|175|161|335|PF00270.29, 737|97|387|483|PF00271.31, 737|95|571|665|PF08152.12, 737|160|1|160|UNK1, 737|51|336|386|UNK2, 737|87|484|570|UNK3, 737|72|666|737|UNK4

Secondary: sp|Q9BUQ8|DDX23_HUMAN Probable ATP-dependent RNA helicase DDX23 OS=Homo sapiens OX=9606 GN=DDX23 PE=1 SV=3

206_820|202|415|616|PF00270.29, 399_820|109|651|759|PF00271.31, 820|414|1|414|UNK1, 820|34|617|650|UNK2, 820|61|760|820|UNK3

Secondary: sp|Q9NR30|DDX21_HUMAN Nucleolar RNA helicase 2 OS=Homo sapiens OX=9606 GN=DDX21 PE=1 SV=5

218_783|171|210|380|PF00270.29, 783|97|436|532|PF00271.31, 783|96|620|715|PF08152.12, 783|209|1|209|UNK1, 783|55|381|435|UNK2, 783|87|533|619|UNK3, 783|68|716|783|UNK4

Primary: sp|Q8NE71|ABCF1_HUMAN ATP-binding cassette sub-family F member 1 OS=Homo sapiens OX=9606 GN=ABCF1 PE=1 SV=2

364_845|160|321|480|PF00005.27, 377_845|130|642|771|PF00005.27, 814_845|38|481|518|UNK2, 833_845|74|772|845|UNK4, 845|76|519|594|PF12848.7, 845|320|1|320|UNK1, 845|47|595|641|UNK3

Secondary: sp|P0A9U3|YBIT_ECOLI Uncharacterized ABC transporter ATP-binding protein YbiT OS=Escherichia coli (strain K12) OX=83333 GN=ybiT PE=1 SV=1

833_530|63|468|530|UNK2, 530|167|18|184|PF00005.27, 530|86|224|309|PF12848.7, 530|132|336|467|PF00005.27, 530|39|185|223|UNK1

Secondary: sp|P63389|YHES_ECOLI Uncharacterized ABC transporter ATP-binding protein YheS OS=Escherichia coli (strain K12) OX=83333 GN=yheS PE=1 SV=1

364_637|162|17|178|PF00005.27, 377_637|132|328|459|PF00005.27, 612_637|82|217|298|PF12848.7, 814_637|38|179|216|UNK1, 637|178|460|637|UNK2

Primary: sp|P0A6A0|UBIB_ECOLI Probable protein kinase UbiB OS=Escherichia coli (strain K12) OX=83333 GN=ubiB PE=1 SV=1

664_546|314|233|546|UNK2, 692_546|117|116|232|PF03109.16, 546|115|1|115|UNK1

Secondary: sp|Q7Z695|ADCK2_HUMAN Uncharacterized aarF domain-containing protein kinase 2 OS=Homo sapiens OX=9606 GN=ADCK2 PE=2 SV=1

664_626|239|388|626|UNK2, 626|96|292|387|PF03109.16, 626|291|1|291|UNK1

Secondary: sp|Q8NI60|COQ8A_HUMAN Atypical kinase COQ8A, mitochondrial OS=Homo sapiens OX=9606 GN=COQ8A PE=1 SV=1

692_647|115|319|433|PF03109.16, 647|318|1|318|UNK1, 647|214|434|647|UNK2

Secondary: sp|Q96D53|COQ8B_HUMAN Atypical kinase COQ8B, mitochondrial OS=Homo sapiens OX=9606 GN=COQ8B PE=1 SV=2

692_544|116|197|312|PF03109.16, 544|196|1|196|UNK1, 544|232|313|544|UNK2

Primary: sp|P60584|CAIA_ECOLI Crotonobetainyl-CoA reductase OS=Escherichia coli (strain K12) OX=83333 GN=caiA PE=1 SV=1

427_380|150|227|376|PF00441.24, 661_380|93|123|215|PF02770.19, 889_380|103|6|108|PF02771.16

Secondary: sp|P26440|IVD_HUMAN Isovaleryl-CoA dehydrogenase, mitochondrial OS=Homo sapiens OX=9606 GN=IVD PE=1 SV=1

661_423|98|161|258|PF02770.19, 889_423|115|43|157|PF02771.16, 423|148|270|417|PF00441.24, 423|42|1|42|UNK1

Secondary: sp|P28330|ACADL_HUMAN Long-chain specific acyl-CoA dehydrogenase, mitochondrial OS=Homo sapiens OX=9606 GN=ACADL PE=1 SV=2

661_430|97|169|265|PF02770.19, 430|111|54|164|PF02771.16, 430|149|278|426|PF00441.24, 430|53|1|53|UNK1

Secondary: sp|P49748|ACADV_HUMAN Very long-chain specific acyl-CoA dehydrogenase, mitochondrial OS=Homo sapiens OX=9606 GN=ACADVL PE=1 SV=1

661_655|103|213|315|PF02770.19, 803_655|114|96|209|PF02771.16, 655|147|327|473|PF00441.24, 655|95|1|95|UNK1, 655|182|474|655|UNK2

Secondary: sp|Q92947|GCDH_HUMAN Glutaryl-CoA dehydrogenase, mitochondrial OS=Homo sapiens OX=9606 GN=GCDH PE=1 SV=1

889_438|111|62|172|PF02771.16, 438|94|176|269|PF02770.19, 438|140|289|428|PF00441.24, 438|61|1|61|UNK1

Secondary: sp|Q9H845|ACAD9_HUMAN Acyl-CoA dehydrogenase family member 9, mitochondrial OS=Homo sapiens OX=9606 GN=ACAD9 PE=1 SV=1

661_621|101|177|277|PF02770.19, 803_621|106|68|173|PF02771.16, 621|148|290|437|PF00441.24, 621|67|1|67|UNK1, 621|184|438|621|UNK2

Primary: sp|Q96CM8|ACSF2_HUMAN Acyl-CoA synthetase family member 2, mitochondrial OS=Homo sapiens OX=9606 GN=ACSF2 PE=1 SV=2

191_615|437|79|515|PF00501.28, 646_615|76|524|599|PF13193.6, 615|78|1|78|UNK1

Secondary: sp|P31552|CAIC_ECOLI Crotonobetaine/carnitine--CoA ligase OS=Escherichia coli (strain K12) OX=83333 GN=caiC PE=1 SV=2

291_517|410|17|426|PF00501.28, 646_517|76|435|510|PF13193.6

Secondary: sp|P69451|LCFA_ECOLI Long-chain-fatty-acid--CoA ligase OS=Escherichia coli (strain K12) OX=83333 GN=fadD PE=1 SV=1

191_561|432|29|460|PF00501.28, 561|75|469|543|PF13193.6

Primary: sp|Q00796|DHSO_HUMAN Sorbitol dehydrogenase OS=Homo sapiens OX=9606 GN=SORD PE=1 SV=4

435_357|111|32|142|PF08240.12, 587_357|130|183|312|PF00107.26, 852_357|40|143|182|UNK2, 357|31|1|31|UNK1, 357|45|313|357|UNK3

Secondary: sp|P39346|IDND_ECOLI L-idonate 5-dehydrogenase (NAD(P)(+)) OS=Escherichia coli (strain K12) OX=83333 GN=idnD PE=1 SV=1

587_343|125|180|304|PF00107.26, 343|110|30|139|PF08240.12, 343|40|140|179|UNK1, 343|39|305|343|UNK2

Secondary: sp|P77280|YDJJ_ECOLI Uncharacterized zinc-type alcohol dehydrogenase-like protein YdjJ OS=Escherichia coli (strain K12) OX=83333 GN=ydjJ PE=1 SV=1

435_347|113|27|139|PF08240.12, 852_347|40|140|179|UNK1, 347|120|180|299|PF00107.26, 347|48|300|347|UNK2

Primary: sp|P60422|RL2_ECOLI 50S ribosomal protein L2 OS=Escherichia coli (strain K12) OX=83333 GN=rplB PE=1 SV=2

521_273|125|125|249|PF03947.18, 657_273|76|42|117|PF00181.23, 273|41|1|41|UNK1

Secondary: sp|P62917|RL8_HUMAN 60S ribosomal protein L8 OS=Homo sapiens OX=9606 GN=RPL8 PE=1 SV=2

521_257|134|97|230|PF03947.18, 257|78|13|90|PF00181.23

Secondary: sp|Q5T653|RM02_HUMAN 39S ribosomal protein L2, mitochondrial OS=Homo sapiens OX=9606 GN=MRPL2 PE=1 SV=2

657_305|81|84|164|PF00181.23, 305|94|178|271|PF03947.18, 305|83|1|83|UNK1, 305|34|272|305|UNK2

Primary: sp|O75417|DPOLQ_HUMAN DNA polymerase theta OS=Homo sapiens OX=9606 GN=POLQ PE=1 SV=2

85_2590|488|2097|2584|PF00476.20, 789_2590|82|404|485|PF00271.31, 2590|167|103|269|PF00270.29, 2590|102|1|102|UNK1, 2590|134|270|403|UNK2, 2590|1611|486|2096|UNK3

Secondary: sp|P00582|DPO1_ECOLI DNA polymerase I OS=Escherichia coli (strain K12) OX=83333 GN=polA PE=1 SV=1

85_928|374|552|925|PF00476.20, 928|162|9|170|PF02739.16, 928|101|171|271|PF01367.20, 928|187|330|516|PF01612.20, 928|58|272|329|UNK1, 928|35|517|551|UNK2

Secondary: sp|P24230|RECG_ECOLI ATP-dependent DNA helicase RecG OS=Escherichia coli (strain K12) OX=83333 GN=recG PE=1 SV=1

789_693|108|481|588|PF00271.31, 693|157|15|171|PF17191.4, 693|161|273|433|PF00270.29, 693|101|172|272|UNK1, 693|47|434|480|UNK2, 693|105|589|693|UNK3

Secondary: sp|P30958|MFD_ECOLI Transcription-repair-coupling factor OS=Escherichia coli (strain K12) OX=83333 GN=mfd PE=1 SV=2

789_1148|104|803|906|PF00271.31, 1148|89|129|217|PF17757.1, 1148|96|477|572|PF02559.16, 1148|161|602|762|PF00270.29, 1148|93|1006|1098|PF03461.15, 1148|128|1|128|UNK1, 1148|259|218|476|UNK2, 1148|40|763|802|UNK3, 1148|99|907|1005|UNK4, 1148|50|1099|1148|UNK5

Primary: sp|P05055|PNP_ECOLI Polyribonucleotide nucleotidyltransferase OS=Escherichia coli (strain K12) OX=83333 GN=pnp PE=1 SV=3

293_711|133|324|456|PF01138.21, 354_711|129|16|144|PF01138.21, 567_711|68|460|527|PF03725.15, 749_711|64|147|210|PF03725.15, 755_711|72|619|690|PF00575.23, 711|80|241|320|PF03726.14, 711|59|556|614|PF00013.29, 711|30|211|240|UNK1

Secondary: sp|Q14562|DHX8_HUMAN ATP-dependent RNA helicase DHX8 OS=Homo sapiens OX=9606 GN=DHX8 PE=1 SV=1

381_1220|129|767|895|PF00271.31, 687_1220|89|957|1045|PF04408.23, 755_1220|74|261|334|PF00575.23, 862_1220|77|1103|1179|PF07717.16, 1220|260|1|260|UNK1, 1220|432|335|766|UNK2, 1220|61|896|956|UNK3, 1220|57|1046|1102|UNK4, 1220|41|1180|1220|UNK5

Secondary: sp|Q8TCS8|PNPT1_HUMAN Polyribonucleotide nucleotidyltransferase 1, mitochondrial OS=Homo sapiens OX=9606 GN=PNPT1 PE=1 SV=2

293_783|135|367|501|PF01138.21, 354_783|130|54|183|PF01138.21, 567_783|106|502|607|UNK3, 749_783|65|186|250|PF03725.15, 783|78|286|363|PF03726.14, 783|58|608|665|PF00013.29, 783|73|678|750|PF00575.23, 783|53|1|53|UNK1, 783|35|251|285|UNK2, 783|33|751|783|UNK4

Primary: sp|Q13423|NNTM_HUMAN NAD(P) transhydrogenase, mitochondrial OS=Homo sapiens OX=9606 GN=NNT PE=1 SV=3

14_1086|458|621|1078|PF02233.16, 69_1086|233|203|435|PF01262.21, 266_1086|140|60|199|PF05222.15, 472_1086|87|501|587|PF12769.7, 1086|59|1|59|UNK1, 1086|65|436|500|UNK2, 1086|33|588|620|UNK3

Secondary: sp|P07001|PNTA_ECOLI NAD(P) transhydrogenase subunit alpha OS=Escherichia coli (strain K12) OX=83333 GN=pntA PE=1 SV=2

69_510|228|141|368|PF01262.21, 266_510|134|4|137|PF05222.15, 472_510|83|427|509|PF12769.7, 510|58|369|426|UNK1

Secondary: sp|P0AB67|PNTB_ECOLI NAD(P) transhydrogenase subunit beta OS=Escherichia coli (strain K12) OX=83333 GN=pntB PE=1 SV=1

14_462|454|7|460|PF02233.16

Primary: sp|P25437|FRMA_ECOLI S-(hydroxymethyl)glutathione dehydrogenase OS=Escherichia coli (strain K12) OX=83333 GN=frmA PE=1 SV=3

152_369|127|28|154|PF08240.12, 175_369|123|197|319|PF00107.26, 686_369|42|155|196|UNK1, 713_369|50|320|369|UNK2

Secondary: sp|P00325|ADH1B_HUMAN Alcohol dehydrogenase 1B OS=Homo sapiens OX=9606 GN=ADH1B PE=1 SV=2

686_375|42|161|202|UNK2, 713_375|50|326|375|UNK3, 375|125|36|160|PF08240.12, 375|123|203|325|PF00107.26, 375|35|1|35|UNK1

Secondary: sp|P00326|ADH1G_HUMAN Alcohol dehydrogenase 1C OS=Homo sapiens OX=9606 GN=ADH1C PE=1 SV=2

175_375|129|203|331|PF00107.26, 686_375|42|161|202|UNK2, 375|126|35|160|PF08240.12, 375|34|1|34|UNK1, 375|44|332|375|UNK3

Secondary: sp|P07327|ADH1A_HUMAN Alcohol dehydrogenase 1A OS=Homo sapiens OX=9606 GN=ADH1A PE=1 SV=2

686_375|42|161|202|UNK2, 713_375|47|329|375|UNK3, 375|126|35|160|PF08240.12, 375|126|203|328|PF00107.26, 375|34|1|34|UNK1

Secondary: sp|P08319|ADH4_HUMAN Alcohol dehydrogenase 4 OS=Homo sapiens OX=9606 GN=ADH4 PE=1 SV=5

686_380|43|165|207|UNK2, 713_380|50|331|380|UNK3, 380|130|35|164|PF08240.12, 380|123|208|330|PF00107.26, 380|34|1|34|UNK1

Secondary: sp|P28332|ADH6_HUMAN Alcohol dehydrogenase 6 OS=Homo sapiens OX=9606 GN=ADH6 PE=1 SV=2

686_368|42|161|202|UNK2, 368|126|35|160|PF08240.12, 368|125|203|327|PF00107.26, 368|34|1|34|UNK1, 368|41|328|368|UNK3

Secondary: sp|P40394|ADH7_HUMAN Alcohol dehydrogenase class 4 mu/sigma chain OS=Homo sapiens OX=9606 GN=ADH7 PE=1 SV=2

686_386|42|172|213|UNK2, 386|125|47|171|PF08240.12, 386|131|214|344|PF00107.26, 386|46|1|46|UNK1, 386|42|345|386|UNK3

Primary: sp|P27550|ACSA_ECOLI Acetyl-coenzyme A synthetase OS=Escherichia coli (strain K12) OX=83333 GN=acs PE=1 SV=2

7_652|440|83|522|PF00501.28, 452_652|79|531|609|PF13193.6, 878_652|43|610|652|UNK1, 652|58|24|81|PF16177.5

Secondary: sp|Q9NR19|ACSA_HUMAN Acetyl-coenzyme A synthetase, cytoplasmic OS=Homo sapiens OX=9606 GN=ACSS2 PE=1 SV=1

7_701|460|115|574|PF00501.28, 452_701|79|583|661|PF13193.6, 701|61|47|107|PF16177.5, 701|46|1|46|UNK1, 701|40|662|701|UNK2

Secondary: sp|Q9NUB1|ACS2L_HUMAN Acetyl-coenzyme A synthetase 2-like, mitochondrial OS=Homo sapiens OX=9606 GN=ACSS1 PE=1 SV=2

878_689|47|643|689|UNK2, 689|57|58|114|PF16177.5, 689|440|116|555|PF00501.28, 689|79|564|642|PF13193.6, 689|57|1|57|UNK1

Primary: sp|Q06278|AOXA_HUMAN Aldehyde oxidase OS=Homo sapiens OX=9606 GN=AOX1 PE=1 SV=2

217_1338|529|717|1245|PF02738.18, 652_1338|74|88|161|PF01799.20, 737_1338|178|241|418|PF00941.21, 801_1338|107|594|700|PF01315.22, 1338|69|10|78|PF00111.27, 1338|105|426|530|PF03450.17, 1338|79|162|240|UNK1, 1338|63|531|593|UNK2, 1338|93|1246|1338|UNK3

Secondary: sp|Q46799|XDHA_ECOLI Putative xanthine dehydrogenase molybdenum-binding subunit XdhA OS=Escherichia coli (strain K12) OX=83333 GN=xdhA PE=2 SV=1

217_752|543|145|687|PF02738.18, 801_752|117|10|126|PF01315.22, 752|65|688|752|UNK1

Secondary: sp|Q46800|XDHB_ECOLI Putative xanthine dehydrogenase FAD-binding subunit XdhB OS=Escherichia coli (strain K12) OX=83333 GN=xdhB PE=4 SV=1

737_292|168|6|173|PF00941.21, 292|101|183|283|PF03450.17

Secondary: sp|Q46801|XDHC_ECOLI Putative xanthine dehydrogenase iron-sulfur-binding subunit XdhC OS=Escherichia coli (strain K12) OX=83333 GN=xdhC PE=3 SV=1

652_159|75|78|152|PF01799.20, 159|53|12|64|PF00111.27

Secondary: sp|Q46814|XDHD_ECOLI Probable hypoxanthine oxidase XdhD OS=Escherichia coli (strain K12) OX=83333 GN=xdhD PE=3 SV=1

217_956|539|355|893|PF02738.18, 801_956|111|187|297|PF01315.22, 956|72|73|144|PF01799.20, 956|72|1|72|UNK1, 956|42|145|186|UNK2, 956|57|298|354|UNK3, 956|63|894|956|UNK4

Primary: sp|Q9NRK6|ABCBA_HUMAN ATP-binding cassette sub-family B member 10, mitochondrial OS=Homo sapiens OX=9606 GN=ABCB10 PE=1 SV=2

213_738|152|511|662|PF00005.27, 462_738|264|175|438|PF00664.23, 738|174|1|174|UNK1, 738|72|439|510|UNK2, 738|76|663|738|UNK3

Secondary: sp|P0AAG5|MDLB_ECOLI Multidrug resistance-like ATP-binding protein MdlB OS=Escherichia coli (strain K12) OX=83333 GN=mdlB PE=1 SV=1

462_593|270|26|295|PF00664.23, 593|149|357|505|PF00005.27, 593|61|296|356|UNK1, 593|88|506|593|UNK2

Secondary: sp|P60752|MSBA_ECOLI Lipid A export ATP-binding/permease protein MsbA OS=Escherichia coli (strain K12) OX=83333 GN=msbA PE=1 SV=1

213_582|151|359|509|PF00005.27, 468_582|73|510|582|UNK2, 582|271|27|297|PF00664.23, 582|61|298|358|UNK1

Primary: sp|O94911|ABCA8_HUMAN ATP-binding cassette sub-family A member 8 OS=Homo sapiens OX=9606 GN=ABCA8 PE=1 SV=3

400_1581|139|1267|1405|PF00005.27, 841_1581|176|1406|1581|UNK5, 1581|381|38|418|PF12698.7, 1581|121|499|619|PF00005.27, 1581|240|901|1140|PF12698.7, 1581|37|1|37|UNK1, 1581|80|419|498|UNK2, 1581|281|620|900|UNK3, 1581|126|1141|1266|UNK4

Secondary: sp|P0A9U1|YBHF_ECOLI Probable multidrug ABC transporter ATP-binding protein YbhF OS=Escherichia coli (strain K12) OX=83333 GN=ybhF PE=1 SV=1

400_578|144|346|489|PF00005.27, 443_578|140|26|165|PF00005.27, 888_578|89|490|578|UNK2, 578|180|166|345|UNK1

Secondary: sp|P36879|YADG_ECOLI Uncharacterized ABC transporter ATP-binding protein YadG OS=Escherichia coli (strain K12) OX=83333 GN=yadG PE=1 SV=1

417_308|145|21|165|PF00005.27, 841_308|143|166|308|UNK1

Secondary: sp|P37624|RBBA_ECOLI Ribosome-associated ATPase OS=Escherichia coli (strain K12) OX=83333 GN=rbbA PE=1 SV=3

400_911|144|293|436|PF00005.27, 443_911|148|28|175|PF00005.27, 888_911|132|437|568|UNK2, 888_911|117|176|292|UNK1, 911|335|569|903|PF12698.7

Primary: sp|Q9BUQ8|DDX23_HUMAN Probable ATP-dependent RNA helicase DDX23 OS=Homo sapiens OX=9606 GN=DDX23 PE=1 SV=3

206_820|202|415|616|PF00270.29, 399_820|109|651|759|PF00271.31, 820|414|1|414|UNK1, 820|34|617|650|UNK2, 820|61|760|820|UNK3

Secondary: sp|P0A9P6|DEAD_ECOLI ATP-dependent RNA helicase DeaD OS=Escherichia coli (strain K12) OX=83333 GN=deaD PE=1 SV=2

218_629|165|31|195|PF00270.29, 399_629|109|232|340|PF00271.31, 629|70|489|558|PF03880.15, 629|62|568|629|PF12343.8, 629|30|1|30|UNK1, 629|36|196|231|UNK2, 629|148|341|488|UNK3

Secondary: sp|P25888|RHLE_ECOLI ATP-dependent RNA helicase RhlE OS=Escherichia coli (strain K12) OX=83333 GN=rhlE PE=1 SV=3

206_454|172|25|196|PF00270.29, 420_454|99|241|339|PF00271.31, 454|44|197|240|UNK1, 454|115|340|454|UNK2

Primary: sp|P0A9U8|YDIO_ECOLI Probable acyl-CoA dehydrogenase YdiO OS=Escherichia coli (strain K12) OX=83333 GN=ydiO PE=3 SV=1

427_383|149|228|376|PF00441.24, 661_383|96|121|216|PF02770.19, 889_383|102|6|107|PF02771.16

Secondary: sp|P26440|IVD_HUMAN Isovaleryl-CoA dehydrogenase, mitochondrial OS=Homo sapiens OX=9606 GN=IVD PE=1 SV=1

661_423|98|161|258|PF02770.19, 889_423|115|43|157|PF02771.16, 423|148|270|417|PF00441.24, 423|42|1|42|UNK1

Secondary: sp|P28330|ACADL_HUMAN Long-chain specific acyl-CoA dehydrogenase, mitochondrial OS=Homo sapiens OX=9606 GN=ACADL PE=1 SV=2

661_430|97|169|265|PF02770.19, 430|111|54|164|PF02771.16, 430|149|278|426|PF00441.24, 430|53|1|53|UNK1

Secondary: sp|P49748|ACADV_HUMAN Very long-chain specific acyl-CoA dehydrogenase, mitochondrial OS=Homo sapiens OX=9606 GN=ACADVL PE=1 SV=1

661_655|103|213|315|PF02770.19, 803_655|114|96|209|PF02771.16, 655|147|327|473|PF00441.24, 655|95|1|95|UNK1, 655|182|474|655|UNK2

Secondary: sp|Q92947|GCDH_HUMAN Glutaryl-CoA dehydrogenase, mitochondrial OS=Homo sapiens OX=9606 GN=GCDH PE=1 SV=1

889_438|111|62|172|PF02771.16, 438|94|176|269|PF02770.19, 438|140|289|428|PF00441.24, 438|61|1|61|UNK1

Secondary: sp|Q9H845|ACAD9_HUMAN Acyl-CoA dehydrogenase family member 9, mitochondrial OS=Homo sapiens OX=9606 GN=ACAD9 PE=1 SV=1

661_621|101|177|277|PF02770.19, 803_621|106|68|173|PF02771.16, 621|148|290|437|PF00441.24, 621|67|1|67|UNK1, 621|184|438|621|UNK2

Primary: sp|P22234|PUR6_HUMAN Multifunctional protein ADE2 OS=Homo sapiens OX=9606 GN=PAICS PE=1 SV=3

357_425|225|12|236|PF01259.18, 805_425|138|267|404|PF00731.20, 425|30|237|266|UNK1

Secondary: sp|P0A7D7|PUR7_ECOLI Phosphoribosylaminoimidazole-succinocarboxamide synthase OS=Escherichia coli (strain K12) OX=83333 GN=purC PE=1 SV=1

357_237|225|7|231|PF01259.18

Secondary: sp|P0AG18|PURE_ECOLI N5-carboxyaminoimidazole ribonucleotide mutase OS=Escherichia coli (strain K12) OX=83333 GN=purE PE=1 SV=2

805_169|149|9|157|PF00731.20

Primary: sp|Q8WWZ4|ABCAA_HUMAN ATP-binding cassette sub-family A member 10 OS=Homo sapiens OX=9606 GN=ABCA10 PE=2 SV=3

400_1543|145|1223|1367|PF00005.27, 443_1543|147|410|556|PF00005.27, 841_1543|176|1368|1543|UNK5, 1543|240|90|329|PF12698.7, 1543|195|936|1130|PF12698.7, 1543|89|1|89|UNK1, 1543|80|330|409|UNK2, 1543|379|557|935|UNK3, 1543|92|1131|1222|UNK4

Secondary: sp|P0A9U1|YBHF_ECOLI Probable multidrug ABC transporter ATP-binding protein YbhF OS=Escherichia coli (strain K12) OX=83333 GN=ybhF PE=1 SV=1

400_578|144|346|489|PF00005.27, 443_578|140|26|165|PF00005.27, 888_578|89|490|578|UNK2, 578|180|166|345|UNK1

Secondary: sp|P36879|YADG_ECOLI Uncharacterized ABC transporter ATP-binding protein YadG OS=Escherichia coli (strain K12) OX=83333 GN=yadG PE=1 SV=1

417_308|145|21|165|PF00005.27, 841_308|143|166|308|UNK1

Secondary: sp|P37624|RBBA_ECOLI Ribosome-associated ATPase OS=Escherichia coli (strain K12) OX=83333 GN=rbbA PE=1 SV=3

400_911|144|293|436|PF00005.27, 443_911|148|28|175|PF00005.27, 888_911|132|437|568|UNK2, 888_911|117|176|292|UNK1, 911|335|569|903|PF12698.7

Primary: sp|Q9UBK8|MTRR_HUMAN Methionine synthase reductase OS=Homo sapiens OX=9606 GN=MTRR PE=1 SV=3

598_725|222|298|519|PF00667.20, 649_725|137|33|169|PF00258.25, 725|120|569|688|PF00175.21, 725|32|1|32|UNK1, 725|128|170|297|UNK2, 725|49|520|568|UNK3, 725|37|689|725|UNK4

Secondary: sp|P38038|CYSJ_ECOLI Sulfite reductase [NADPH] flavoprotein alpha-component OS=Escherichia coli (strain K12) OX=83333 GN=cysJ PE=1 SV=4

359_599|107|457|563|PF00175.21, 589_599|131|66|196|PF00258.25, 598_599|195|230|424|PF00667.20, 599|65|1|65|UNK1, 599|33|197|229|UNK2, 599|32|425|456|UNK3, 599|36|564|599|UNK4

Secondary: sp|P65367|YQCA_ECOLI Uncharacterized protein YqcA OS=Escherichia coli (strain K12) OX=83333 GN=yqcA PE=3 SV=1

649_149|135|6|140|PF00258.25

Primary: sp|P63389|YHES_ECOLI Uncharacterized ABC transporter ATP-binding protein YheS OS=Escherichia coli (strain K12) OX=83333 GN=yheS PE=1 SV=1

364_637|162|17|178|PF00005.27, 377_637|132|328|459|PF00005.27, 612_637|82|217|298|PF12848.7, 814_637|38|179|216|UNK1, 637|178|460|637|UNK2

Secondary: sp|Q8NE71|ABCF1_HUMAN ATP-binding cassette sub-family F member 1 OS=Homo sapiens OX=9606 GN=ABCF1 PE=1 SV=2

364_845|160|321|480|PF00005.27, 377_845|130|642|771|PF00005.27, 814_845|38|481|518|UNK2, 833_845|74|772|845|UNK4, 845|76|519|594|PF12848.7, 845|320|1|320|UNK1, 845|47|595|641|UNK3

Secondary: sp|Q9NUQ8|ABCF3_HUMAN ATP-binding cassette sub-family F member 3 OS=Homo sapiens OX=9606 GN=ABCF3 PE=1 SV=2

364_709|161|196|356|PF00005.27, 377_709|130|510|639|PF00005.27, 612_709|81|395|475|PF12848.7, 758_709|70|640|709|UNK4, 855_709|38|357|394|UNK2, 709|195|1|195|UNK1, 709|34|476|509|UNK3

Secondary: sp|Q9UG63|ABCF2_HUMAN ATP-binding cassette sub-family F member 2 OS=Homo sapiens OX=9606 GN=ABCF2 PE=1 SV=2

364_623|157|101|257|PF00005.27, 377_623|133|413|545|PF00005.27, 814_623|38|258|295|UNK2, 833_623|78|546|623|UNK4, 623|73|296|368|PF12848.7, 623|100|1|100|UNK1, 623|44|369|412|UNK3

Primary: sp|Q8WWZ7|ABCA5_HUMAN ATP-binding cassette sub-family A member 5 OS=Homo sapiens OX=9606 GN=ABCA5 PE=2 SV=2

400_1642|143|1318|1460|PF00005.27, 443_1642|147|497|643|PF00005.27, 841_1642|182|1461|1642|UNK5, 888_1642|222|644|865|UNK3, 1642|385|32|416|PF12698.7, 1642|359|866|1224|PF12698.7, 1642|31|1|31|UNK1, 1642|80|417|496|UNK2, 1642|93|1225|1317|UNK4

Secondary: sp|P0A9U1|YBHF_ECOLI Probable multidrug ABC transporter ATP-binding protein YbhF OS=Escherichia coli (strain K12) OX=83333 GN=ybhF PE=1 SV=1

400_578|144|346|489|PF00005.27, 443_578|140|26|165|PF00005.27, 888_578|89|490|578|UNK2, 578|180|166|345|UNK1

Secondary: sp|P36879|YADG_ECOLI Uncharacterized ABC transporter ATP-binding protein YadG OS=Escherichia coli (strain K12) OX=83333 GN=yadG PE=1 SV=1

417_308|145|21|165|PF00005.27, 841_308|143|166|308|UNK1

Secondary: sp|P37624|RBBA_ECOLI Ribosome-associated ATPase OS=Escherichia coli (strain K12) OX=83333 GN=rbbA PE=1 SV=3

400_911|144|293|436|PF00005.27, 443_911|148|28|175|PF00005.27, 888_911|132|437|568|UNK2, 888_911|117|176|292|UNK1, 911|335|569|903|PF12698.7

Primary: sp|P33599|NUOCD_ECOLI NADH-quinone oxidoreductase subunit C/D OS=Escherichia coli (strain K12) OX=83333 GN=nuoC PE=1 SV=3

103_596|272|325|596|PF00346.19, 389_596|151|174|324|UNK2, 537_596|128|46|173|PF00329.19, 596|45|1|45|UNK1

Secondary: sp|O75306|NDUS2_HUMAN NADH dehydrogenase [ubiquinone] iron-sulfur protein 2, mitochondrial OS=Homo sapiens OX=9606 GN=NDUFS2 PE=1 SV=2

103_463|271|193|463|PF00346.19, 389_463|192|1|192|UNK1

Secondary: sp|O75489|NDUS3_HUMAN NADH dehydrogenase [ubiquinone] iron-sulfur protein 3, mitochondrial OS=Homo sapiens OX=9606 GN=NDUFS3 PE=1 SV=1

537_264|121|87|207|PF00329.19, 264|86|1|86|UNK1, 264|57|208|264|UNK2

Primary: sp|Q9BZC7|ABCA2_HUMAN ATP-binding cassette sub-family A member 2 OS=Homo sapiens OX=9606 GN=ABCA2 PE=1 SV=3

443_2435|145|1007|1151|PF00005.27, 443_2435|142|2072|2213|PF00005.27, 841_2435|222|2214|2435|UNK5, 2435|208|705|912|PF12698.7, 2435|283|1725|2007|PF12698.7, 2435|704|1|704|UNK1, 2435|94|913|1006|UNK2, 2435|573|1152|1724|UNK3, 2435|64|2008|2071|UNK4

Secondary: sp|P0A9U1|YBHF_ECOLI Probable multidrug ABC transporter ATP-binding protein YbhF OS=Escherichia coli (strain K12) OX=83333 GN=ybhF PE=1 SV=1

400_578|144|346|489|PF00005.27, 443_578|140|26|165|PF00005.27, 888_578|89|490|578|UNK2, 578|180|166|345|UNK1

Secondary: sp|P36879|YADG_ECOLI Uncharacterized ABC transporter ATP-binding protein YadG OS=Escherichia coli (strain K12) OX=83333 GN=yadG PE=1 SV=1

417_308|145|21|165|PF00005.27, 841_308|143|166|308|UNK1

Secondary: sp|P37624|RBBA_ECOLI Ribosome-associated ATPase OS=Escherichia coli (strain K12) OX=83333 GN=rbbA PE=1 SV=3

400_911|144|293|436|PF00005.27, 443_911|148|28|175|PF00005.27, 888_911|132|437|568|UNK2, 888_911|117|176|292|UNK1, 911|335|569|903|PF12698.7

Primary: sp|O75643|U520_HUMAN U5 small nuclear ribonucleoprotein 200 kDa helicase OS=Homo sapiens OX=9606 GN=SNRNP200 PE=1 SV=2

700_2136|167|1330|1496|PF00270.29, 700_2136|176|483|658|PF00270.29, 789_2136|145|714|858|PF00271.31, 2136|110|254|363|PF18149.1, 2136|304|982|1285|PF02889.16, 2136|312|1812|2123|PF02889.16, 2136|253|1|253|UNK1, 2136|119|364|482|UNK2, 2136|55|659|713|UNK3, 2136|123|859|981|UNK4, 2136|44|1286|1329|UNK5, 2136|315|1497|1811|UNK6

Secondary: sp|P24230|RECG_ECOLI ATP-dependent DNA helicase RecG OS=Escherichia coli (strain K12) OX=83333 GN=recG PE=1 SV=1

789_693|108|481|588|PF00271.31, 693|157|15|171|PF17191.4, 693|161|273|433|PF00270.29, 693|101|172|272|UNK1, 693|47|434|480|UNK2, 693|105|589|693|UNK3

Secondary: sp|P30015|LHR_ECOLI Probable ATP-dependent helicase lhr OS=Escherichia coli (strain K12) OX=83333 GN=lhr PE=3 SV=2

700_1538|188|31|218|PF00270.29, 1538|69|349|417|PF00271.31, 1538|189|679|867|PF08494.11, 1538|30|1|30|UNK1, 1538|130|219|348|UNK2, 1538|261|418|678|UNK3, 1538|671|868|1538|UNK4

Secondary: sp|P30958|MFD_ECOLI Transcription-repair-coupling factor OS=Escherichia coli (strain K12) OX=83333 GN=mfd PE=1 SV=2

789_1148|104|803|906|PF00271.31, 1148|89|129|217|PF17757.1, 1148|96|477|572|PF02559.16, 1148|161|602|762|PF00270.29, 1148|93|1006|1098|PF03461.15, 1148|128|1|128|UNK1, 1148|259|218|476|UNK2, 1148|40|763|802|UNK3, 1148|99|907|1005|UNK4, 1148|50|1099|1148|UNK5

Primary: sp|Q9H845|ACAD9_HUMAN Acyl-CoA dehydrogenase family member 9, mitochondrial OS=Homo sapiens OX=9606 GN=ACAD9 PE=1 SV=1

661_621|101|177|277|PF02770.19, 803_621|106|68|173|PF02771.16, 621|148|290|437|PF00441.24, 621|67|1|67|UNK1, 621|184|438|621|UNK2

Secondary: sp|P0A9U8|YDIO_ECOLI Probable acyl-CoA dehydrogenase YdiO OS=Escherichia coli (strain K12) OX=83333 GN=ydiO PE=3 SV=1

427_383|149|228|376|PF00441.24, 661_383|96|121|216|PF02770.19, 889_383|102|6|107|PF02771.16

Secondary: sp|P60584|CAIA_ECOLI Crotonobetainyl-CoA reductase OS=Escherichia coli (strain K12) OX=83333 GN=caiA PE=1 SV=1

427_380|150|227|376|PF00441.24, 661_380|93|123|215|PF02770.19, 889_380|103|6|108|PF02771.16

Secondary: sp|Q47146|FADE_ECOLI Acyl-coenzyme A dehydrogenase OS=Escherichia coli (strain K12) OX=83333 GN=fadE PE=2 SV=2

803_814|86|147|232|PF02771.16, 814|92|237|328|PF02770.19, 814|145|360|504|PF00441.24, 814|279|514|792|PF09317.11, 814|146|1|146|UNK1, 814|31|329|359|UNK2

Primary: sp|P76081|PAAE_ECOLI 1,2-phenylacetyl-CoA epoxidase, subunit E OS=Escherichia coli (strain K12) OX=83333 GN=paaE PE=1 SV=1

808_356|109|118|226|PF00175.21, 844_356|99|7|105|PF00970.24, 356|75|267|341|PF00111.27, 356|40|227|266|UNK1

Secondary: sp|Q6IPT4|NB5R5_HUMAN NADH-cytochrome b5 reductase-like OS=Homo sapiens OX=9606 GN=CYB5RL PE=2 SV=3

844_315|94|83|176|PF00970.24, 315|37|18|54|PF09791.9, 315|113|186|298|PF00175.21

Secondary: sp|Q7L1T6|NB5R4_HUMAN Cytochrome b5 reductase 4 OS=Homo sapiens OX=9606 GN=CYB5R4 PE=1 SV=1

808_521|106|395|500|PF00175.21, 521|72|58|129|PF00173.28, 521|79|168|246|PF04969.16, 521|107|277|383|PF00970.24, 521|57|1|57|UNK1, 521|38|130|167|UNK2, 521|30|247|276|UNK3

Primary: sp|P0AFG3|ODO1_ECOLI 2-oxoglutarate dehydrogenase E1 component OS=Escherichia coli (strain K12) OX=83333 GN=sucA PE=1 SV=1

63_933|292|219|510|PF00676.20, 95_933|195|591|785|PF02779.24, 340_933|143|788|930|PF16870.5, 494_933|168|51|218|UNK1, 933|39|12|50|PF16078.5, 933|80|511|590|UNK2

Secondary: sp|Q02218|ODO1_HUMAN 2-oxoglutarate dehydrogenase, mitochondrial OS=Homo sapiens OX=9606 GN=OGDH PE=1 SV=3

63_1023|325|257|581|PF00676.20, 494_1023|170|87|256|UNK2, 1023|39|48|86|PF16078.5, 1023|217|650|866|PF02779.24, 1023|146|869|1014|PF16870.5, 1023|47|1|47|UNK1, 1023|68|582|649|UNK3

Secondary: sp|Q96HY7|DHTK1_HUMAN Probable 2-oxoglutarate dehydrogenase E1 component DHKTD1, mitochondrial OS=Homo sapiens OX=9606 GN=DHTKD1 PE=1 SV=2

95_919|204|568|771|PF02779.24, 340_919|142|777|918|PF16870.5, 919|280|217|496|PF00676.20, 919|216|1|216|UNK1, 919|71|497|567|UNK2

Secondary: sp|Q9ULD0|OGDHL_HUMAN 2-oxoglutarate dehydrogenase-like, mitochondrial OS=Homo sapiens OX=9606 GN=OGDHL PE=1 SV=3

63_1010|325|244|568|PF00676.20, 494_1010|163|81|243|UNK2, 1010|35|46|80|PF16078.5, 1010|216|637|852|PF02779.24, 1010|146|855|1000|PF16870.5, 1010|45|1|45|UNK1, 1010|68|569|636|UNK3

Primary: sp|A1L0T0|ILVBL_HUMAN Acetolactate synthase-like protein OS=Homo sapiens OX=9606 GN=ILVBL PE=1 SV=2

360_632|161|53|213|PF02776.18, 668_632|152|467|618|PF02775.21, 632|132|273|404|PF00205.22, 632|52|1|52|UNK1, 632|59|214|272|UNK2, 632|62|405|466|UNK3

Secondary: sp|P07003|POXB_ECOLI Pyruvate dehydrogenase [ubiquinone] OS=Escherichia coli (strain K12) OX=83333 GN=poxB PE=1 SV=1

668_572|147|379|525|PF02775.21, 572|167|4|170|PF02776.18, 572|128|192|319|PF00205.22, 572|59|320|378|UNK1, 572|47|526|572|UNK2

Secondary: sp|P0DP89|ILVGP_ECOLI Putative acetolactate synthase isozyme 2 large subunit OS=Escherichia coli (strain K12) OX=83333 GN=ilvG PE=5 SV=1

360_327|166|1|166|PF02776.18, 327|136|186|321|PF00205.22

Primary: sp|P24186|FOLD_ECOLI Bifunctional protein FolD OS=Escherichia coli (strain K12) OX=83333 GN=folD PE=1 SV=4

241_288|156|124|279|PF02882.19, 351_288|117|5|121|PF00763.23

Secondary: sp|P11586|C1TC_HUMAN C-1-tetrahydrofolate synthase, cytoplasmic OS=Homo sapiens OX=9606 GN=MTHFD1 PE=1 SV=3

241_935|165|129|293|PF02882.19, 935|120|6|125|PF00763.23, 935|618|318|935|PF01268.19

Secondary: sp|P13995|MTDC_HUMAN Bifunctional methylenetetrahydrofolate dehydrogenase/cyclohydrolase, mitochondrial OS=Homo sapiens OX=9606 GN=MTHFD2 PE=1 SV=2

351_350|116|40|155|PF00763.23, 350|173|158|330|PF02882.19, 350|39|1|39|UNK1

Secondary: sp|Q9H903|MTD2L_HUMAN Probable bifunctional methylenetetrahydrofolate dehydrogenase/cyclohydrolase 2 OS=Homo sapiens OX=9606 GN=MTHFD2L PE=2 SV=3

351_347|117|53|169|PF00763.23, 347|173|172|344|PF02882.19, 347|52|1|52|UNK1

Primary: sp|P38038|CYSJ_ECOLI Sulfite reductase [NADPH] flavoprotein alpha-component OS=Escherichia coli (strain K12) OX=83333 GN=cysJ PE=1 SV=4

359_599|107|457|563|PF00175.21, 589_599|131|66|196|PF00258.25, 598_599|195|230|424|PF00667.20, 599|65|1|65|UNK1, 599|33|197|229|UNK2, 599|32|425|456|UNK3, 599|36|564|599|UNK4

Secondary: sp|P29474|NOS3_HUMAN Nitric oxide synthase, endothelial OS=Homo sapiens OX=9606 GN=NOS3 PE=1 SV=4

589_1203|177|522|698|PF00258.25, 598_1203|228|752|979|PF00667.20, 1203|361|121|481|PF02898.15, 1203|113|1011|1123|PF00175.21, 1203|120|1|120|UNK1, 1203|40|482|521|UNK2, 1203|53|699|751|UNK3, 1203|31|980|1010|UNK4, 1203|80|1124|1203|UNK5

Secondary: sp|P29475|NOS1_HUMAN Nitric oxide synthase, brain OS=Homo sapiens OX=9606 GN=NOS1 PE=1 SV=2

589_1434|174|762|935|PF00258.25, 598_1434|229|991|1219|PF00667.20, 1434|77|18|94|PF00595.24, 1434|361|357|717|PF02898.15, 1434|113|1251|1363|PF00175.21, 1434|262|95|356|UNK1, 1434|44|718|761|UNK2, 1434|55|936|990|UNK3, 1434|31|1220|1250|UNK4, 1434|71|1364|1434|UNK5

Secondary: sp|P35228|NOS2_HUMAN Nitric oxide synthase, inducible OS=Homo sapiens OX=9606 GN=NOS2 PE=1 SV=2

589_1153|132|541|672|PF00258.25, 598_1153|222|726|947|PF00667.20, 1153|361|137|497|PF02898.15, 1153|114|979|1092|PF00175.21, 1153|136|1|136|UNK1, 1153|43|498|540|UNK2, 1153|53|673|725|UNK3, 1153|31|948|978|UNK4, 1153|61|1093|1153|UNK5

Secondary: sp|Q9UBK8|MTRR_HUMAN Methionine synthase reductase OS=Homo sapiens OX=9606 GN=MTRR PE=1 SV=3

598_725|222|298|519|PF00667.20, 649_725|137|33|169|PF00258.25, 725|120|569|688|PF00175.21, 725|32|1|32|UNK1, 725|128|170|297|UNK2, 725|49|520|568|UNK3, 725|37|689|725|UNK4

Secondary: sp|Q9UHB4|NDOR1_HUMAN NADPH-dependent diflavin oxidoreductase 1 OS=Homo sapiens OX=9606 GN=NDOR1 PE=1 SV=1

589_597|138|8|145|PF00258.25, 598_597|219|203|421|PF00667.20, 597|106|455|560|PF00175.21, 597|57|146|202|UNK1, 597|33|422|454|UNK2, 597|37|561|597|UNK3

Primary: sp|P15043|RECQ_ECOLI ATP-dependent DNA helicase RecQ OS=Escherichia coli (strain K12) OX=83333 GN=recQ PE=1 SV=5

232_609|160|28|187|PF00270.29, 349_609|109|222|330|PF00271.31, 836_609|109|406|514|PF09382.10, 609|62|343|404|PF16124.5, 609|67|533|599|PF00570.23, 609|34|188|221|UNK1

Secondary: sp|O94762|RECQ5_HUMAN ATP-dependent DNA helicase Q5 OS=Homo sapiens OX=9606 GN=RECQL5 PE=1 SV=2

232_991|170|31|200|PF00270.29, 991|95|259|353|PF00271.31, 991|70|366|435|PF16124.5, 991|203|625|827|PF06959.11, 991|30|1|30|UNK1, 991|58|201|258|UNK2, 991|189|436|624|UNK3, 991|164|828|991|UNK4

Secondary: sp|P46063|RECQ1_HUMAN ATP-dependent DNA helicase Q1 OS=Homo sapiens OX=9606 GN=RECQL PE=1 SV=3

232_649|167|94|260|PF00270.29, 649|107|301|407|PF00271.31, 649|59|421|479|PF16124.5, 649|93|1|93|UNK1, 649|40|261|300|UNK2, 649|170|480|649|UNK3

Secondary: sp|P54132|BLM_HUMAN Bloom syndrome protein OS=Homo sapiens OX=9606 GN=BLM PE=1 SV=1

232_1417|168|671|838|PF00270.29, 349_1417|101|883|983|PF00271.31, 1417|367|1|367|PF16202.5, 1417|40|372|411|PF08072.11, 1417|223|425|647|PF16204.5, 1417|71|996|1066|PF16124.5, 1417|124|1072|1195|PF09382.10, 1417|65|1217|1281|PF00570.23, 1417|44|839|882|UNK1, 1417|136|1282|1417|UNK2

Secondary: sp|Q14191|WRN_HUMAN Werner syndrome ATP-dependent helicase OS=Homo sapiens OX=9606 GN=WRN PE=1 SV=2

836_1432|99|955|1053|PF09382.10, 1432|169|60|228|PF01612.20, 1432|160|551|710|PF00270.29, 1432|96|763|858|PF00271.31, 1432|70|871|940|PF16124.5, 1432|58|1156|1213|PF00570.23, 1432|94|1259|1352|PF14493.6, 1432|59|1|59|UNK1, 1432|322|229|550|UNK2, 1432|52|711|762|UNK3, 1432|102|1054|1155|UNK4, 1432|45|1214|1258|UNK5, 1432|80|1353|1432|UNK6

Primary: sp|Q9UIF7|MUTYH_HUMAN Adenine DNA glycosylase OS=Homo sapiens OX=9606 GN=MUTYH PE=1 SV=1

286_546|134|129|262|PF00730.25, 882_546|110|382|491|PF14815.6, 546|128|1|128|UNK1, 546|119|263|381|UNK2, 546|55|492|546|UNK3

Secondary: sp|P08337|MUTT_ECOLI 8-oxo-dGTP diphosphatase OS=Escherichia coli (strain K12) OX=83333 GN=mutT PE=1 SV=1

882_129|121|5|125|PF00293.28

Secondary: sp|P17802|MUTY_ECOLI Adenine DNA glycosylase OS=Escherichia coli (strain K12) OX=83333 GN=mutY PE=1 SV=1

286_350|133|36|168|PF00730.25, 350|17|192|208|PF10576.9, 350|110|234|343|PF14815.6, 350|35|1|35|UNK1

Secondary: sp|P77788|NUDG_ECOLI CTP pyrophosphohydrolase OS=Escherichia coli (strain K12) OX=83333 GN=nudG PE=1 SV=1

882_135|121|5|125|PF00293.28

Primary: sp|Q86UK0|ABCAC_HUMAN ATP-binding cassette sub-family A member 12 OS=Homo sapiens OX=9606 GN=ABCA12 PE=1 SV=3

443_2595|146|1361|1506|PF00005.27, 841_2595|178|2418|2595|UNK5, 888_2595|237|1507|1743|UNK3, 2595|337|934|1270|PF12698.7, 2595|424|1744|2167|PF12698.7, 2595|145|2273|2417|PF00005.27, 2595|933|1|933|UNK1, 2595|90|1271|1360|UNK2, 2595|105|2168|2272|UNK4

Secondary: sp|P0A9U1|YBHF_ECOLI Probable multidrug ABC transporter ATP-binding protein YbhF OS=Escherichia coli (strain K12) OX=83333 GN=ybhF PE=1 SV=1

400_578|144|346|489|PF00005.27, 443_578|140|26|165|PF00005.27, 888_578|89|490|578|UNK2, 578|180|166|345|UNK1

Secondary: sp|P36879|YADG_ECOLI Uncharacterized ABC transporter ATP-binding protein YadG OS=Escherichia coli (strain K12) OX=83333 GN=yadG PE=1 SV=1

417_308|145|21|165|PF00005.27, 841_308|143|166|308|UNK1

Secondary: sp|P37624|RBBA_ECOLI Ribosome-associated ATPase OS=Escherichia coli (strain K12) OX=83333 GN=rbbA PE=1 SV=3

400_911|144|293|436|PF00005.27, 443_911|148|28|175|PF00005.27, 888_911|132|437|568|UNK2, 888_911|117|176|292|UNK1, 911|335|569|903|PF12698.7

Primary: sp|Q96RQ3|MCCA_HUMAN Methylcrotonoyl-CoA carboxylase subunit alpha, mitochondrial OS=Homo sapiens OX=9606 GN=MCCC1 PE=1 SV=3

91_725|207|163|369|PF02786.17, 797_725|63|651|713|PF00364.22, 725|109|49|157|PF00289.22, 725|108|383|490|PF02785.19, 725|48|1|48|UNK1, 725|160|491|650|UNK2

Secondary: sp|P0ABD8|BCCP_ECOLI Biotin carboxyl carrier protein of acetyl-CoA carboxylase OS=Escherichia coli (strain K12) OX=83333 GN=accB PE=1 SV=1

797_156|73|83|155|PF00364.22, 156|82|1|82|UNK1

Secondary: sp|P24182|ACCC_ECOLI Biotin carboxylase OS=Escherichia coli (strain K12) OX=83333 GN=accC PE=1 SV=2

91_449|209|115|323|PF02786.17, 265_449|110|1|110|PF00289.22, 562_449|106|336|441|PF02785.19

Primary: sp|Q86UQ4|ABCAD_HUMAN ATP-binding cassette sub-family A member 13 OS=Homo sapiens OX=9606 GN=ABCA13 PE=2 SV=3

443_5058|146|3859|4004|PF00005.27, 841_5058|174|4885|5058|UNK5, 5058|224|3547|3770|PF12698.7, 5058|265|4390|4654|PF12698.7, 5058|147|4738|4884|PF00005.27, 5058|3546|1|3546|UNK1, 5058|88|3771|3858|UNK2, 5058|385|4005|4389|UNK3, 5058|83|4655|4737|UNK4

Secondary: sp|P0A9U1|YBHF_ECOLI Probable multidrug ABC transporter ATP-binding protein YbhF OS=Escherichia coli (strain K12) OX=83333 GN=ybhF PE=1 SV=1

400_578|144|346|489|PF00005.27, 443_578|140|26|165|PF00005.27, 888_578|89|490|578|UNK2, 578|180|166|345|UNK1

Secondary: sp|P36879|YADG_ECOLI Uncharacterized ABC transporter ATP-binding protein YadG OS=Escherichia coli (strain K12) OX=83333 GN=yadG PE=1 SV=1

417_308|145|21|165|PF00005.27, 841_308|143|166|308|UNK1

Secondary: sp|P37624|RBBA_ECOLI Ribosome-associated ATPase OS=Escherichia coli (strain K12) OX=83333 GN=rbbA PE=1 SV=3

400_911|144|293|436|PF00005.27, 443_911|148|28|175|PF00005.27, 888_911|132|437|568|UNK2, 888_911|117|176|292|UNK1, 911|335|569|903|PF12698.7

Primary: sp|P24182|ACCC_ECOLI Biotin carboxylase OS=Escherichia coli (strain K12) OX=83333 GN=accC PE=1 SV=2

91_449|209|115|323|PF02786.17, 265_449|110|1|110|PF00289.22, 562_449|106|336|441|PF02785.19

Secondary: sp|P05165|PCCA_HUMAN Propionyl-CoA carboxylase alpha chain, mitochondrial OS=Homo sapiens OX=9606 GN=PCCA PE=1 SV=4

265_728|109|63|171|PF00289.22, 728|209|176|384|PF02786.17, 728|109|397|505|PF02785.19, 728|129|525|653|PF18140.1, 728|66|662|727|PF00364.22, 728|62|1|62|UNK1

Secondary: sp|P11498|PYC_HUMAN Pyruvate carboxylase, mitochondrial OS=Homo sapiens OX=9606 GN=PC PE=1 SV=2

562_1178|109|375|483|PF02785.19, 1178|109|37|145|PF00289.22, 1178|208|151|358|PF02786.17, 1178|271|565|835|PF00682.19, 1178|201|861|1061|PF02436.18, 1178|67|1111|1177|PF00364.22, 1178|36|1|36|UNK1, 1178|81|484|564|UNK2, 1178|49|1062|1110|UNK3

Secondary: sp|Q96RQ3|MCCA_HUMAN Methylcrotonoyl-CoA carboxylase subunit alpha, mitochondrial OS=Homo sapiens OX=9606 GN=MCCC1 PE=1 SV=3

91_725|207|163|369|PF02786.17, 797_725|63|651|713|PF00364.22, 725|109|49|157|PF00289.22, 725|108|383|490|PF02785.19, 725|48|1|48|UNK1, 725|160|491|650|UNK2

Primary: sp|Q3SY69|AL1L2_HUMAN Mitochondrial 10-formyltetrahydrofolate dehydrogenase OS=Homo sapiens OX=9606 GN=ALDH1L2 PE=1 SV=2

22_923|468|452|919|PF00171.22, 431_923|179|24|202|PF00551.19, 923|102|227|328|PF02911.18, 923|54|347|400|PF00550.25, 923|51|401|451|UNK1

Secondary: sp|P23882|FMT_ECOLI Methionyl-tRNA formyltransferase OS=Escherichia coli (strain K12) OX=83333 GN=fmt PE=1 SV=4

431_315|178|6|183|PF00551.19, 315|98|207|304|PF02911.18

Secondary: sp|P23883|PUUC_ECOLI NADP/NAD-dependent aldehyde dehydrogenase PuuC OS=Escherichia coli (strain K12) OX=83333 GN=puuC PE=1 SV=2

22_495|458|32|489|PF00171.22, 495|31|1|31|UNK1

Primary: sp|P49748|ACADV_HUMAN Very long-chain specific acyl-CoA dehydrogenase, mitochondrial OS=Homo sapiens OX=9606 GN=ACADVL PE=1 SV=1

661_655|103|213|315|PF02770.19, 803_655|114|96|209|PF02771.16, 655|147|327|473|PF00441.24, 655|95|1|95|UNK1, 655|182|474|655|UNK2

Secondary: sp|P0A9U8|YDIO_ECOLI Probable acyl-CoA dehydrogenase YdiO OS=Escherichia coli (strain K12) OX=83333 GN=ydiO PE=3 SV=1

427_383|149|228|376|PF00441.24, 661_383|96|121|216|PF02770.19, 889_383|102|6|107|PF02771.16

Secondary: sp|P60584|CAIA_ECOLI Crotonobetainyl-CoA reductase OS=Escherichia coli (strain K12) OX=83333 GN=caiA PE=1 SV=1

427_380|150|227|376|PF00441.24, 661_380|93|123|215|PF02770.19, 889_380|103|6|108|PF02771.16

Secondary: sp|Q47146|FADE_ECOLI Acyl-coenzyme A dehydrogenase OS=Escherichia coli (strain K12) OX=83333 GN=fadE PE=2 SV=2

803_814|86|147|232|PF02771.16, 814|92|237|328|PF02770.19, 814|145|360|504|PF00441.24, 814|279|514|792|PF09317.11, 814|146|1|146|UNK1, 814|31|329|359|UNK2

Primary: sp|P0AAI3|FTSH_ECOLI ATP-dependent zinc metalloprotease FtsH OS=Escherichia coli (strain K12) OX=83333 GN=ftsH PE=1 SV=1

128_644|133|188|320|PF00004.29, 246_644|191|401|591|PF01434.18, 580_644|94|94|187|UNK1, 771_644|45|343|387|PF17862.1, 644|89|5|93|PF06480.15, 644|53|592|644|UNK2

Secondary: sp|Q96TA2|YMEL1_HUMAN ATP-dependent zinc metalloprotease YME1L1 OS=Homo sapiens OX=9606 GN=YME1L1 PE=1 SV=2

246_773|175|588|762|PF01434.18, 771_773|44|528|571|PF17862.1, 773|132|375|506|PF00004.29, 773|374|1|374|UNK1

Secondary: sp|Q9Y4W6|AFG32_HUMAN AFG3-like protein 2 OS=Homo sapiens OX=9606 GN=AFG3L2 PE=1 SV=2

128_797|132|345|476|PF00004.29, 580_797|104|241|344|UNK2, 797|91|150|240|PF06480.15, 797|39|508|546|PF17862.1, 797|182|562|743|PF01434.18, 797|149|1|149|UNK1, 797|31|477|507|UNK3, 797|54|744|797|UNK4

Primary: sp|Q02880|TOP2B_HUMAN DNA topoisomerase 2-beta OS=Homo sapiens OX=9606 GN=TOP2B PE=1 SV=3

508_1626|456|734|1189|PF00521.20, 601_1626|102|478|579|PF01751.22, 800_1626|146|100|245|PF02518.26, 1626|161|287|447|PF00204.25, 1626|139|594|732|PF16898.5, 1626|103|1508|1610|PF08070.11, 1626|99|1|99|UNK1, 1626|41|246|286|UNK2, 1626|30|448|477|UNK3, 1626|318|1190|1507|UNK4

Secondary: sp|P0AES4|GYRA_ECOLI DNA gyrase subunit A OS=Escherichia coli (strain K12) OX=83333 GN=gyrA PE=1 SV=1

508_875|475|32|506|PF00521.20, 875|31|1|31|UNK1, 875|31|507|537|UNK2, 875|36|840|875|UNK3

Secondary: sp|P0AES6|GYRB_ECOLI DNA gyrase subunit B OS=Escherichia coli (strain K12) OX=83333 GN=gyrB PE=1 SV=2

601_804|113|419|531|PF01751.22, 800_804|143|32|174|PF02518.26, 804|170|221|390|PF00204.25, 804|166|564|729|PF18053.1, 804|62|732|793|PF00986.21, 804|31|1|31|UNK1, 804|46|175|220|UNK2, 804|32|532|563|UNK3

Secondary: sp|P0AFI2|PARC_ECOLI DNA topoisomerase 4 subunit A OS=Escherichia coli (strain K12) OX=83333 GN=parC PE=1 SV=1

508_752|437|29|465|PF00521.20, 752|127|466|592|UNK1, 752|77|676|752|UNK2

Secondary: sp|P20083|PARE_ECOLI DNA topoisomerase 4 subunit B OS=Escherichia coli (strain K12) OX=83333 GN=parE PE=1 SV=3

601_630|109|413|521|PF01751.22, 800_630|144|28|171|PF02518.26, 630|167|218|384|PF00204.25, 630|65|556|620|PF00986.21, 630|46|172|217|UNK1, 630|34|522|555|UNK2

Primary: sp|P15034|AMPP_ECOLI Xaa-Pro aminopeptidase OS=Escherichia coli (strain K12) OX=83333 GN=pepP PE=1 SV=2

176_441|230|185|414|PF00557.24, 734_441|121|9|129|PF05195.16, 441|55|130|184|UNK1

Secondary: sp|P12955|PEPD_HUMAN Xaa-Pro dipeptidase OS=Homo sapiens OX=9606 GN=PEPD PE=1 SV=3

176_493|265|195|459|PF00557.24, 493|117|23|139|PF05195.16, 493|55|140|194|UNK1, 493|34|460|493|UNK2

Secondary: sp|Q9NQH7|XPP3_HUMAN Probable Xaa-Pro aminopeptidase 3 OS=Homo sapiens OX=9606 GN=XPNPEP3 PE=1 SV=1

734_507|128|72|199|PF05195.16, 507|230|253|482|PF00557.24, 507|71|1|71|UNK1, 507|53|200|252|UNK2

Primary: sp|Q9Y223|GLCNE_HUMAN Bifunctional UDP-N-acetylglucosamine 2-epimerase/N-acetylmannosamine kinase OS=Homo sapiens OX=9606 GN=GNE PE=1 SV=1

433_722|305|410|714|PF00480.20, 643_722|338|38|375|PF02350.19, 722|37|1|37|UNK1, 722|34|376|409|UNK2

Secondary: sp|P0AF20|NAGC_ECOLI N-acetylglucosamine repressor OS=Escherichia coli (strain K12) OX=83333 GN=nagC PE=2 SV=1

433_406|304|89|392|PF00480.20, 406|39|25|63|PF01047.22

Secondary: sp|P27828|WECB_ECOLI UDP-N-acetylglucosamine 2-epimerase OS=Escherichia coli (strain K12) OX=83333 GN=wecB PE=1 SV=2

643_376|349|22|370|PF02350.19

Secondary: sp|P50456|MLC_ECOLI Protein mlc OS=Escherichia coli (strain K12) OX=83333 GN=mlc PE=1 SV=2

433_406|294|96|389|PF00480.20, 406|95|1|95|UNK1

Primary: sp|Q14562|DHX8_HUMAN ATP-dependent RNA helicase DHX8 OS=Homo sapiens OX=9606 GN=DHX8 PE=1 SV=1

381_1220|129|767|895|PF00271.31, 687_1220|89|957|1045|PF04408.23, 755_1220|74|261|334|PF00575.23, 862_1220|77|1103|1179|PF07717.16, 1220|260|1|260|UNK1, 1220|432|335|766|UNK2, 1220|61|896|956|UNK3, 1220|57|1046|1102|UNK4, 1220|41|1180|1220|UNK5

Secondary: sp|P05055|PNP_ECOLI Polyribonucleotide nucleotidyltransferase OS=Escherichia coli (strain K12) OX=83333 GN=pnp PE=1 SV=3

293_711|133|324|456|PF01138.21, 354_711|129|16|144|PF01138.21, 567_711|68|460|527|PF03725.15, 749_711|64|147|210|PF03725.15, 755_711|72|619|690|PF00575.23, 711|80|241|320|PF03726.14, 711|59|556|614|PF00013.29, 711|30|211|240|UNK1

Secondary: sp|P43329|HRPA_ECOLI ATP-dependent RNA helicase HrpA OS=Escherichia coli (strain K12) OX=83333 GN=hrpA PE=3 SV=3

194_1300|149|85|233|PF00270.29, 381_1300|119|285|403|PF00271.31, 687_1300|86|466|551|PF04408.23, 719_1300|62|404|465|UNK3, 862_1300|77|622|698|PF07717.16, 1300|585|713|1297|PF11898.8, 1300|84|1|84|UNK1, 1300|51|234|284|UNK2, 1300|70|552|621|UNK4

Primary: sp|O00764|PDXK_HUMAN Pyridoxal kinase OS=Homo sapiens OX=9606 GN=PDXK PE=1 SV=1

594_312|93|1|93|UNK1, 619_312|182|94|275|PF08543.12, 312|37|276|312|UNK2

Secondary: sp|P40191|PDXK_ECOLI Pyridoxine/pyridoxal/pyridoxamine kinase OS=Escherichia coli (strain K12) OX=83333 GN=pdxK PE=1 SV=2

619_283|178|88|265|PF08543.12, 283|87|1|87|UNK1

Secondary: sp|P77150|PDXY_ECOLI Pyridoxal kinase PdxY OS=Escherichia coli (strain K12) OX=83333 GN=pdxY PE=1 SV=3

594_287|67|1|67|UNK1, 287|189|68|256|PF08543.12, 287|31|257|287|UNK2

Primary: sp|P0A9M0|LON_ECOLI Lon protease OS=Escherichia coli (strain K12) OX=83333 GN=lon PE=1 SV=1

139_784|138|352|489|PF00004.29, 156_784|203|570|772|PF05362.13, 348_784|150|202|351|UNK1, 595_784|80|490|569|UNK2, 784|192|10|201|PF02190.16

Secondary: sp|P36776|LONM_HUMAN Lon protease homolog, mitochondrial OS=Homo sapiens OX=9606 GN=LONP1 PE=1 SV=2

348_959|150|369|518|UNK2, 959|245|124|368|PF02190.16, 959|138|519|656|PF00004.29, 959|211|737|947|PF05362.13, 959|123|1|123|UNK1, 959|80|657|736|UNK3

Secondary: sp|Q86WA8|LONP2_HUMAN Lon protease homolog 2, peroxisomal OS=Homo sapiens OX=9606 GN=LONP2 PE=1 SV=1

139_852|138|371|508|PF00004.29, 156_852|207|629|835|PF05362.13, 595_852|120|509|628|UNK2, 852|207|13|219|PF02190.16, 852|151|220|370|UNK1

Primary: sp|O75891|AL1L1_HUMAN Cytosolic 10-formyltetrahydrofolate dehydrogenase OS=Homo sapiens OX=9606 GN=ALDH1L1 PE=1 SV=2

22_902|469|430|898|PF00171.22, 431_902|180|1|180|PF00551.19, 902|105|205|309|PF02911.18, 902|67|325|391|PF00550.25, 902|38|392|429|UNK1

Secondary: sp|P23882|FMT_ECOLI Methionyl-tRNA formyltransferase OS=Escherichia coli (strain K12) OX=83333 GN=fmt PE=1 SV=4

431_315|178|6|183|PF00551.19, 315|98|207|304|PF02911.18

Secondary: sp|P23883|PUUC_ECOLI NADP/NAD-dependent aldehyde dehydrogenase PuuC OS=Escherichia coli (strain K12) OX=83333 GN=puuC PE=1 SV=2

22_495|458|32|489|PF00171.22, 495|31|1|31|UNK1

Primary: sp|Q9NZB8|MOCS1_HUMAN Molybdenum cofactor biosynthesis protein 1 OS=Homo sapiens OX=9606 GN=MOCS1 PE=1 SV=3

277_636|136|493|628|PF01967.21, 289_636|160|75|234|PF04055.21, 573_636|126|242|367|PF06463.13, 636|74|1|74|UNK1, 636|125|368|492|UNK2

Secondary: sp|P0A738|MOAC_ECOLI Cyclic pyranopterin monophosphate synthase OS=Escherichia coli (strain K12) OX=83333 GN=moaC PE=1 SV=2

277_161|136|15|150|PF01967.21

Secondary: sp|P30745|MOAA_ECOLI GTP 3',8-cyclase OS=Escherichia coli (strain K12) OX=83333 GN=moaA PE=1 SV=1

289_329|163|19|181|PF04055.21, 573_329|127|186|312|PF06463.13

Primary: sp|P47989|XDH_HUMAN Xanthine dehydrogenase/oxidase OS=Homo sapiens OX=9606 GN=XDH PE=1 SV=4

217_1333|533|705|1237|PF02738.18, 652_1333|73|87|159|PF01799.20, 737_1333|178|234|411|PF00941.21, 801_1333|107|588|694|PF01315.22, 1333|69|9|77|PF00111.27, 1333|105|419|523|PF03450.17, 1333|74|160|233|UNK1, 1333|64|524|587|UNK2, 1333|96|1238|1333|UNK3

Secondary: sp|Q46799|XDHA_ECOLI Putative xanthine dehydrogenase molybdenum-binding subunit XdhA OS=Escherichia coli (strain K12) OX=83333 GN=xdhA PE=2 SV=1

217_752|543|145|687|PF02738.18, 801_752|117|10|126|PF01315.22, 752|65|688|752|UNK1

Secondary: sp|Q46800|XDHB_ECOLI Putative xanthine dehydrogenase FAD-binding subunit XdhB OS=Escherichia coli (strain K12) OX=83333 GN=xdhB PE=4 SV=1

737_292|168|6|173|PF00941.21, 292|101|183|283|PF03450.17

Secondary: sp|Q46801|XDHC_ECOLI Putative xanthine dehydrogenase iron-sulfur-binding subunit XdhC OS=Escherichia coli (strain K12) OX=83333 GN=xdhC PE=3 SV=1

652_159|75|78|152|PF01799.20, 159|53|12|64|PF00111.27

Secondary: sp|Q46814|XDHD_ECOLI Probable hypoxanthine oxidase XdhD OS=Escherichia coli (strain K12) OX=83333 GN=xdhD PE=3 SV=1

217_956|539|355|893|PF02738.18, 801_956|111|187|297|PF01315.22, 956|72|73|144|PF01799.20, 956|72|1|72|UNK1, 956|42|145|186|UNK2, 956|57|298|354|UNK3, 956|63|894|956|UNK4

Primary: sp|P76113|CURA_ECOLI NADPH-dependent curcumin reductase OS=Escherichia coli (strain K12) OX=83333 GN=curA PE=1 SV=3

426_345|139|161|299|PF00107.26, 541_345|107|9|115|PF16884.5, 777_345|46|300|345|UNK2, 837_345|45|116|160|UNK1

Secondary: sp|Q14914|PTGR1_HUMAN Prostaglandin reductase 1 OS=Homo sapiens OX=9606 GN=PTGR1 PE=1 SV=2

777_329|45|285|329|UNK2, 837_329|47|106|152|UNK1, 329|101|5|105|PF16884.5, 329|132|153|284|PF00107.26

Secondary: sp|Q8N8N7|PTGR2_HUMAN Prostaglandin reductase 2 OS=Homo sapiens OX=9606 GN=PTGR2 PE=1 SV=1

426_351|102|168|269|PF00107.26, 541_351|113|5|117|PF16884.5, 351|50|118|167|UNK1, 351|82|270|351|UNK2

Secondary: tr|G3V3Y1|G3V3Y1_HUMAN Uncharacterized protein OS=Homo sapiens OX=9606 PE=4 SV=1

541_287|113|5|117|PF16884.5, 287|170|118|287|UNK1

Primary: sp|P0AFG6|ODO2_ECOLI Dihydrolipoyllysine-residue succinyltransferase component of 2-oxoglutarate dehydrogenase complex OS=Escherichia coli (strain K12) OX=83333 GN=sucB PE=1 SV=2

62_405|229|175|403|PF00198.23, 729_405|72|5|76|PF00364.22, 405|34|114|147|PF02817.17, 405|37|77|113|UNK1

Secondary: sp|O00330|ODPX_HUMAN Pyruvate dehydrogenase protein X component, mitochondrial OS=Homo sapiens OX=9606 GN=PDHX PE=1 SV=3

729_501|72|59|130|PF00364.22, 501|35|183|217|PF02817.17, 501|227|274|500|PF00198.23, 501|58|1|58|UNK1, 501|52|131|182|UNK2, 501|56|218|273|UNK3

Secondary: sp|P10515|ODP2_HUMAN Dihydrolipoyllysine-residue acetyltransferase component of pyruvate dehydrogenase complex, mitochondrial OS=Homo sapiens OX=9606 GN=DLAT PE=1 SV=3

729_647|74|220|293|PF00364.22, 729_647|72|93|164|PF00364.22, 647|35|355|389|PF02817.17, 647|228|420|647|PF00198.23, 647|92|1|92|UNK1, 647|55|165|219|UNK2, 647|61|294|354|UNK3, 647|30|390|419|UNK4

Secondary: sp|P36957|ODO2_HUMAN Dihydrolipoyllysine-residue succinyltransferase component of 2-oxoglutarate dehydrogenase complex, mitochondrial OS=Homo sapiens OX=9606 GN=DLST PE=1 SV=4

62_453|229|222|450|PF00198.23, 453|72|72|143|PF00364.22, 453|71|1|71|UNK1, 453|78|144|221|UNK2

Primary: sp|Q9NUT2|ABCB8_HUMAN ATP-binding cassette sub-family B member 8, mitochondrial OS=Homo sapiens OX=9606 GN=ABCB8 PE=1 SV=3

213_735|151|490|640|PF00005.27, 462_735|276|149|424|PF00664.23, 735|148|1|148|UNK1, 735|65|425|489|UNK2, 735|95|641|735|UNK3

Secondary: sp|P0AAG5|MDLB_ECOLI Multidrug resistance-like ATP-binding protein MdlB OS=Escherichia coli (strain K12) OX=83333 GN=mdlB PE=1 SV=1

462_593|270|26|295|PF00664.23, 593|149|357|505|PF00005.27, 593|61|296|356|UNK1, 593|88|506|593|UNK2

Secondary: sp|P60752|MSBA_ECOLI Lipid A export ATP-binding/permease protein MsbA OS=Escherichia coli (strain K12) OX=83333 GN=msbA PE=1 SV=1

213_582|151|359|509|PF00005.27, 468_582|73|510|582|UNK2, 582|271|27|297|PF00664.23, 582|61|298|358|UNK1

Primary: sp|Q5T013|HYI_HUMAN Putative hydroxypyruvate isomerase OS=Homo sapiens OX=9606 GN=HYI PE=1 SV=2

256_277|196|25|220|PF01261.24, 733_277|57|221|277|UNK1

Secondary: sp|P30147|HYI_ECOLI Hydroxypyruvate isomerase OS=Escherichia coli (strain K12) OX=83333 GN=hyi PE=1 SV=1

256_258|199|21|219|PF01261.24, 258|39|220|258|UNK1

Secondary: sp|Q46891|OTNI_ECOLI 2-oxo-tetronate isomerase OS=Escherichia coli (strain K12) OX=83333 GN=otnI PE=1 SV=1

733_258|39|220|258|UNK1, 258|199|21|219|PF01261.24

Primary: sp|P31937|3HIDH_HUMAN 3-hydroxyisobutyrate dehydrogenase, mitochondrial OS=Homo sapiens OX=9606 GN=HIBADH PE=1 SV=2

282_336|159|42|200|PF03446.15, 704_336|128|203|330|PF14833.6, 336|41|1|41|UNK1

Secondary: sp|P0A9V8|SQUU_ECOLI 3-sulfolactaldehyde reductase OS=Escherichia coli (strain K12) OX=83333 GN=yihU PE=1 SV=1

704_298|120|165|284|PF14833.6, 298|157|4|160|PF03446.15

Secondary: sp|P0ABQ2|GARR_ECOLI 2-hydroxy-3-oxopropionate reductase OS=Escherichia coli (strain K12) OX=83333 GN=garR PE=1 SV=1

282_294|160|2|161|PF03446.15, 813_294|118|164|281|PF14833.6

Secondary: sp|Q46888|LTND_ECOLI L-threonate dehydrogenase OS=Escherichia coli (strain K12) OX=83333 GN=ltnD PE=3 SV=1

704_302|120|172|291|PF14833.6, 302|158|9|166|PF03446.15

Primary: sp|Q99758|ABCA3_HUMAN ATP-binding cassette sub-family A member 3 OS=Homo sapiens OX=9606 GN=ABCA3 PE=1 SV=2

417_1704|144|550|693|PF00005.27, 443_1704|143|1400|1542|PF00005.27, 841_1704|162|1543|1704|UNK5, 888_1704|229|694|922|UNK3, 1704|218|252|469|PF12698.7, 1704|400|923|1322|PF12698.7, 1704|251|1|251|UNK1, 1704|80|470|549|UNK2, 1704|77|1323|1399|UNK4

Secondary: sp|P0A9U1|YBHF_ECOLI Probable multidrug ABC transporter ATP-binding protein YbhF OS=Escherichia coli (strain K12) OX=83333 GN=ybhF PE=1 SV=1

400_578|144|346|489|PF00005.27, 443_578|140|26|165|PF00005.27, 888_578|89|490|578|UNK2, 578|180|166|345|UNK1

Secondary: sp|P36879|YADG_ECOLI Uncharacterized ABC transporter ATP-binding protein YadG OS=Escherichia coli (strain K12) OX=83333 GN=yadG PE=1 SV=1

417_308|145|21|165|PF00005.27, 841_308|143|166|308|UNK1

Secondary: sp|P37624|RBBA_ECOLI Ribosome-associated ATPase OS=Escherichia coli (strain K12) OX=83333 GN=rbbA PE=1 SV=3

400_911|144|293|436|PF00005.27, 443_911|148|28|175|PF00005.27, 888_911|132|437|568|UNK2, 888_911|117|176|292|UNK1, 911|335|569|903|PF12698.7

Primary: sp|O95477|ABCA1_HUMAN ATP-binding cassette sub-family A member 1 OS=Homo sapiens OX=9606 GN=ABCA1 PE=1 SV=3

443_2261|145|917|1061|PF00005.27, 841_2261|189|2073|2261|UNK5, 888_2261|285|1062|1346|UNK3, 2261|202|640|841|PF12698.7, 2261|522|1347|1868|PF12698.7, 2261|143|1930|2072|PF00005.27, 2261|639|1|639|UNK1, 2261|75|842|916|UNK2, 2261|61|1869|1929|UNK4

Secondary: sp|P0A9U1|YBHF_ECOLI Probable multidrug ABC transporter ATP-binding protein YbhF OS=Escherichia coli (strain K12) OX=83333 GN=ybhF PE=1 SV=1

400_578|144|346|489|PF00005.27, 443_578|140|26|165|PF00005.27, 888_578|89|490|578|UNK2, 578|180|166|345|UNK1

Secondary: sp|P36879|YADG_ECOLI Uncharacterized ABC transporter ATP-binding protein YadG OS=Escherichia coli (strain K12) OX=83333 GN=yadG PE=1 SV=1

417_308|145|21|165|PF00005.27, 841_308|143|166|308|UNK1

Secondary: sp|P37624|RBBA_ECOLI Ribosome-associated ATPase OS=Escherichia coli (strain K12) OX=83333 GN=rbbA PE=1 SV=3

400_911|144|293|436|PF00005.27, 443_911|148|28|175|PF00005.27, 888_911|132|437|568|UNK2, 888_911|117|176|292|UNK1, 911|335|569|903|PF12698.7

Primary: sp|P22102|PUR2_HUMAN Trifunctional purine biosynthetic protein adenosine-3 OS=Homo sapiens OX=9606 GN=GART PE=1 SV=1

115_1010|194|105|298|PF01071.19, 204_1010|180|809|988|PF00551.19, 228_1010|101|492|592|PF00586.24, 229_1010|170|607|776|PF02769.22, 321_1010|101|4|104|PF02844.15, 553_1010|91|334|424|PF02843.16, 858_1010|67|425|491|UNK2, 1010|35|299|333|UNK1, 1010|32|777|808|UNK3

Secondary: sp|P08178|PUR5_ECOLI Phosphoribosylformylglycinamidine cyclo-ligase OS=Escherichia coli (strain K12) OX=83333 GN=purM PE=1 SV=3

228_345|106|59|164|PF00586.24, 229_345|165|177|341|PF02769.22, 858_345|58|1|58|UNK1

Secondary: sp|P08179|PUR3_ECOLI Phosphoribosylglycinamide formyltransferase OS=Escherichia coli (strain K12) OX=83333 GN=purN PE=1 SV=1

204_212|181|1|181|PF00551.19, 212|31|182|212|UNK1

Secondary: sp|P15640|PUR2_ECOLI Phosphoribosylamine--glycine ligase OS=Escherichia coli (strain K12) OX=83333 GN=purD PE=1 SV=2

115_429|194|103|296|PF01071.19, 321_429|102|1|102|PF02844.15, 553_429|91|332|422|PF02843.16, 429|35|297|331|UNK1

Primary: sp|P0A9U1|YBHF_ECOLI Probable multidrug ABC transporter ATP-binding protein YbhF OS=Escherichia coli (strain K12) OX=83333 GN=ybhF PE=1 SV=1

400_578|144|346|489|PF00005.27, 443_578|140|26|165|PF00005.27, 888_578|89|490|578|UNK2, 578|180|166|345|UNK1

Secondary: sp|O94911|ABCA8_HUMAN ATP-binding cassette sub-family A member 8 OS=Homo sapiens OX=9606 GN=ABCA8 PE=1 SV=3

400_1581|139|1267|1405|PF00005.27, 841_1581|176|1406|1581|UNK5, 1581|381|38|418|PF12698.7, 1581|121|499|619|PF00005.27, 1581|240|901|1140|PF12698.7, 1581|37|1|37|UNK1, 1581|80|419|498|UNK2, 1581|281|620|900|UNK3, 1581|126|1141|1266|UNK4

Secondary: sp|O95477|ABCA1_HUMAN ATP-binding cassette sub-family A member 1 OS=Homo sapiens OX=9606 GN=ABCA1 PE=1 SV=3

443_2261|145|917|1061|PF00005.27, 841_2261|189|2073|2261|UNK5, 888_2261|285|1062|1346|UNK3, 2261|202|640|841|PF12698.7, 2261|522|1347|1868|PF12698.7, 2261|143|1930|2072|PF00005.27, 2261|639|1|639|UNK1, 2261|75|842|916|UNK2, 2261|61|1869|1929|UNK4

Secondary: sp|P78363|ABCA4_HUMAN Retinal-specific ATP-binding cassette transporter OS=Homo sapiens OX=9606 GN=ABCA4 PE=1 SV=3

443_2273|144|947|1090|PF00005.27, 443_2273|142|1957|2098|PF00005.27, 841_2273|175|2099|2273|UNK5, 2273|247|610|856|PF12698.7, 2273|292|1604|1895|PF12698.7, 2273|609|1|609|UNK1, 2273|90|857|946|UNK2, 2273|513|1091|1603|UNK3, 2273|61|1896|1956|UNK4

Secondary: sp|Q86UK0|ABCAC_HUMAN ATP-binding cassette sub-family A member 12 OS=Homo sapiens OX=9606 GN=ABCA12 PE=1 SV=3

443_2595|146|1361|1506|PF00005.27, 841_2595|178|2418|2595|UNK5, 888_2595|237|1507|1743|UNK3, 2595|337|934|1270|PF12698.7, 2595|424|1744|2167|PF12698.7, 2595|145|2273|2417|PF00005.27, 2595|933|1|933|UNK1, 2595|90|1271|1360|UNK2, 2595|105|2168|2272|UNK4

Secondary: sp|Q86UQ4|ABCAD_HUMAN ATP-binding cassette sub-family A member 13 OS=Homo sapiens OX=9606 GN=ABCA13 PE=2 SV=3

443_5058|146|3859|4004|PF00005.27, 841_5058|174|4885|5058|UNK5, 5058|224|3547|3770|PF12698.7, 5058|265|4390|4654|PF12698.7, 5058|147|4738|4884|PF00005.27, 5058|3546|1|3546|UNK1, 5058|88|3771|3858|UNK2, 5058|385|4005|4389|UNK3, 5058|83|4655|4737|UNK4

Secondary: sp|Q8IZY2|ABCA7_HUMAN ATP-binding cassette sub-family A member 7 OS=Homo sapiens OX=9606 GN=ABCA7 PE=1 SV=3

443_2146|145|824|968|PF00005.27, 888_2146|193|1954|2146|UNK5, 2146|200|551|750|PF12698.7, 2146|284|1466|1749|PF12698.7, 2146|142|1812|1953|PF00005.27, 2146|550|1|550|UNK1, 2146|73|751|823|UNK2, 2146|497|969|1465|UNK3, 2146|62|1750|1811|UNK4

Secondary: sp|Q8WWZ4|ABCAA_HUMAN ATP-binding cassette sub-family A member 10 OS=Homo sapiens OX=9606 GN=ABCA10 PE=2 SV=3

400_1543|145|1223|1367|PF00005.27, 443_1543|147|410|556|PF00005.27, 841_1543|176|1368|1543|UNK5, 1543|240|90|329|PF12698.7, 1543|195|936|1130|PF12698.7, 1543|89|1|89|UNK1, 1543|80|330|409|UNK2, 1543|379|557|935|UNK3, 1543|92|1131|1222|UNK4

Secondary: sp|Q99758|ABCA3_HUMAN ATP-binding cassette sub-family A member 3 OS=Homo sapiens OX=9606 GN=ABCA3 PE=1 SV=2

417_1704|144|550|693|PF00005.27, 443_1704|143|1400|1542|PF00005.27, 841_1704|162|1543|1704|UNK5, 888_1704|229|694|922|UNK3, 1704|218|252|469|PF12698.7, 1704|400|923|1322|PF12698.7, 1704|251|1|251|UNK1, 1704|80|470|549|UNK2, 1704|77|1323|1399|UNK4

Secondary: sp|Q9BZC7|ABCA2_HUMAN ATP-binding cassette sub-family A member 2 OS=Homo sapiens OX=9606 GN=ABCA2 PE=1 SV=3

443_2435|145|1007|1151|PF00005.27, 443_2435|142|2072|2213|PF00005.27, 841_2435|222|2214|2435|UNK5, 2435|208|705|912|PF12698.7, 2435|283|1725|2007|PF12698.7, 2435|704|1|704|UNK1, 2435|94|913|1006|UNK2, 2435|573|1152|1724|UNK3, 2435|64|2008|2071|UNK4

Primary: sp|Q8IUA7|ABCA9_HUMAN ATP-binding cassette sub-family A member 9 OS=Homo sapiens OX=9606 GN=ABCA9 PE=1 SV=1

400_1624|139|1310|1448|PF00005.27, 443_1624|147|500|646|PF00005.27, 841_1624|176|1449|1624|UNK5, 888_1624|307|647|953|UNK3, 1624|388|32|419|PF12698.7, 1624|266|954|1219|PF12698.7, 1624|31|1|31|UNK1, 1624|80|420|499|UNK2, 1624|90|1220|1309|UNK4

Secondary: sp|P0A9U1|YBHF_ECOLI Probable multidrug ABC transporter ATP-binding protein YbhF OS=Escherichia coli (strain K12) OX=83333 GN=ybhF PE=1 SV=1

400_578|144|346|489|PF00005.27, 443_578|140|26|165|PF00005.27, 888_578|89|490|578|UNK2, 578|180|166|345|UNK1

Secondary: sp|P36879|YADG_ECOLI Uncharacterized ABC transporter ATP-binding protein YadG OS=Escherichia coli (strain K12) OX=83333 GN=yadG PE=1 SV=1

417_308|145|21|165|PF00005.27, 841_308|143|166|308|UNK1

Secondary: sp|P37624|RBBA_ECOLI Ribosome-associated ATPase OS=Escherichia coli (strain K12) OX=83333 GN=rbbA PE=1 SV=3

400_911|144|293|436|PF00005.27, 443_911|148|28|175|PF00005.27, 888_911|132|437|568|UNK2, 888_911|117|176|292|UNK1, 911|335|569|903|PF12698.7

Primary: sp|P61011|SRP54_HUMAN Signal recognition particle 54 kDa protein OS=Homo sapiens OX=9606 GN=SRP54 PE=1 SV=1

258_504|195|102|296|PF00448.22, 850_504|106|326|431|PF02978.19, 504|78|6|83|PF02881.19, 504|73|432|504|UNK1

Secondary: sp|P0AGD7|SRP54_ECOLI Signal recognition particle protein OS=Escherichia coli (strain K12) OX=83333 GN=ffh PE=1 SV=1

850_453|99|329|427|PF02978.19, 453|78|5|82|PF02881.19, 453|197|100|296|PF00448.22, 453|32|297|328|UNK1

Secondary: sp|P10121|FTSY_ECOLI Signal recognition particle receptor FtsY OS=Escherichia coli (strain K12) OX=83333 GN=ftsY PE=1 SV=1

258_497|201|293|493|PF00448.22, 497|66|210|275|PF02881.19, 497|209|1|209|UNK1

Primary: sp|P28331|NDUS1_HUMAN NADH-ubiquinone oxidoreductase 75 kDa subunit, mitochondrial OS=Homo sapiens OX=9606 GN=NDUFS1 PE=1 SV=3

622_727|148|153|300|UNK2, 666_727|328|301|628|PF00384.22, 731_727|40|113|152|PF10588.9, 807_727|75|32|106|PF13510.6, 727|53|658|710|PF09326.11, 727|31|1|31|UNK1

Secondary: sp|P07658|FDHF_ECOLI Formate dehydrogenase H OS=Escherichia coli (strain K12) OX=83333 GN=fdhF PE=1 SV=2

666_715|427|61|487|PF00384.22, 715|53|1|53|PF04879.16, 715|107|576|682|PF01568.21, 715|88|488|575|UNK1, 715|33|683|715|UNK2

Secondary: sp|P09152|NARG_ECOLI Respiratory nitrate reductase 1 alpha chain OS=Escherichia coli (strain K12) OX=83333 GN=narG PE=1 SV=4

666_1247|726|108|833|PF00384.22, 1247|38|3|40|PF14710.6, 1247|116|1087|1202|PF01568.21, 1247|67|41|107|UNK1, 1247|253|834|1086|UNK2, 1247|45|1203|1247|UNK3

Secondary: sp|P18775|DMSA_ECOLI Dimethyl sulfoxide reductase DmsA OS=Escherichia coli (strain K12) OX=83333 GN=dmsA PE=1 SV=2

666_814|458|119|576|PF00384.22, 814|61|56|116|PF04879.16, 814|112|695|806|PF01568.21, 814|55|1|55|UNK1, 814|118|577|694|UNK2

Secondary: sp|P19319|NARZ_ECOLI Respiratory nitrate reductase 2 alpha chain OS=Escherichia coli (strain K12) OX=83333 GN=narZ PE=1 SV=5

666_1246|725|108|832|PF00384.22, 1246|38|3|40|PF14710.6, 1246|114|1086|1199|PF01568.21, 1246|67|41|107|UNK1, 1246|253|833|1085|UNK2, 1246|47|1200|1246|UNK3

Secondary: sp|P20099|BISC_ECOLI Biotin sulfoxide reductase OS=Escherichia coli (strain K12) OX=83333 GN=bisC PE=1 SV=3

666_777|462|56|517|PF00384.22, 777|41|12|52|PF18364.1, 777|103|637|739|PF01568.21, 777|119|518|636|UNK1, 777|38|740|777|UNK2

Secondary: sp|P24183|FDNG_ECOLI Formate dehydrogenase, nitrate-inducible, major subunit OS=Escherichia coli (strain K12) OX=83333 GN=fdnG PE=1 SV=3

666_1015|483|107|589|PF00384.22, 1015|61|44|104|PF04879.16, 1015|116|893|1008|PF01568.21, 1015|43|1|43|UNK1, 1015|303|590|892|UNK2

Secondary: sp|P32176|FDOG_ECOLI Formate dehydrogenase-O major subunit OS=Escherichia coli (strain K12) OX=83333 GN=fdoG PE=1 SV=5

666_1016|484|107|590|PF00384.22, 1016|60|44|103|PF04879.16, 1016|116|894|1009|PF01568.21, 1016|43|1|43|UNK1, 1016|303|591|893|UNK2

Secondary: sp|P33602|NUOG_ECOLI NADH-quinone oxidoreductase subunit G OS=Escherichia coli (strain K12) OX=83333 GN=nuoG PE=1 SV=4

622_908|96|125|220|UNK1, 731_908|37|88|124|PF10588.9, 807_908|76|3|78|PF13510.6, 908|54|221|274|PF04879.16, 908|77|562|638|PF00384.22, 908|63|828|890|PF01568.21, 908|287|275|561|UNK2, 908|189|639|827|UNK3

Secondary: sp|P33937|NAPA_ECOLI Periplasmic nitrate reductase OS=Escherichia coli (strain K12) OX=83333 GN=napA PE=1 SV=3

666_828|472|96|567|PF00384.22, 828|54|39|92|PF04879.16, 828|109|714|822|PF01568.21, 828|38|1|38|UNK1, 828|146|568|713|UNK2

Secondary: sp|P77374|YNFE_ECOLI Putative dimethyl sulfoxide reductase chain YnfE OS=Escherichia coli (strain K12) OX=83333 GN=ynfE PE=1 SV=1

666_808|456|111|566|PF00384.22, 808|60|49|108|PF04879.16, 808|114|687|800|PF01568.21, 808|48|1|48|UNK1, 808|120|567|686|UNK2

Secondary: sp|P77561|YDEP_ECOLI Protein YdeP OS=Escherichia coli (strain K12) OX=83333 GN=ydeP PE=2 SV=1

666_759|316|108|423|PF00384.22, 759|107|1|107|UNK1, 759|336|424|759|UNK2

Secondary: sp|P77783|YNFF_ECOLI Probable dimethyl sulfoxide reductase chain YnfF OS=Escherichia coli (strain K12) OX=83333 GN=ynfF PE=1 SV=4

666_807|452|114|565|PF00384.22, 807|60|52|111|PF04879.16, 807|114|686|799|PF01568.21, 807|51|1|51|UNK1, 807|120|566|685|UNK2

Primary: sp|Q9UG63|ABCF2_HUMAN ATP-binding cassette sub-family F member 2 OS=Homo sapiens OX=9606 GN=ABCF2 PE=1 SV=2

364_623|157|101|257|PF00005.27, 377_623|133|413|545|PF00005.27, 814_623|38|258|295|UNK2, 833_623|78|546|623|UNK4, 623|73|296|368|PF12848.7, 623|100|1|100|UNK1, 623|44|369|412|UNK3

Secondary: sp|P0A9U3|YBIT_ECOLI Uncharacterized ABC transporter ATP-binding protein YbiT OS=Escherichia coli (strain K12) OX=83333 GN=ybiT PE=1 SV=1

833_530|63|468|530|UNK2, 530|167|18|184|PF00005.27, 530|86|224|309|PF12848.7, 530|132|336|467|PF00005.27, 530|39|185|223|UNK1

Secondary: sp|P63389|YHES_ECOLI Uncharacterized ABC transporter ATP-binding protein YheS OS=Escherichia coli (strain K12) OX=83333 GN=yheS PE=1 SV=1

364_637|162|17|178|PF00005.27, 377_637|132|328|459|PF00005.27, 612_637|82|217|298|PF12848.7, 814_637|38|179|216|UNK1, 637|178|460|637|UNK2

Primary: sp|P11388|TOP2A_HUMAN DNA topoisomerase 2-alpha OS=Homo sapiens OX=9606 GN=TOP2A PE=1 SV=3

508_1531|459|713|1171|PF00521.20, 601_1531|102|457|558|PF01751.22, 800_1531|146|79|224|PF02518.26, 1531|161|266|426|PF00204.25, 1531|139|573|711|PF16898.5, 1531|88|1435|1522|PF08070.11, 1531|78|1|78|UNK1, 1531|41|225|265|UNK2, 1531|30|427|456|UNK3, 1531|263|1172|1434|UNK4

Secondary: sp|P0AES4|GYRA_ECOLI DNA gyrase subunit A OS=Escherichia coli (strain K12) OX=83333 GN=gyrA PE=1 SV=1

508_875|475|32|506|PF00521.20, 875|31|1|31|UNK1, 875|31|507|537|UNK2, 875|36|840|875|UNK3

Secondary: sp|P0AES6|GYRB_ECOLI DNA gyrase subunit B OS=Escherichia coli (strain K12) OX=83333 GN=gyrB PE=1 SV=2

601_804|113|419|531|PF01751.22, 800_804|143|32|174|PF02518.26, 804|170|221|390|PF00204.25, 804|166|564|729|PF18053.1, 804|62|732|793|PF00986.21, 804|31|1|31|UNK1, 804|46|175|220|UNK2, 804|32|532|563|UNK3

Secondary: sp|P0AFI2|PARC_ECOLI DNA topoisomerase 4 subunit A OS=Escherichia coli (strain K12) OX=83333 GN=parC PE=1 SV=1

508_752|437|29|465|PF00521.20, 752|127|466|592|UNK1, 752|77|676|752|UNK2

Secondary: sp|P20083|PARE_ECOLI DNA topoisomerase 4 subunit B OS=Escherichia coli (strain K12) OX=83333 GN=parE PE=1 SV=3

601_630|109|413|521|PF01751.22, 800_630|144|28|171|PF02518.26, 630|167|218|384|PF00204.25, 630|65|556|620|PF00986.21, 630|46|172|217|UNK1, 630|34|522|555|UNK2

Primary: sp|Q86XP3|DDX42_HUMAN ATP-dependent RNA helicase DDX42 OS=Homo sapiens OX=9606 GN=DDX42 PE=1 SV=1

206_938|172|277|448|PF00270.29, 415_938|106|488|593|PF00271.31, 938|276|1|276|UNK1, 938|39|449|487|UNK2, 938|345|594|938|UNK3

Secondary: sp|P0A8J8|RHLB_ECOLI ATP-dependent RNA helicase RhlB OS=Escherichia coli (strain K12) OX=83333 GN=rhlB PE=1 SV=2

415_421|109|243|351|PF00271.31, 421|174|33|206|PF00270.29, 421|32|1|32|UNK1, 421|36|207|242|UNK2, 421|70|352|421|UNK3

Secondary: sp|P25888|RHLE_ECOLI ATP-dependent RNA helicase RhlE OS=Escherichia coli (strain K12) OX=83333 GN=rhlE PE=1 SV=3

206_454|172|25|196|PF00270.29, 420_454|99|241|339|PF00271.31, 454|44|197|240|UNK1, 454|115|340|454|UNK2

Primary: sp|P08622|DNAJ_ECOLI Chaperone protein DnaJ OS=Escherichia coli (strain K12) OX=83333 GN=dnaJ PE=1 SV=3

225_376|213|118|330|PF01556.18, 489_376|63|5|67|PF00226.31, 376|50|68|117|UNK1, 376|46|331|376|UNK2

Secondary: sp|O75953|DNJB5_HUMAN DnaJ homolog subfamily B member 5 OS=Homo sapiens OX=9606 GN=DNAJB5 PE=1 SV=1

489_348|62|4|65|PF00226.31, 348|159|172|330|PF01556.18, 348|106|66|171|UNK1

Secondary: sp|Q96EY1|DNJA3_HUMAN DnaJ homolog subfamily A member 3, mitochondrial OS=Homo sapiens OX=9606 GN=DNAJA3 PE=1 SV=2

225_480|204|210|413|PF01556.18, 480|63|93|155|PF00226.31, 480|92|1|92|UNK1, 480|54|156|209|UNK2, 480|67|414|480|UNK3

Secondary: sp|Q9UBS3|DNJB9_HUMAN DnaJ homolog subfamily B member 9 OS=Homo sapiens OX=9606 GN=DNAJB9 PE=1 SV=1

489_223|61|27|87|PF00226.31, 223|136|88|223|UNK1

Secondary: sp|Q9UDY4|DNJB4_HUMAN DnaJ homolog subfamily B member 4 OS=Homo sapiens OX=9606 GN=DNAJB4 PE=1 SV=1

489_337|62|4|65|PF00226.31, 337|159|161|319|PF01556.18, 337|95|66|160|UNK1
